# New insight into HCV E1/E2 region of genotype 4a

**DOI:** 10.1186/s12985-014-0231-y

**Published:** 2014-12-30

**Authors:** Nehal Hussein, Abdel-Rahman N Zekri, Mohamed Abouelhoda, Hanaa M Alam El-din, Ahmed Abdelwahab Ghamry, Mahmoud A Amer, Ghada M sherif, Abeer A Bahnassy

**Affiliations:** Virology and Immunology Unit, Cancer Biology Department, National Cancer Institute, Cairo University, Fom El-Khalig, Cairo, 11796 Egypt; Faculty of Engineering, Cairo University, Giza, Egypt; Faculty of Science, Zoology Department, Cairo University, Giza, Egypt; Biostatistic & Epidemiology Department, National Cancer Institute, Cairo University, Cairo, Egypt; Center for Informatics Sciences, Nile University, Giza, Egypt; Pathology Department, National Cancer Institute, Cairo University, Cairo, Egypt

**Keywords:** HCV, Genotype 4, Variability, Molecular clock

## Abstract

**Introduction:**

Hepatitis C virus (HCV) genome contains two envelope proteins (E1 and E2) responsible for the virus entry into the cell. There is a substantial lack of sequences covering the full length of E1/E2 region for genotype 4. Our study aims at providing new sequences as well as characterizing the genetic divergence of the E1/E2 region of HCV 4a using our new sequences along with all publicly available datasets.

**Methods:**

The genomic segments covering the whole E1/E2 region were isolated from Egyptian HCV patients and sequenced. The resulting 36 sequences 36 were analyzed using sequence analysis techniques to study variability within and among hosts in the same time point. Furthermore, previously published HCV E1/E2 sequence datasets for genotype 4a were retrieved and categorized according to the geographical location and date of isolation and were used for further analysis of variability among Egyptian over a period of 15 years, also compared with non-Egyptian sequences to figure out region-specific variability.

**Results:**

Phylogenetic analysis of the new sequences has shown variability within the host and among different individuals in the same time point. Analysis of the 36 sequences along with the Egyptian sequences (254 sequences in E1 in the period from 1997 to 2010 and 8 E2 sequences in the period from 2006 to 2010) has shown temporal change over time. Analysis of the new HCV sequences with the non-Egyptian sequences (182 sequences in E1 and 155 sequences in the E2) has shown region specific variability. The molecular clock rate of E1 was estimated to be 5E-3 per site per year for Egyptian and 5.38E-3 for non-Egyptian. The clock rate of E2 was estimated to be 8.48E per site per year for Egyptian and 6.3E-3 for non-Egyptian.

**Conclusion:**

The results of this study support the high rate of evolution of the Egyptian HCV genotype 4a. It has also revealed significant level of genetic variability among sequences from different regions in the world.

**Electronic supplementary material:**

The online version of this article (doi:10.1186/s12985-014-0231-y) contains supplementary material, which is available to authorized users.

## Background

Hepatitis C Virus (HCV) is a major risk factor for liver diseases and hepatocellular carcinomas (HCC) worldwide [1]. Approximately, 175 million persons, representing 3% of the whole world population, are affected worldwide. HCV infection is now endemic in many countries. Egypt has the highest prevalence of HCV worldwide; where 6% to 20% of the Egyptian population are HCV positive with an average of 13.8% [2].

HCV, which is a member of the Hepacivirus group of the Flaviviridae family, is an enveloped virus with a single stranded positive sense RNA genome [3]. The HCV genome encodes a single poly-protein that is cleaved into 10 mature proteins. The structural proteins are located near the 5' end of the poly-protein and the non-structural proteins are located near the 3' end [4]**.**

HCV isolates have been classified into seven genotypes and several subtypes, differing in about 20–25% of the nucleotide sequences [5,6]. HCV has a very high mutation rate of about 10E-5 error/nt, with large genetic heterogeneity and variability due to the lack of any proofreading mechanism [7]. The geographical distribution of genotypes and subtypes differs greatly from one region to another [8]. In the Middle East and Africa, HCV genotype 4 (HCV-4) is the most common genotype. In Egypt, genotype 4 is the most predominant genotype, and 4a is the dominant subtype [10]. Interestingly, Genotype 4 has recently spread to several European countries [9].

A pool of phylogenetic- related viral quasispecies exists in the blood of infected persons due to the high mutation rate. The genomes of these quasispecies vary slightly and fluctuate during the course of the disease. However, the changes in the consensus sequence of a viral population occur only if the population equilibrium is altered by a selection mechanism. As discussed in [11], the viral variants may be related to differences in transmissibility, immunogenicity, or pathogenicity, which should be taken into consideration in the development of prophylactic and therapeutic vaccines.

The E1/E2 region harbors the E1 and E2 glycoproteins, which are thought to be the viral attachment proteins. This region demonstrates a high level of variability, especially at two sub-regions known as HVR1 and HVR2 [12]. This variability is thought to be related either to the non-clearance of the virus or the resistance to antiviral therapies [13]. This variability also poses a major challenge in vaccine design, where the objective is the identification of protective epitopes conserved across different strains of HCV [14] [15].

Despite the importance of HCV E1/E2 region, very few studies have sequenced the full length of the related sequence, especially for genotype 4a. From our data retrieval work described in this paper, there are only 8 Egyptian Genotype 4a sequences covering the E1/E2 region. The other Egyptian sequences cover only fraction of the region. For non-Egyptian Genotype 4a sequences, there are only 6 sequences covering the E1/E2 region and the other ones cover only a fraction of the region. There is also no study that analyzed all these sequences and compared them to each other to identify temporal as well as region specific variability.

In this paper, we have focused on the E1/E2 region and studied its variability among Egyptian and non-Egyptian isolates. Specifically, 1) we sequenced 36 viral genome segments from five patients with HCV genotype 4a covering the E1/E2 region (of about 1,672 nucleotides), 2) we collected all publically available sequences covering E1/E2 genotype 4a and compared them to each other and to our new sequences considering time of isolation and geographical location, and 3) we estimated the rate of change within the Egyptian and Non-Egyptian isolates.

## Methods

### Patients

The present study included five different HCV (genotype-4a)-infected individuals with no history of liver cirrhosis or end stage liver disease. A written informed consent was obtained from each patient prior to enrollment in the study and the ethical committees of Kasr el Ainy, School of Medicine and the National Cancer institute, Cairo University approved the study protocol which conformed to the ethical guidelines of the World Medical Association Declaration of Helsinki. Serum samples were collected from patients admitted to Viral Hepatitis Center. Anti-HCV antibodies were detected in sera by fourth-generation ELISA (ETI-AB-HCVK-4, DiaSorin) and infection was confirmed by HCV RT-PCR. All samples were genotyped with Versant HCV genotype assay (LiPA) 2.0 (Innogenetics, Siemens Healthcare Diagnostics, USA) prior to enrollment in the study.

### RNA extraction and amplification of the E1/E2 genome regions

Viral RNA was extracted from 140 μL of serum using the Qiagen vRNA Extraction Kit according to manufacturer’s protocol (Qiagen, Hilden, Germany). c-DNA was synthesized using Reverse transcription step Kit (StrataScript® Reverse Transcription- Stratagene, La Jolla, CA). PCR amplification was done using the primer sequences and PCR conditions (a nested PCR) of Dimitri et al. [16] (Table 1)**.** PCR cycling conditions were as follows: 95°C for 2 min, followed by 35 cycles of 30 s at 95°C, 30 s at 50°C, 2 min at 68°C and a final extension of 68°C for 10 min using the HotStart-IT™ Taq DNA Polymerase (USA). A second round of PCR was carried out under the same conditions with 1 μl of the first PCR product for 28 cycles and then the PCR products were separated by 1% agarose gel electrophoresis. Fragments of the expected size (1.7 kb) were purified out of the gel, for subsequent cloning; using a High pure PCR product purification kit (Roche applied science, Mannheim, Germany).

**Table 1:** **Primers used for the generation of E1/E2 area of HCV genotype 4**

**Primers**	**Polarity**	**Sequences (5′-3′)**	**Application**	**Product size**
HCV outer 2	Antisense	CACCAGCGGGTGAAGCAGCATTGA	RT/1^st^ round PCR	
HCV outer 1	Sense	GGACGGGGTAAACTATGCAACAGG	1st round PCR	1804 bp
HCV inner 2	Antisense	GACAGTTACGCCTGAACTTGACTTACCATAAACATC	2nd round PCR	
HCV inner 1	Sense	CACCCATGGGTTGCTCTTTTTCTATC	2nd round PCR	1726 bp

**Table 2:** **Maximum likelihood estimate of substitution matrix, substitution pattern and rates were estimated under the Tamura-Nei (1993) model**

	**A**	**T/U**	**C**	**G**
**A**	-	*3.48*	*3.91*	**16.09**
**T/U**	*2.75*	-	**23.29**	*3.52*
**C**	*2.75*	**20.73**	-	*3.52*
**G**	**12.56**	*3.48*	*3.91*	-

Rates of different transitional substitutions are shown in bold and those of transversionsal substitutions are shown in *italics*.

### Cloning

Purified PCR products were directly ligated into pCR2.1- TOPO plasmid (Invitrogen, Carlsbad, CA, USA) and then chemically transformed into One Shot Top10 Escherichia coli (Invitrogen, Carlsbad, CA, USA) according to manufacturer’s instructions. Bacteria were plated onto Luria Bertani agar plates containing ampicillin (50–100 μg/ml). Clones with E1/E2 sequences were identified by standard blue/white screening, as well as by colony PCR and restriction enzyme cut. {We used restriction enzyme maps and applied applying the remap tool from the European bank of bionformatics (http://srs.ebi.ac.uk/srsbin/cgi-bin/wgetz) to the sequences of the vector and E1/E2 region. Hind III (GibcoBRL, USA) was used for restriction enzyme cut, with specific one target position at 234–240 bp of the vector sequence map, according to the manufacture’s protocol. Then, 10 μl of the digestion reaction were loaded onto 1% agarose gel, fragments were separated by electrophoresis and visualized by ethidium bromide staining}.

### Sequencing

Sequencing of purified plasmids was performed using the BigDye Terminator kit (Applied Biosystems, Foster City, Calif.) according to manufacturer’s instructions in ABI 310 automatic sequencer (company and country). Plasmids containing E1/E2 inserts were sequenced using a bidirectional primer walking method and sequences were analyzed with Lasergene software (DNAStar, Inc., Madison, WI). On average, ten clones were sequenced for each clinical isolate. All reads were checked for vector contamination and assembled into contiguous sequences using the tools from the EMBOSS package [17].

### Deposition in GenBank

All the E1/E2 sequences characterized in the present study have been submitted to GenBank under the indicated accession numbers **JX310279-JX310314**.

### Data collection from public databases

Three main databases were queried to retrieve the publicly available HCV sequences related to the E1/E2 region:

1. NCBI GenBank (https://www.ncbi.nlm.nih.gov/genbank), which is the major repository for nucleotide sequences.
2. The Hepatitis C Virus Databases at LANL, Los Alamos National Library (hcv.lanl.gov).
3. The European Hepatitis C Virus Database euHCVdb (https://euhcvdb.ibcp.fr).

The retrieval and categorization of sequences from LANL and euHCVdb was based on filtering the deposited HCV sequences according to the genotype. Determining whether a sequence is from Egypt or not and determining its collection date were achieved by parsing the respective GenBank file. In case of using the GenBank database, the retrieval and categorization of sequences was achieved by the following workflow. First, all HCV sequences were collected in GenBank file formats. These files were then parsed to filter out sequences not including Genotype 4a. Also the filers including regions other than E1/E2 were filtered out. Whole genomes with Genotype 4a are always accepted as they include E1/E2 regions. The remaining files (sequences) are then parsed again to categorize them according to the location and the date of isolation. In case of missing information, we restored to the related publications to complete the categorization. The retrieval and categorization were accomplished using own scripts written in Python and Perl scripting languages.

### Sequence analysis

Multiple sequence alignment was accomplished using the Clustalw [18] and the MUSCLE [19] programs. We used two distance measures for aligning pairs of sequences: the k-mer distance (for an unaligned pair) and the Kimura distance (for an aligned pair). We wrote a program (in Perl) to correct for sequencing errors; in this program a single character change in a column was considered as a sequencing error and it was corrected.

### Preprocessing of public datasets

Except for whole genome sequences, the public sequences cover parts of the E1/E2 region. To extract the segments common to all sequences, we used the following workflow: First, we aligned the whole genomic sequences along with our full length E1/E2 sequences using ClustalW. Then we extracted the region covering E1/E2 from each whole genome sequence. For the E1 region, we aligned the other partial public sequences to the E1 sequences obtained from the previous step and visualized the results using the program JalView. From JalView, which supports selection and editing of multiple sequence alignments, we selected the part of E1 common to all given sequences and extracted it to multi-Fasta file. The same steps were conducted for pre-processing E2 public sequences.

### Phylogenetic trees

Phylogenetic trees were constructed with the PhyML package using default parameters. The Akaike’s information criterion, implemented in JModeltest [20]**,** was used to find the most appropriate model of evolution. We also used the BEAST program [21] to compute the phylogenetic tree under a molecular clock assumption. We labeled each sequence with its date and used the *strict* and *relaxed uncorrelated lognormal* clock models. The chain length for the Monte Carlo Markov Chains (MCMC) procedure was set to 20 millions, at least, in order to achieve a statistically significant results with an effective sample size (ESS) larger than 100. Data Visualization was achieved using the FigTree (http://tree.bio.ed.ac.uk/software/figtree/) and Cytoscape programs [22].

### Variability analysis

The Nei and Gojobori method [23] implemented in the molecular evolutionary genetics analysis software package (MEGA, version 5.0) [24] was used to determine the genetic distance (d), the number of synonymous substitutions per synonymous site (dS), the number of non-synonymous substitutions per nonsynonymous site (dN) and dN/dS values.

To spot the variability within the highly variable regions of the HCV E1/E2, we used the VarPlot software tool (available from S.C.R. at http://sray.med.som.jhmi.edu/SCRoftware) [25]**,** which is also based on the Nei and Gojobori method [23]**.** Accordingly, the values for dS, dN and the dN/dS ratio were computed in sliding w ws over the multiple alignments; each has a width of 20 nucleotides [25]. Two consecutive windows were overlapping by 19 nucleotides. At each step, the results for all pairwise comparisons were computed and the values were averaged. The Jukes-Cantor correction was used to correct for underestimated distances due to multiple substitutions at the same site [26].

## Results

### 1-Analysis of sequencing data

After the assembly and annotation, we excluded all clones containing nonsense and frame shift mutations as well as the sequences that didn’t overlap well or didn’t meet the control criteria. Accordingly, the number of the analyzed variants was reduced to 36 out of 50, which represents isolates from five different HCV patients. Our dataset of 36 sequences were assembled into contiguous sequences of about 1762 bp.

### 2-Alignment of informative sites

Multiple sequence alignment was computed using the Clustalw and Muscle programs. The resulting two alignments were compared and curated where a single character change in a column was considered as a sequencing error and was corrected. This curation has removed a number of errors and it fine-tuned the final analysis results. Multiple sequence alignment(s) of the sequences is available in Additional file 1.

### 3-Phylogenetic analysis of the 36 Egyptian E1/E2 sequences

**Figure 1:**
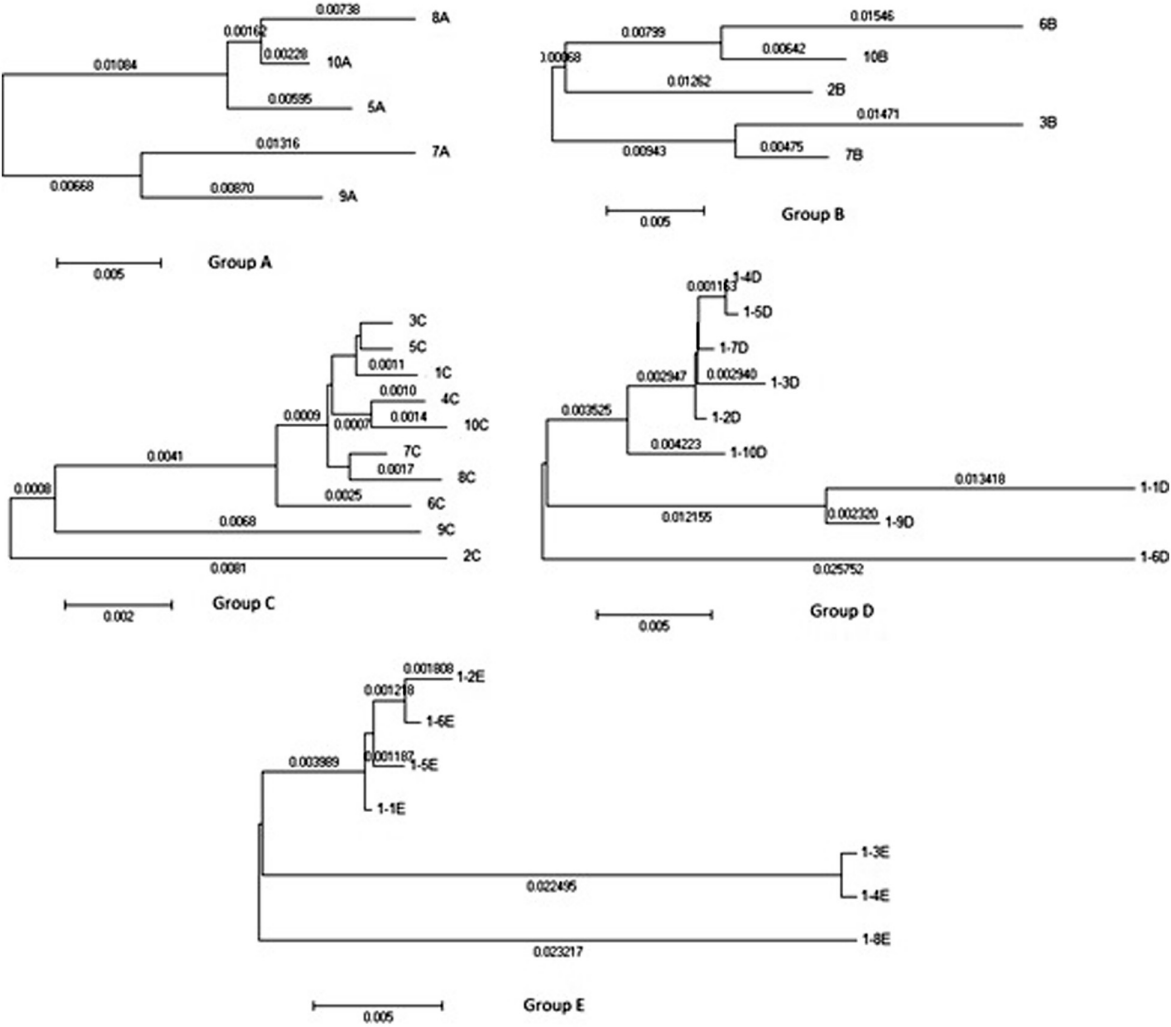
**Predicted phylogenetic relationships for each group of sequences within the same infected individual, the scale bar represents genetic distance (substitutions per site).** Group B represent the most closely related isolates. All panels represents different patients marked as A, B, C, D and E.

**Figure 2:**
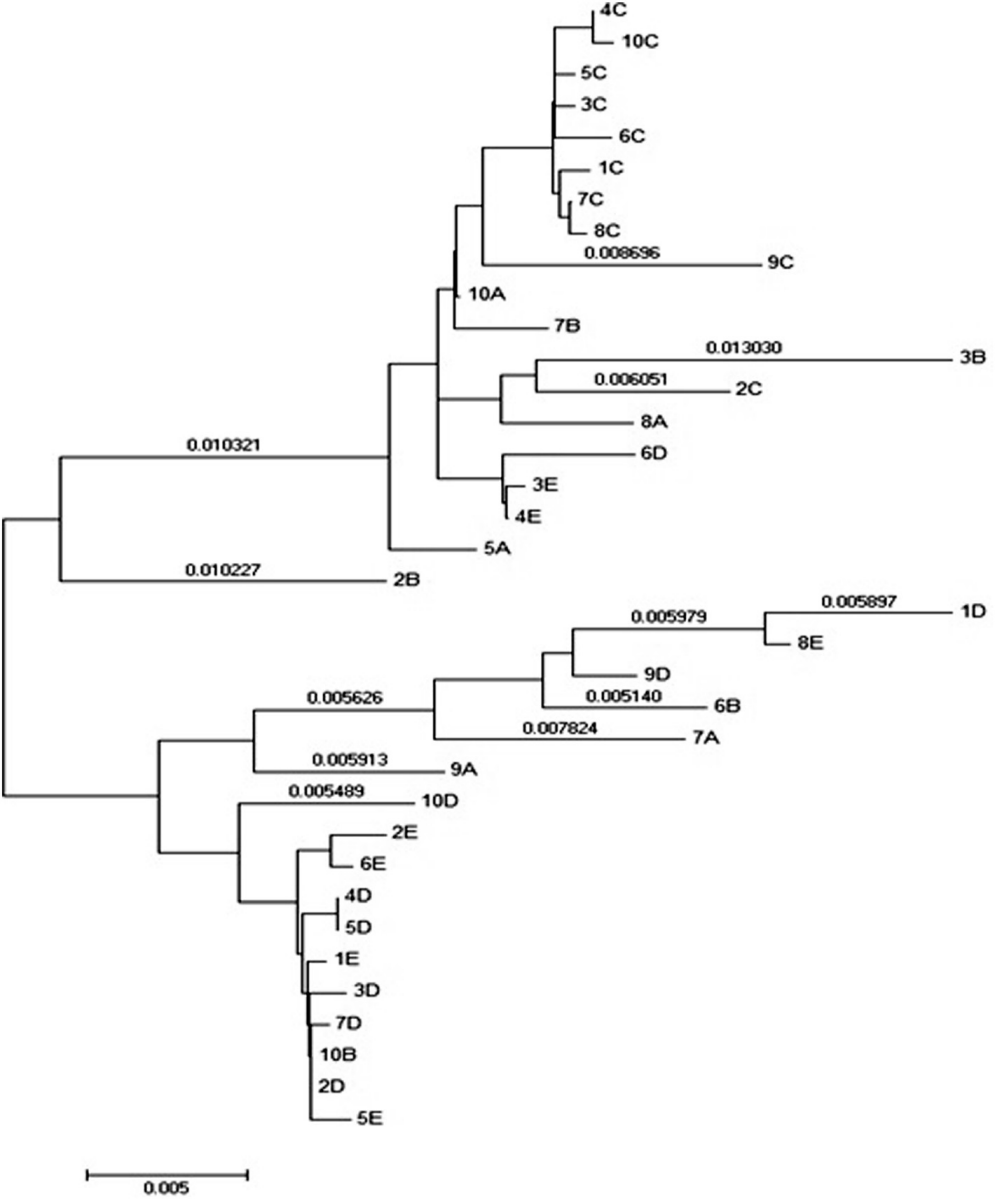
**Predicted phylogenetic relationships among HCV genotype 4 isolates of E1/E2 region.** The scale bar represents genetic distance (substitutions per site).

Phylogenetic trees were constructed for all sequences to determine the diversity of variants constituting each individual HCV population and the relationships between them, using HCV genotype 1 sequence as the out-species. However, to enlarge the image, the out-species was deleted from the trees in the figures, without changes in the topology of the representations. The analyzed sequences represented 36 different isolates form 5 different patients, which were numbered from 1–10 and named (grouped or categorized) as: A, B, C, D, and E. Then, we constructed a phylogenetic tree for each group of sequences, which was obtained from the same individual (Figure 1)**.** For all defined sequences in the phylogenetic tree of E1/E2 region of the HCV genotype 4 sequences (36), two different monophyletic groups were observed. Each of these groups includes sequences from the different studied cases. One group includes sequences that represent all of the 5 patients and the other group includes the sequence from 4 patients only. All sequences obtained from patient C showed high genetic similarity, other clades showed remarkable differences between the sequences obtained from other patients with high degree of variability and divergence among sequences in the same individual (Figure 2).

To further assess the variability within the sequences and the reliability of the constructed tree, the maximum likelihood E estimation of substitution matrix was calculated where each entry represents the probability of substitution (*r*) from one base (row) to another (column). The substitution pattern and rates were estimated under the Tamura-Nei (1993) model [24] (Table 2). The rates of different transitional substitutions are shown in bold and those of transversional substitutions are shown in *italics*. For simplicity, the sum of *r* values was considered equal to 100. The nucleotide frequencies are: A = 20.14%, T/U = 25.46%, C = 28.61% and G = 25.79%. For estimating the Maximum log (ML) values, a user-specified topology based on the computed tree was used. The ML likelihood for this computation was −4230.178. The codon positions included were 1st + 2nd + 3rd + Noncoding**.** The average evolutionary divergence, which represents the number of base substitutions per site by averaging over all sequence pairs, was estimated to be 0.023. These results suggest a high degree of genetic diversity within the E1/E2 region of HCV genotype 4a.

**Table 3:** **Data of HCV E1 and E2 genotype 4 retrieved from data bases**

**Region**	**Country**	**Info**	**Number of collected sequences**		
			**NCBI**	**LANL**	**EUHCV**
**E1**	Egypt	Collection dates range between 1997-2010	254	8	107
**E2**	Egypt	Collection date ranged between 2006-2010	8	8	7
**E1**	Not-Egypt	From Canada, France, Ireland, Pakistan, Sri Lanka, USA	182	13	120
**E2**	Not-Egypt	From Ireland, USA	155	7	101

The analysis was made using the collection date of samples. These numbers does not include our 36 sequences, where the collection date of our sequences was 2011.

### Variability analysis with the 36 Egyptian sequences

**Figure 3:**
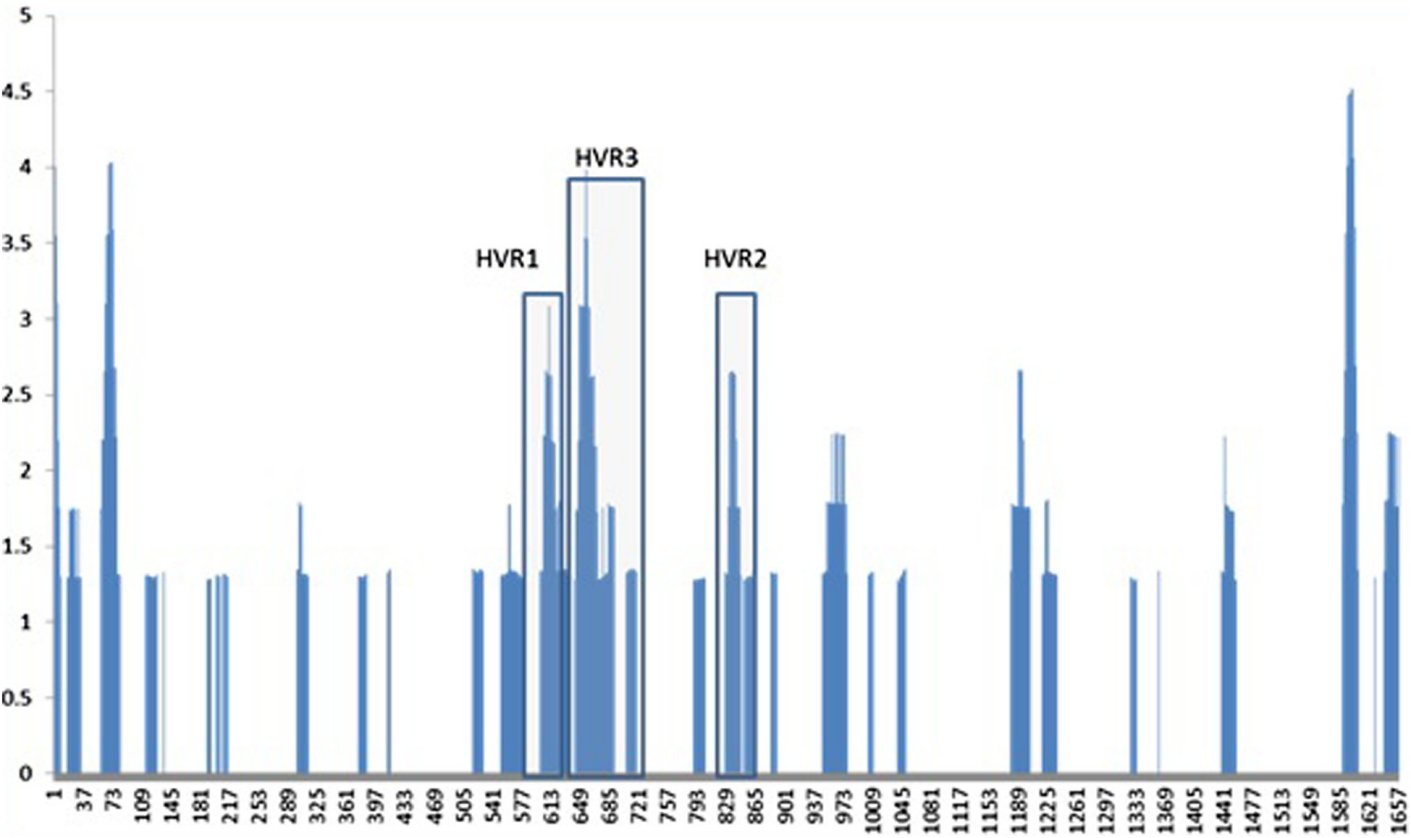
**Evolutionary rate plot of the E1/E2 region for all the nucleotide sequences.** The rate is computed using the program MEGA5. The highly variable regions HVR1, HVR2, and HVR3 are highlighted. The rate is computed using the program MEGA5 using the default parameter. Sites with rates larger than one are more variable than expected.

**Figure 4:**
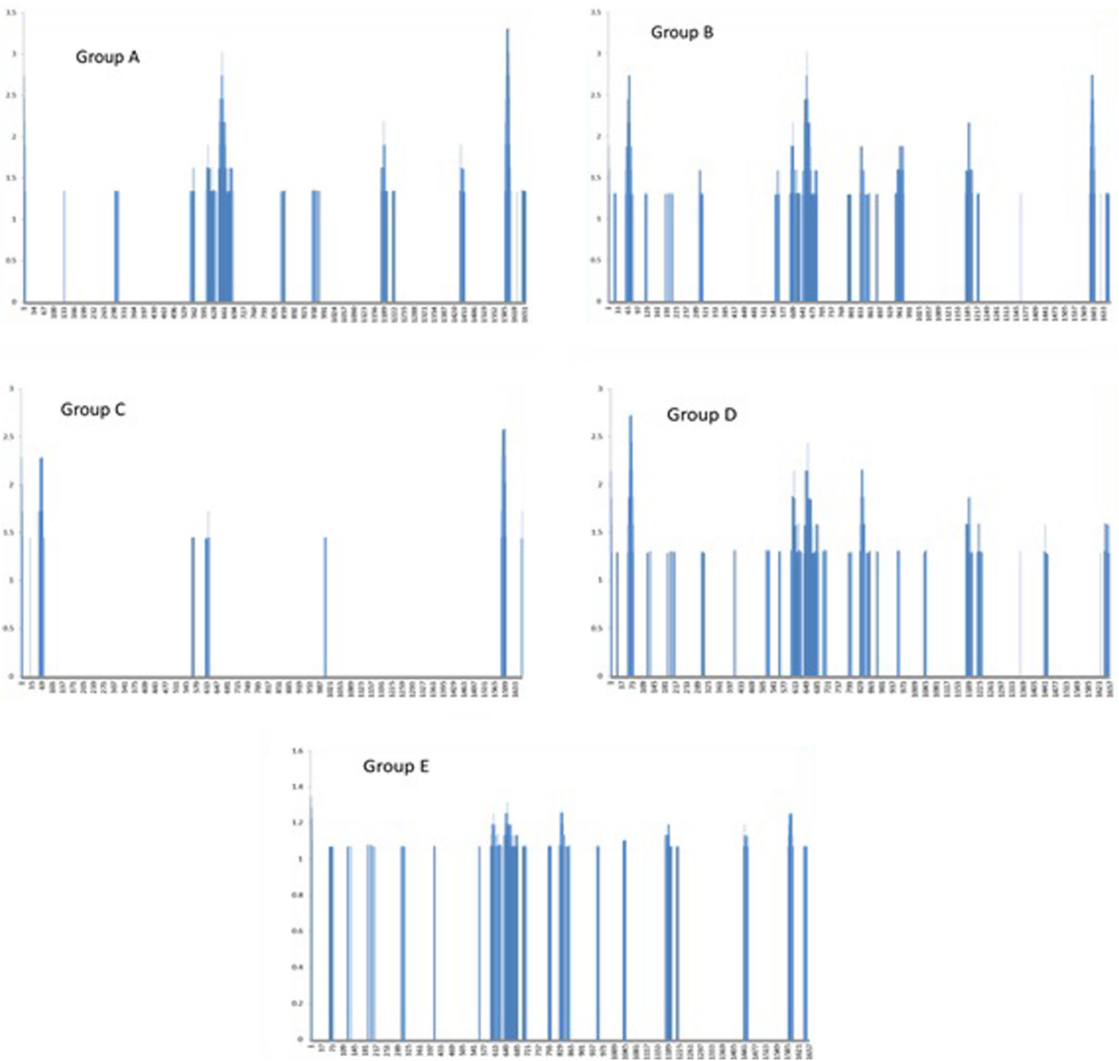
**Mean (relative) evolutionary rate plots of the E1/E2 region for each group separately.** The rate is computed using the program MEGA5 using the default parameter. Sites with rates larger than one are more variable than expected. All panels represents different patients marked as A, B, C, D and E.

To assess the variability among the different segments of our sequences, we performed a high-resolution analysis of differences in dN/dS ratios using the VarPlot. We also used the MEGA5 package to compute the mean evolutionary rates at each site. We also wrote special Perl scripts to plot these variations. Figure 3 shows the plots, which depict the evolutionary rate at each site for all groups in the dataset; we highlighted the location of the known hyper-variable regions HVR1, HVR2, and HVR3 of the E1/E2. Figure 4 shows the evolutionary rates for each group separately.

### Public HCV datasets

We retrieved all publicly available HCV genotype 4a sequences from GenBank, LANL, and euHCVdb databases. Table 3 summarizes the results of all collected sequences from these databases. Additional file 2 includes all details of the collected sequences, including the origin, the date of isolation, the number of sequences, average lengths, and the respective publications. It is clear that NCBI was the most comprehensive resource and it included more sequences than what available in other datasets. The challenge was just to develop a workflow to structure and categorize the data according to the origin of the sequences, the genotype, and the date of isolation.

In Additional file 2, we see that the E1 dataset for non-Egyptian isolated is divided into two subsets. The reason for this is as follows. Because most of the public sequences cover parts of the E1/E2 region, we used the multiple alignment based approach presented in the methodology section to find segments common to all the sequences. For E1, it was determined that some sequences cover the left most part of the E1 region and other datasets cover the right most part of the E1 region. Therefore, we composed two subsets for E1 and analyzed each one separately. The E1 dataset covering the left most part of the E1 region included 27 sequences from 6 countries: Canada, France, South Africa, Pakistan, Serilanka, and USA. The length of each sequence in the final set was 113 bp (in all the Egyptian sequences, the region starts at position 154 and ends at position 266). The second E1 set covering the rightmost part of E1 included 155 sequences from two countries (Ireland and USA). The average length of each sequence in the final set was 197 bp. (In our sequences, the region starts at position 421 and end at position 617 in each sequence.). The reason why US sequences appear in both subsets is that the US sequences are full length viral genomes including the whole E1 region. The Ireland dataset includes sequences at the junction of E1/E2 and hence they appear on the dataset covering the rightmost part of E1 and it will appear in the dataset covering E2. The E2 dataset was not sub-divided as it included the US and Ireland sequences.

### Evolution of genotype 4a in Egyptian isolates

#### Phylogenetic trees

**Figure 5:**
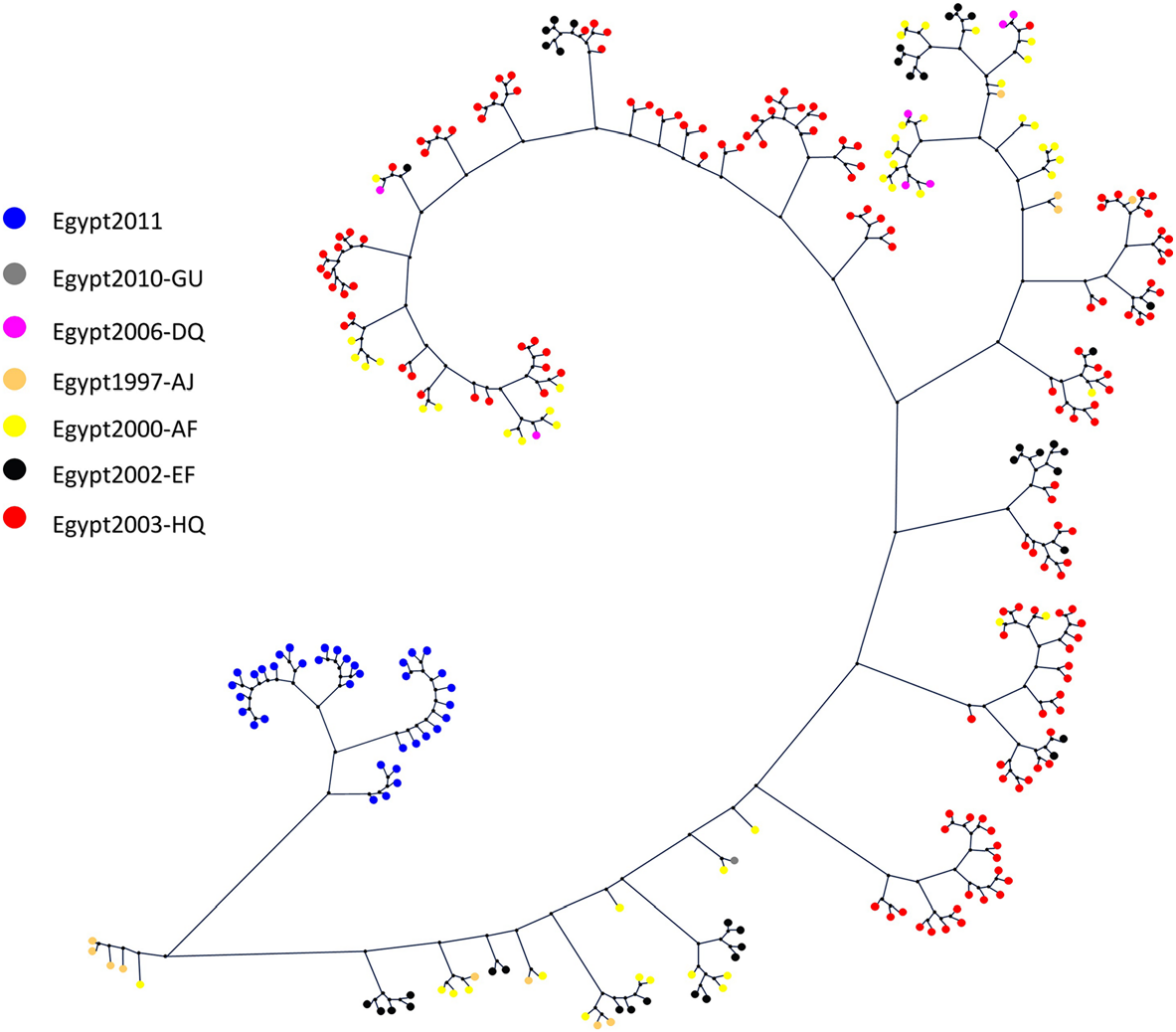
**The phylogenetic tree of the Egyptian E1 sequences isolated in the period from 1997 to 2011 and the new 36 sequences in this study.**

Figure 5 shows the phylogenetic tree for the 254 public E1 sequences (isolated from 1997 to 2010) in addition to our new ones. It is clear that our sequences are grouped together. The other largest group is for a previous study of 146 Egyptian sequences collected in 2003. It can also be observed that the sequences from 1997 also cluster together. This reflects a high evolutionary rate for the viral sequences in the E1 region.

**Figure 6:**
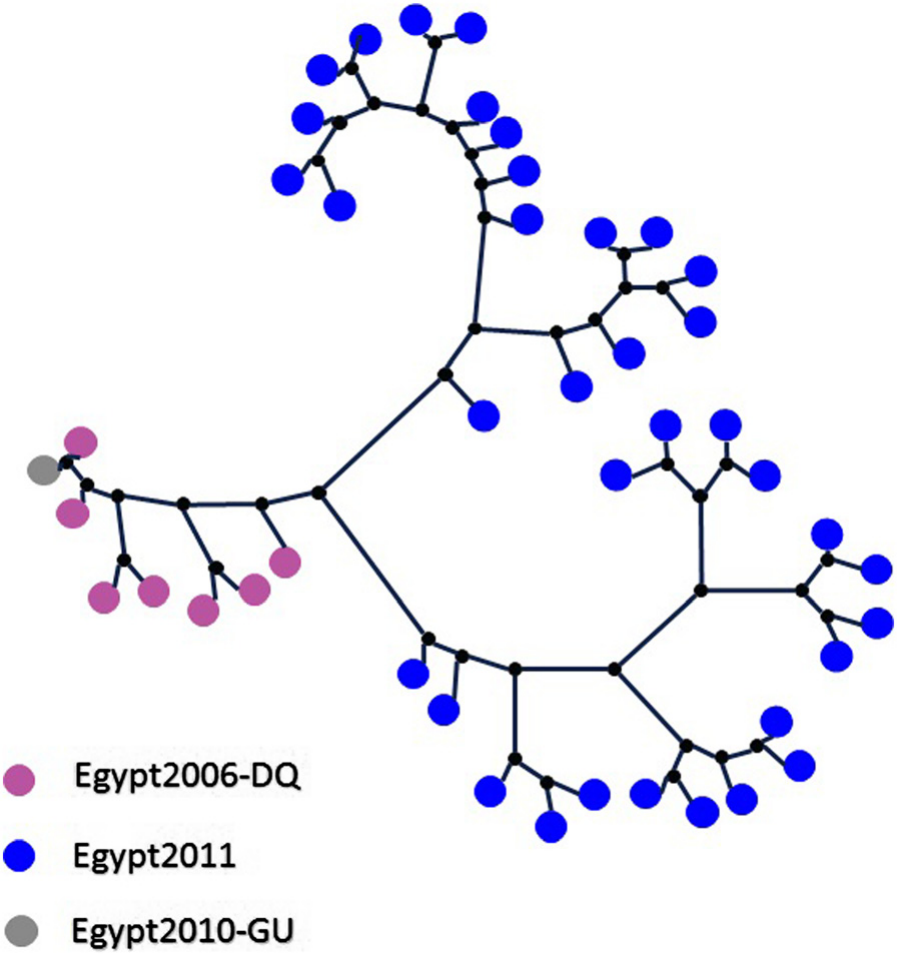
**The phylogenetic tree of the Egyptian E2 sequences isolated in the period from 2006 to 2010 and the new 36 sequences in this study.**

Figure 6 shows the phylogenetic tree for the 8 public E2 sequences isolated in 2006 and 2010 in addition to our new ones. It is also clear that our sequences are grouped together. The other sequences are clustered separately. Although the data is not large enough to yield a conclusion, it is most likely that E2 also evolves at a high rate similar to E1, especially that it host the highly variable regions.

#### Estimation of clock rates for Egyptian sequences

To evaluate the molecular clock, we labeled the sequences with the collection date and used the program BEAST. The rate of change for E1 sequences was estimated to be 5.03E-3 per site per year, and that of E2 was 8.03E-3 per site per year.

### Evolution of genotype 4a in non-Egyptian isolates

#### E1 Analysis

**Figure 7:**
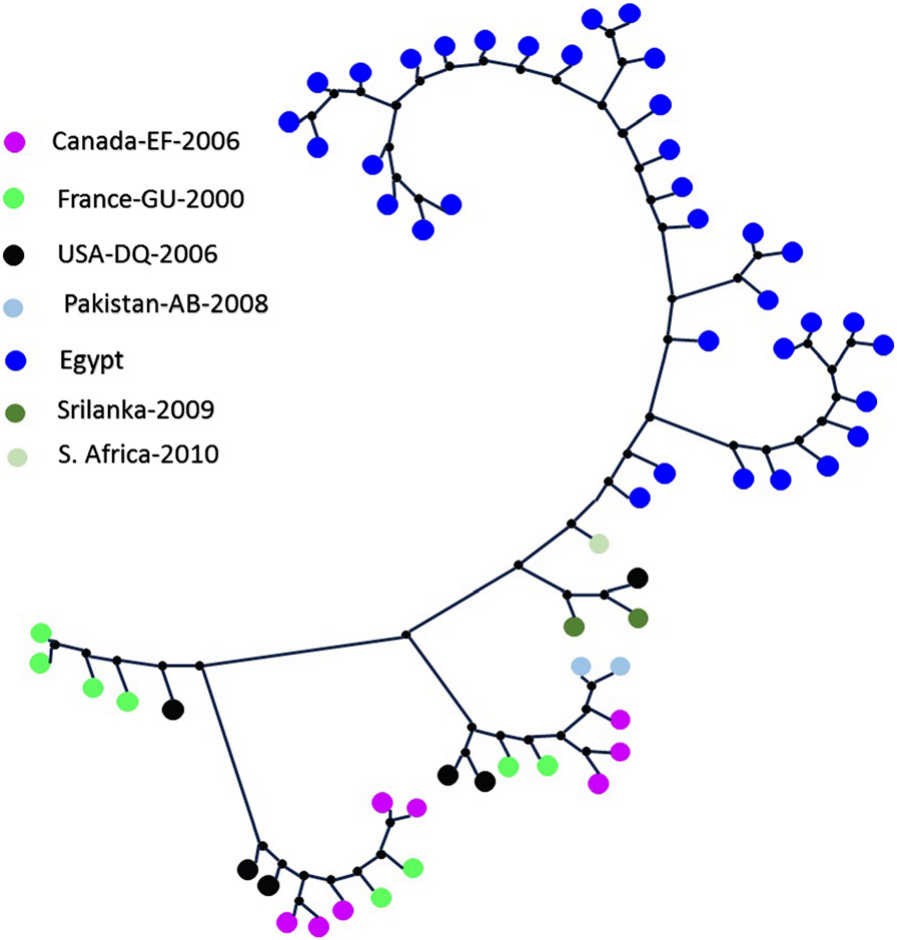
**The phylogenetic tree of the first set of E1 sequences (covering the leftmost part of E1 region) including the Egyptian sequences.**

**Figure 8:**
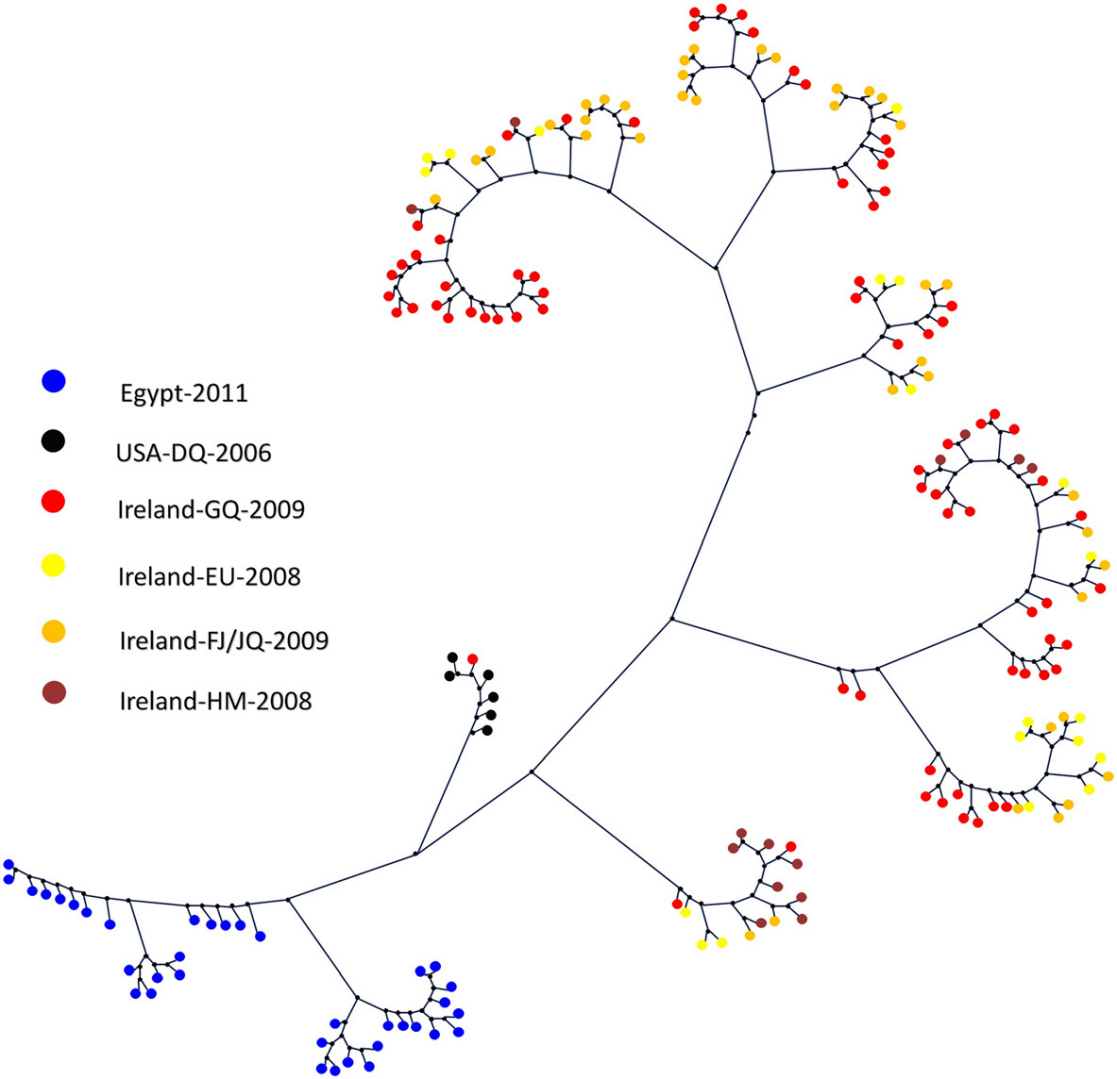
**The phylogenetic tree of the second set of E1 sequences (covering the rightmost part of E1 region) including the Egyptian sequences.**

Exactly 218 E1 sequences from different countries including our new 36 sequences were used to develop a phylogenetic tree. After arranging the input sequences, we computed the phylogeny and the rate of evolution for each set separately. Figure 7 and Figure 8 show the phylogenetic trees for the two E1 sequence sets including our new 36 sequences. It can be clearly observed that sequences from the same region cluster together in separate sub-trees.

#### E2 Analysis

**Figure 9:**
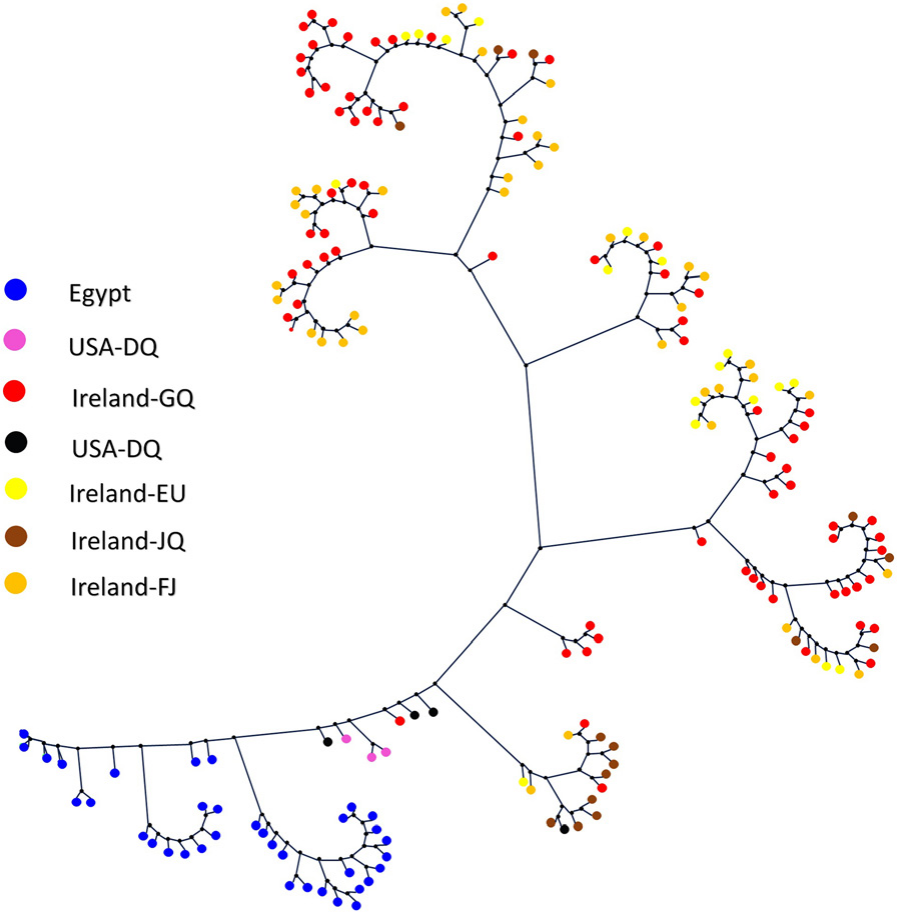
**The phylogenetic tree of the E2 sequences including the Egyptian sequences.**

We analyzed 155 E2 sequences from outside Egypt in addition to the new sequences in this paper. The region that is common among all of these sequences has been identified and the subsequences including it were extracted. The length of each sequence in the final set was 133 bp. In our sequences in this paper, the common region starts at position 618 and ends at position 740*.* Figure 9 shows the phylogenetic tree for the 155 sequences from different countries (including ours) which cover the E2 region. It can be also observed that sequences from the same region cluster together in separate sub-trees.

### Estimation of clock rates for non-Egyptian sequences

We have estimated the molecular clock and the rate of change using the program BEAST as we explained before. The rate of change for the first set of E1 sequences was 8.48E-3 per site per year, while it was 5.38E-3 for the second set of E1. In fact, the rate of change for the first part is not significant due to the small number of the sequences and their short lengths. For E2, it was estimated to be 6.3E-3 per site per year.

## Discussion

Egypt has the highest endemic transmission and prevalence of HCV in the world [8,27]**,** The high degree of natural genetic variation in HCV poses a significant challenge for antiviral chemotherapy and hinders the construction of effective vaccines against HCV [28]. The genetic divergence within the region of envelope glycoprotein (E1/E2) of HCV is mainly responsible for the variability of HCV genome [29].

Although there is a lot of work for sequencing HCV genomes and analyzing them, very little work has been accomplished to characterize the HCV genome of genotype 4, as can be observed by querying the different biological databases. Currently available data are highly biased towards genotype 1. For example, 70% out of the 143 published distinct full genomes in the LANL HCV database are of genotype 1, and 87% of these are specific to subtype 1b. This emphasizes the substantial lack of full genome sequences available for many of the HCV genotypes and subtypes [15].

In the present study we present a new dataset including sequences covering the whole E1/E2 region of HCV genotype 4a. Specifically, we targeted the full range of 1672 bp of the E1/E2 region. Because the only previous study [30] targeted only 105 bp of the E1/E2 region, the current work is then the first of its type in terms of coverage of the E1/E2 region.

Sequencing the E1/E2 region (HCV nucleotide position 874 to 2551) from Egyptian isolates and sequence analysis ascertains its high variability, even within the same infected individual.

The analysis of variability across the E1/E2 sequence sites is particularly interesting. First, it asserts that E1/E2 is in general a rapidly evolving region of the HCV genome, as previously studied in [31]. Second, it clearly shows high degree of variability and the genetic divergence codons at certain positions (Figures 1–4).

These areas mainly concentrated on the highly variable regions. Interestingly, we could observe variable regions in addition to HVR1 and HVR2. These newly observed ones are in agreement with the previously reported regions in [32] and named HVR495 and HVR575. It is worth mentioning that these two HVR regions were reported in subtypes 4a but were not present in subtypes 1a, 1b, 2a, or 6a [32]. It is important to note that highly variable regions in E1/E2 may reflect the generation of escape mutants as a consequence of the immune response [34]. Such associations have been addressed in [35], where multiple variants established infection in chimeric mice and selective sweep has occurred after transmission of HCV.

Phylogenetic analysis of our new 36 sequences showed that they are grouped into two main distinct groups: one contains sequences (1D, 8E, 6B, 7A, 9D, 9A) which shows noticeable degree of variability and another cluster that contains a clade of sequences (4D, 5D, 1E, 7D, 3D, 10B, 5E, 2D, 2E, 6E) with no significant genetic heterogeneity and high homology among the sequences. Similar study on Phylogenetic tree reconstructions showed two distinct clades existing within the 1a subtype with each clade having a star-like tree topology [33].

Another group that arises into different branched and sub clusters also showed variability among some sequences and homology between the sequences obtained from patient C. In another analyses, we created independent phylogenetic tree to each group of sequences representing the same patient, which revealed a high degree of genetic heterogeneity among the same infected individual with different variants of HCV genotype 4a. An exception for that was group C, which showed less variability.

We collected public sequences from different countries isolated at different time points. We studied their variability and compared them to our new 36 sequences. Our results confirm not only region specific variability but also evolution of the sequences over the period of study. In [39], the authors sequenced 36 samples from 10 HCV patients (Genotype 1a) over a period of 7–21 years. The patients were from the USA, Japan, and Egypt. The sequences included 1,778 nucleotides from the core gene, E1/E2, and the NS5b region. Phylogenetic analysis of this dataset showed different geographical distribution and spread times among the three countries. In this study, the authors also discussed the related socioeconomic, medical and paramedical events that can be responsible for the spread of HCV infection in these countries in the past [36]. Our results in this paper show similar pattern for Genotype 4a, where sequences from the same region and same time point tend to cluster together especially for the Egyptian sequences [37].

We would like also to indicate that our sequences are the most recent ones, which also add temporal dimension to this separation. It is most likely that E1 also evolves at a high rate similar to E2, which hosts the highly variable regions.

It is clear how our Egyptian sequences cluster together in a clear separation from the others. The other big Irish and USA data has also been grouped together. These results suggest a region specific evolution of the virus of this genotype.

Although the observed variability cannot be confirmed as a prognostic tool in assessment of variants of acute hepatitis C course [38], it is likely related to functional and immunological determinants of different HCV neutralizing epitopes [11], viral transmission [30] and development of hepatocellular carcinoma [39]. It is worth mentioning that the present findings are compatible with our previously published data on HCV-4 supporting viral genetic complexity and variability in the 5’UTR region [40] and different quasispecies that may play a role in the response to IFN treatment [41]. Still, further studies using more datasets are needed to confirm E2 variability of HCV-4a and whether it is related to the course of HCV infection or not.

The rate of change of E1 region within the Egyptian sequences from 1997 to 2012 has been estimated to be 5E-3 per site per year, and the rate of E1 for none Egyptian sequences was 5.38E-3 per site per year. For E2, the rate of change was 8E-3 for Egyptian isolated and it was 6E-3 for non-Egyptian isolates. Whether the rate of change differs from one region to another or not remains an open question that needs further validation using more datasets in future.

## Conclusion

Sequencing and phylogenetic analysis of 36 new sequences covering full region of E1/E2 region of HCV 4a were conducted. Moreover, 254 E1 sequences and 8 E2 sequences isolated from Egypt in the period from 1997 to 2010 were collected. Also, 218 E1 sequences and 155 E2 sequences from different countries were collected from public databases. Phylogenetic analysis of all these datasets was conducted to study temporal and region specific evolution of HCV-4a. The estimated rate of change for Egyptian sequences was 5E-3 and 8E-3 per site per year for E1 and E2, respectively. For non-Egyptian sequences, the rates were 5.38E-3 and 6.3E-3 per site per year for E1 and E2, respectively. The results of this study support the high rate of evolution of these regions in genotype 4a. It has also revealed the significant level of variability among sequences from different regions of the world. Whether the clock rates do differ from one region to another need further validation using more datasets in future, especially for the E2 region. Further research is also required to associate the observed variability to functional issues, such as immune escape, virulence, and disease pathogenesis.

## Additional files

Additional file 1:
**Multiple sequence alignment(s) of the sequences.**
Additional file 2:
**Details of collected sequences, including the origin, the date of isolation, average lengths and the respective publications.**
