# Supplementary material for: New insight into HCV E1/E2 region of genotype 4a
Source: Virol J. 2014 Dec 30;11:231. doi: 10.1186/s12985-014-0231-y (PMC4304183; doi:10.1186/s12985-014-0231-y)
Supplement: Additional file 1: — Multiple sequence alignment(s) of the sequences. [file 12985_2014_231_MOESM1_ESM.docx]

**HCV+Egypt+Genotype4a+E1:**

| **Title** | **Authors** | **Sequence Identifiers (GI)** | **#Seq** | **Length** | **Date of collection/ publication** | **NCBI** | **LANL** | **EUHCV** |
| --- | --- | --- | --- | --- | --- | --- | --- | --- |
| The Core/E1 domain of hepatitis C virus genotype 4a in Egypt does not contain viral mutations or strains specific for hepatocellular carcinoma | Zhang,X., Ryu,S.H., Xu,Y., Elbaz,T., Zekri,A.R., Abdelaziz,A.O., Abdel-Hamid,M., Thiers,V., Elena,S.F., Fan,X. and Di Bisceglie,A.M. | 334701163  334701444 (HQ615723-HQ615725) | 146 | 387 | 2003/2011 | 146 | - | - |
| Intrafamilial clustering of subtype 4u, a new Hepatitis C subtype in a rural village of the Nile delta | El-Daly,M., Thiers,V., Rimlinger,F., Rekacewicz,C., Diliberto,G., Fontanet,A., Feray,C. and Abdel Hamid,M. | 157419469  - 157419599  (EFxxxxxx -EF694386) | 39 | 426 | 2002/ 2008 | 39 | - | 39 |
| Molecular epidemiology of hepatitis C virus genotype 4 isolates in Egypt and analysis of the variability of envelope proteins E1 and E2 in patients with chronic hepatitis | Genovese,D., Dettori,S., Argentini,C., Villano,U., Chionne,P., Angelico,M. and Rapicetta,M. | 3980266-3980279  (AJ002751-  AJ002762) | 12 | 227 | 1997/2005 | 12 | - | 12 |
| Genetic epidemiology of hepatitis C virus throughout egypt | Ray,S.C., Arthur,R.R., Carella,A., Bukh,J. and Thomas,D.L. | 10304180  -10304276  (AF271825-  AF271873) | 49 | 411 | 2000/2000 | 49 | - | 49 |
| Novel infectious cDNA clones of hepatitis C virus genotype 3a (strain S52) and 4a (strain ED43): genetic analyses and in vivo pathogenesis studies | Gottwein,J.M., Scheel,T.K., Callendret,B., Li,Y.P., Eccleston,H.B., Engle,R.E., Govindarajan,S., Satterfield,W., Purcell,R.H.,\n Walker,C.M. and Bukh,J. | 295311563-  295311563  **(**GU814265) | 1 | FULL | 2008OR 1997/  2010 | 1 | 1 | - |
| Direct Submission | Bernardin,F., Abdel-Hamid,M., Cooper,S., Wakil,E., Nygaard,K., Ostrowski,M.-J., Mohamed,M.K., Fontanet,A. and Delwart,E.L. | 119331257-  119331269  (DQ988073-DQ988079) | 7 | FULL | 2006/  2007 | 7 | 7 | 7 |
| **TOTAL** |  |  | **254** |  |  | **254** | **8** | **107** |

**HCV+Egypt+Genotype4a+E2:**

| **Title** | **Authors** | **Sequence Identifiers (GI)** | **#Seq** | **Length** | **Date of collection/ publication** | **NCBI** | **LANL** | **EUHCV** |
| --- | --- | --- | --- | --- | --- | --- | --- | --- |
| Novel infectious cDNA clones of hepatitis C virus genotype 3a (strain S52) and 4a (strain ED43): genetic analyses and in vivo pathogenesis studies | Gottwein,J.M., Scheel,T.K., Callendret,B., Li,Y.P., Eccleston,H.B., Engle,R.E., Govindarajan,S., Satterfield,W., Purcell,R.H.,\n Walker,C.M. and Bukh,J. | 295311563  295311563  **Prefix**  GU814265 | 1 | FULL | 2008OR 1997/  2010 | 1 | 1 | - |
| Direct Submission | Bernardin,F., Abdel-Hamid,M., Cooper,S., Wakil,E., Nygaard,K., Ostrowski,M.-J., Mohamed,M.K., Fontanet,A. and Delwart,E.L. | 119331257-  119331269  (DQ988073-DQ988079) | 7 | FULL | 2006/  2007 | 7 | 7 | 7 |
| TOTAL |  |  | 8 |  |  | 8 | 8 | 7 |

**HCV+NotEgypt+Genotype4a+E1:**

| **Title** | **Authors** | **Country** | **Sequence Identifiers (GI)** | **#Seq** | **Length** | **Date of collection/ publication** | **NCBI** | **LANL** | **EUHCV** |
| --- | --- | --- | --- | --- | --- | --- | --- | --- | --- |
| Use of sequence analysis of the NS5B region for routine genotyping of hepatitis C virus with reference to C/E1 and 5' untranslated region sequences | Murphy,D.G., Willems,B., Deschenes,M., Hilzenrat,N., Mousseau,R and Sabbah,S. | Canada | 134037586  134038438  (EF115894-EF116143)  Core/E1 Junction | 14 | 424 (average) | 2006/2006 | 14 | - | 7  (EF115894, EF115897, EF115901, EF115903, EF115907, EF115912, EF115920) |
| Use of sequence analysis of the NS5B region for routine genotyping of hepatitis C virus with reference to C/E1 and 5' untranslated region sequences | Murphy,D.G., Willems,B., Deschenes,M., Hilzenrat,N., Mousseau,R and Sabbah,S. | Canada | 383215728  -383215984  (JQ318256-  JQ318384)  Core/E1 Junction | 2 | 424 (average) | 2010/2010 | 2 | - | - |
| Characterization of full-length hepatitis C virus genotype 4 sequences | Timm, J., Neukamm, M.M., Kuntzen, T., Brander, C., Chung, R.T., Lauer, G.M., Walker, B.D., Allen, T.M. | USA | DQ418782-  [DQ418789](http://hcv.lanl.gov/components/sequence/HCV/asearch/query_one.comp?se_id=13572)   \|  \|  \| \| --- \| --- \| | 6 | Full | 2006 | 6 | 6 | - |
| Identification and characterization of broadly neutralizing human monoclonal antibodies directed against the E2 envelope glycoprotein of hepatitis C virus. | Broering TJ, Garrity KA, Boatright NK, Sloan SE, Sandor F, Thomas WD Jr, Szabo G, Finberg RW, Ambrosino DM, Babcock GJ | USA | 260748846-  260748846  (GQ379230- GQ379230)  E1+E2 | 1 | 1746 | 2007 | 1 | 1 | 1 |
| Insertion and recombination events at hypervariable region 1 over 9.6 years of hepatitis C virus chronic infection | Palmer,B.A., Moreau,I., Levis,J., Harty,C., Crosbie,O., Kenny-Walsh,E. and Fanning,L.J. | Ireland | 261351281  261351359  (FJ744067-FJ744106) | 40 | 320 | 2009 | 40 | - | 40 |
| Insertion and recombination events at hypervariable region 1 over 9.6 years of hepatitis C virus chronic infection | Palmer,B.A., Moreau,I., Levis,J., Harty,C., Crosbie,O., Kenny-Walsh,E. and Fanning,L.J. |  | 307696085-307696183  (GQ985330-GQ985379) | 50 | 320 | 2009 | 50 | - | 50 |
| Insertion and recombination events at hypervariable region 1 over 9.6 years of hepatitis C virus chronic infection | Palmer,B.A., Moreau,I., Levis,J., Harty,C., Crosbie,O., Kenny-Walsh,E. and Fanning,L.J. | Ireland | 334724722  334724766  (HM363383, HM363405) | 23 | 320 | 2008 | 23 | - | - |
| Insertion and recombination events at hypervariable region 1 over 9.6 years of hepatitis C virus chronic infection | Palmer,B.A., Moreau,I., Levis,J., Harty,C., Crosbie,O., Kenny-Walsh,E. and Fanning,L.J. | Ireland | 425893156  425893184  (JQ743303-JQ743317) E1-E2 HVR1 | 15 | 320 | 2009 | 15 | - | - |
| Separation of Hepatitis C genotype 4a into IgG-depleted and IgG-enriched fractions reveals a unique quasispecies profile | Moreau,I., O'Sullivan,H., Murray,C., Levis,J., Crosbie,O., Kenny-Walsh,E. and Fanning,L.J. | Ireland | 186892403  186892441  (EU482129, EU482148)  E1-E2 HVR1 | 20 | 323 | 2008 | 20 | - | 20 |
| Introduction of new subtypes and variants of hepatitis C virus genotype 4 in South Africa. | Gededzha MP, Selabe SG, Kyaw T, Rakgole JN, Blackard JT, Mphahlele MJ. | South Africa | 357535583  (JN116569) C/E1 | 1 | 324 | 2010 | 1 | - | - |
| NA | Seneviranthna, D.B. and Fernandupulle, N.D. | Sri Lanka | 262478627-262478629  (GU075874-  GU075875)  Core/E1 | 2 | 420 | 2009 | 2 | - | - |
| Molecular characterization of genotype 2 and 4 hepatitis C virus isolates in French blood donors. | Cantaloube JF, Gallian P, Laperche S, Elghouzzi MH, Piquet Y, Bouchardeau F, Jordier F, Biagini P, Attoui H, de Micco P. | France | 269131433-269131511  (GU054339- GU054378) | 8 | 276 | 2000 | 8 | - | 1 (GU054339) |
| J Med Virol. 2009 Jul;81(7):1189-97. doi: 10.1002/jmv.21466.  Epidemic spread of hepatitis C virus genotype 3a and relation to high incidence of hepatocellular carcinoma in Pakistan. | Khan A, Tanaka Y, Azam Z, Abbas Z, Kurbanov F, Saleem U, Hamid S, Jafri W, Mizokami M | Pakistan | 225380549  225380551  (AB444558- AB444558) | 2 | 432 | 2008 | 2 | - | 2 |
| Hepatitis C virus subtyping based on sequencing of the C/E1 and NS5B genomic regions in comparison to a commercially available line probe assay | Avo,A.P., Agua-Doce,I., Andrade,A. and Padua,E. | Portugal | 410520251  410520430 (JQ272507, JQ272512- JQ272598) | 19 | 420  C/E1 | 2008 | 19 | - | - |
| Adaptive mutations allow establishment of jfh1-based cell culture systems for hepatitis c virus genotype 4a | Scheel,T.K., Gottwein,J.M., Eugen-Olsen,J. and Bukh,J. | Denmark | 218043714  -218043729  (GM864803- GM864819, JA206597- JA206598) | 16 | Full | 2008 | 16 | - | - |
| Complete nucleotide sequence of genotype 4 hepatitis C viruses isolated from patients co-infected with human immunodeficiency virus type 1 | Franco, S., Tural, C., Clotet, B., Martinez, M.A. | Spain | DQ516084 | 1 | Full | 2006 | 1 | - | 1 |
| Complete nucleotide sequence of a type 4 hepatitis C virus variant,  the predominant genotype in the Middle East | Chamberlain,R.W. | Middle East | GI:157781208 (NC_00982) | 1 | FULL | 1997 | 1 | - | - |
| Complete nucleotide sequence of a type 4 hepatitis C virus variant,  the predominant genotype in the Middle East | Chamberlain,R.W. | Middle East | GI:2252489 (Y11604) | 1 | FULL | 1997 | 1 | - | - |
| **TOTAL** |  |  | **222** |  |  |  | **222** | **7** | **115** |

**HCV+NotEgypt+Genotype4a+E2:**

| **Title** | **Authors** | **Country** | **Files names ranges** | **#seq** | **len** | **Year** | NCBI | LANL | EUHCV |
| --- | --- | --- | --- | --- | --- | --- | --- | --- | --- |
| Analysing the evolutionary history of HCV: puzzle of ancient phylogenetic discordance. | Magiorkinis G, Ntziora F, Paraskevis D, Magiorkinis E, Hatzakis A. | Greece | 396925075  396925091  (JN563670- JN563678)  E2-P7-NS2 | 9 | 639 | 1999 | 9 | 1 | 1 |
| Characterization of full-length hepatitis C virus genotype 4 sequences | Timm, J., Neukamm, M.M., Kuntzen, T., Brander, C., Chung, R.T.,  Lauer, G.M., Walker, B.D., Allen, T.M. | USA | DQ418782-  [DQ418789](http://hcv.lanl.gov/components/sequence/HCV/asearch/query_one.comp?se_id=13572)   \|  \|  \| \| --- \| --- \| | 6 | Full | 2006 | 6 | 6 | - |
| Identification and characterization of broadly neutralizing human monoclonal antibodies directed against the E2 envelope glycoprotein of hepatitis C virus. | Broering TJ, Garrity KA, Boatright NK, Sloan SE, Sandor F, Thomas WD Jr, Szabo G, Finberg RW, Ambrosino DM, Babcock GJ | USA | 260748846-  260748846  (GQ379230- GQ379230)  E1+E2 | 1 | 1746 | 2007 | 1 | 1 | 1 |
| Insertion and recombination events at hypervariable region 1 over 9.6 years of hepatitis C virus chronic infection | Palmer,B.A., Moreau,I., Levis,J., Harty,C., Crosbie,O., Kenny-Walsh,E. and Fanning,L.J. | Ireland | 261351281  261351359  (FJ744067-FJ744106) | 40 | 320 | 2009 | 40 | - | 40 |
| Insertion and recombination events at hypervariable region 1 over 9.6 years of hepatitis C virus chronic infection | Palmer,B.A., Moreau,I., Levis,J., Harty,C., Crosbie,O., Kenny-Walsh,E. and Fanning,L.J. |  | 307696085-307696183  (GQ985330-GQ985379) | 50 | 320 | 2009 | 40 | - | 40 |
| Insertion and recombination events at hypervariable region 1 over 9.6 years of hepatitis C virus chronic infection | Palmer,B.A., Moreau,I., Levis,J., Harty,C., Crosbie,O., Kenny-Walsh,E. and Fanning,L.J. | Ireland | 334724722  334724766  (HM363383, HM363405) | 23 | 320 | 2008 | 23 | - | - |
| Insertion and recombination events at hypervariable region 1 over 9.6 years of hepatitis C virus chronic infection | Palmer,B.A., Moreau,I., Levis,J., Harty,C., Crosbie,O., Kenny-Walsh,E. and Fanning,L.J. | Ireland | 425893156  425893184  (JQ743303-JQ743317) E1-E2 HVR1 | 15 | 320 | 2009 | 15 | - | - |
| Separation of Hepatitis C genotype 4a into IgG-depleted and IgG-enriched fractions reveals a unique quasispecies profile | Moreau,I., O'Sullivan,H., Murray,C., Levis,J., Crosbie,O., Kenny-Walsh,E. and Fanning,L.J. | Ireland | 186892403  186892441  (EU482129, EU482148)  E1-E2 HVR1 | 20 | 323 | 2008 | 20 | - | 20 |
| Adaptive mutations allow establishment of jfh1-based cell culture systems for hepatitis c virus genotype 4a | Scheel,T.K., Gottwein,J.M., Eugen-Olsen,J. and Bukh,J. | Denmark | 218043714  -218043729  (GM864803- GM864819, JA206597- JA206598) | 16 | Full | 2008 | 16 | - | - |
| Complete nucleotide sequence of genotype 4 hepatitis C viruses isolated from patients co-infected with human immunodeficiency  virus type 1 | Franco, S., Tural, C., Clotet, B., Martinez, M.A. | Spain | DQ516084 | 1 | Full | 2006 | 1 | - | 1 |
| Complete nucleotide sequence of a type 4 hepatitis C virus variant,  the predominant genotype in the Middle East | Chamberlain,R.W. | Middle East | GI:157781208 (NC_00982) | 1 | FULL | 1997 | 1 | - | - |
| Complete nucleotide sequence of a type 4 hepatitis C virus variant,  the predominant genotype in the Middle East | Chamberlain,R.W. | Middle East | GI:2252489 (Y11604) | 1 | FULL | 1997 | 1 | - | - |
| **TOTAL** |  |  |  | **183** |  |  | **183** | **8** | **103** |
