# Supplementary material for: New insight into HCV E1/E2 region of genotype 4a
Source: Virol J. 2014 Dec 30;11:231. doi: 10.1186/s12985-014-0231-y (PMC4304183; doi:10.1186/s12985-014-0231-y)
Supplement: Additional file 2: — Details of collected sequences, including the origin, the date of isolation, average lengths and the respective publications. [file 12985_2014_231_MOESM2_ESM.pdf]

```
CLUSTAL multiple sequence alignment by MUSCLE (3.8)


7A              CTTGGCCCTCTCTCGTGCTGACTGTCCCCGCCCTCGGCCATCAACTATCACAATACTTCG
6B              CCTGGCCCTCTCTCGTGCTGACTGTCCCCGCCTTCGGCCATCAACTATCACAATACTTCG
9D              TCTGGCCCTCTCTCGTGCTGACTGTCCCCGCCTTCGGCCATCAACTACCACAATACTTCG
1D              CTTGGCCCTCTCTCGTGCTGACTGTCCCCGCCTTCGGCCATCAACTATCACAATACTTCG
8E              CCTGGCTCTCTCGTGCCCTGACTGTCCCCGCCTTCGGCCATCAACTATCACAATACTTCG
9A              TTTGGGCTTCTCTCGTGCTGACTGTCCCCGCCTTCGGCCATCAACTATCACAATACTTCG
10D             TCTTGGCCTCTCTCGTGCTGACTGTCCCCGCCTTCGGCCATCAACTATCACAATACTTCA
3D              TTCTGGCTTCTCTCGTGCTGACTGTCCCCGCCTTCGGCCATCAACTATCACAATACTTCA
4D              TCTTGGCCTCTCTCGTGCTGACTGTCCCCGCCTTCGGCCATCAACTATCACAATACTTCA
5D              TCTTGCCCTCTCTCGTGCTGACTGTCCCCGCCTTCGGCCATCAACTATCACAATACTTCA
10B             CTTGGCCCTCTCTCGTGCTGACTGTCCCCGCCTTCGGCCATCAACTATCACAATACTTCA
5E              TCTTGGCCTCTCTCGTGCTGACTGTCCCCGCCTTCGGCCATCAACTATCACAATACTTCA
1E              TCTTGGCCTCTCTCGTGCTGACTGTCCCCGCCTTCGGCCATCAACTATCACAATACTTCA
7D              TCTTGGCCTCTCTCGTGCTGACTGTCCCCGCCTTCGGCCATCAACTATCACAATACTTCA
2D              CCTTGGCCTCTCTCGTGCTGACTGTCCCCGCCTTCGGCCATCAACTATCACAATACTTCA
2E              TCTTGGCCTCTCTCGTGCTGACTGTCCCCGCCTTCGGCCATCAACTATCACAATACTTCA
6E              TCTTGGCCTCTCTCGTGCTGACTGTCCCCGCCTTCGGCCATCAACTATCACAATACTTCA
2B              TCCTGGCCTCTCTCGTGCTGACTGTCCCCGCCTTCGGCCATCAACTATCACAATACTTCG
3B              CTTGGCCCTCTCTCGTGCTGACTGCCCCCGCCCTCGGCCATCAACTATCACAATACTTCG
6D              CTTGGCCCTCTCTCGTGCTGACTGCCCCCGCCCTCGGCCATCAACTATCACAATACTTCG
9C              TTTTTGGCTCTCTCGTGCTGACTGTCCCCCCCCTCGGCCATCATCTATCACAATACTTCG
2C              CTCTTGCCTCTCTCGTGCTGACTGTCCCCGCCCTCGGCCATCAACTATCACAATACTTCG
5A              TTCTTGGCTCTCTCGTGCTGACTGTCCCCGCCCTCGGCCATCAACTATCACAATACTTCG
8A              CTTGGCCCTCTCTCGTGCTGACTGTCCCCGCCCTCGGCCATCAACTATCACAATACTTCG
3E              TTCTTGGCTCTCTCGTGCTGACTGTCCCCGCCTTCGGCCATCAACTATCACAATACTTCG
4E              TCCTTGGCTCTCTCGTGCTGACTGTCCCCGCCCTCGGCCATCAACTATCACAATACTTCG
10A             TCTTGGACTCTCTCGTGCTGACTGTCCCCGCCCTCGGCCATCAACTATCACAATACTTCG
7B              TCTTGGCCTCTCTCGTGCTGACTGTCCCCGCCCTCGGCCATCAACTATCACAATACTTCG
6C              TCTTGGCCTCTCTCGTGCTGACTGTCCCCGCCCTCGGCCATCATCTATCACAATACTTCG
8C              TCTCTGGCTCTCTCGTGCTGACTGTCCCCCGCCTCGGCCATCATCTATCACAATACTTCG
1C              TTCTTGGCTCTCTCGTGCTGACTGTCCCCGCCCTCGGCCATCATCTATCACAATACTTCG
4C              TTTCTGGCTCTCTCGTGCTGACTGTCCCCGCCCTCGGCCATCATCTATCACAATACTTCG
7C              TTCCTGCCTCTCTCGTGCTGACTGTCCCCGCCCTCGGCCATCATCTATCACAATACTTCG
10C             TTCTTGCCTCTCTCGTGCTGACTGTCCCCGCCCTCGGCCATCATCTATCACAATACTTCG
5C              TTCTTGGCTCTCTCGTGCTGACTGTCCCCGCCCTCGGCCATCATCTATCACAATACTTCG
3C              TTCTTGGCTCTCTCGTGCTGACTGTCCCCGCCCTCGGCCATCATCTATCACAATACTTCG
                        ****  *  ******* ****  * ********** *** *********** 

7A              GGCATCTACCATGTCACCAACGACTGCCCTAATTCAAGTATAGTATATGAGGCCGACCAT
6B              GGCATCTACCATGTCACCAATGACTGCCCTAACTCAAGTATAGTATATGAGGCCGACCAT
9D              GGCATCTACCATGTCACCAACGACTGCCCTAACTCAAGTATAGTATATGAGGCCGACCAT
1D              GGCATCTACCACCCTTCCAACGACTGCCCTAACTCAAGTATAGTATATGAGGCCGACCAT
8E              GGCATCTACCATGTCACCAACGACTGCCCTAACTCAAGTATAGTATATGAGGCCGACCAT
9A              GGCATCTACCATGTCACCAACGACTGCCCTAATTCAAGTATAGTATATGAGGCCGACCAC
10D             GGCATCTACCATGTCACCAACGACTGCCCTAATTCAAGTATAGTATATGAGGCCGACCAT
3D              GGCATCTACCATGTCACCAACGACTGCCCTAATTCAAGTATAGTATATGAGGCCGACCAT
4D              GGCATCTACCATGTCACCAACGACTGCCCTAATTCAAGTATAGTATATGAGGCCGACCAT
5D              GGCATCTACCATGTCACCAACGACTGCCCTAATTCAAGTATAGTATATGAGGCCGACCAT
10B             GGCATCTACCATGTCACCAACGACTGCCCTAATTCAAGTATAGTATATGAGGCCGACCAT
5E              GGCATCTACCATGTAACCAACGACTGCCCTAATTCAAGTATAGTATATGAGGCCGACCAT
1E              GGCATCTACCATGTCACCAACGACTGCCCTAATTCAAGTATAGTATATGAGGCCGACCAT
7D              GGCATCTACCATGTCACCAACGACTGCCCTAATTCAAGTATAGTATATGAGGCCGACCAT
2D              GGCATCTACCATGTCACCAACGACTGCCCTAATTCAAGTATAGTATATGAGGCCGACCAT
```

```
2E              GGTATCTACCATGTCACCAACGACTGCCCTAATTCAAGTATAGTATATGAGGCCGACCAT
6E              GGCATCTACCATGTCTCCAACGACTGCCCTAATTCAAGTATAGTATATGAGGCCGACCAT
2B              GGCATCTACCATGTCACCAACGACTGCCCTAACTCAAGTATAGTATATGAGGCCGACCAT
3B              GGCATCTACCCCCTCTACAAAGACTGCCCTAATTCAAGTATAGTATATGAGGCCGACCAT
6D              GGCATCTACCCCCTCTCCAACGACTGCCCTAATTCAAGTATAATATATGAGGCCGACCAT
9C              GGCATCTACCATGTCACCAACGACTGCCCTAATTCAAGTATAGTATATGAGGCCGACCAT
2C              GGCATCTACCCCCTCTCCAACGACTGCCCTAATTCAAGTATAGTATATGAGGCCGACCAT
5A              GGCATCTACCATGTCACCAACGACTGCCCTAATTCAAGTATAGTATATGAGGCCGACCAT
8A              GGCATCTACCATGTCACCAACGACTGCCCTAATTCAAGTATAGTATATGAGGCCGACCAT
3E              GGCATCTACCATGTCACCAACGACTGCCCTAATTCAAGTATAGTATATGAGGCCGACCAT
4E              GGCATCTACCATGTCACCAACGACTGCCCTAATTCAAATATAGTATATGAGGCCGACCAT
10A             GGCATCTACCATGTCACCAACGACTGCCCTAATTCAAGTATAGTATATGAGGCCGACCAT
7B              GGCATCTACCATGTCACCAACGACTGCCCTAATTCAAGTATAGTATATGAGGCCGACCAT
6C              GGCATCTACCATGTCACCAACGACTGCCCTAATTCAAGTATAGTATATGAGGCCGACCAT
8C              GGCATCTACCATGTCACCAACGACTGCCCTAATTCAAGTATAGTATATGAGGCCGACCAT
1C              GGCATCTACCATGTCACCAACGACTGCCCTAATTCAAGTATAGTATATGAGGCCGACCAT
4C              GGCATCTACCATGTCACCAACGACTGCCCTAATTCAAGTATAGTATATGAGGCCGACCAT
7C              GGCATCTACCATGTCACCAACGACTGCCCTAATTCAAGTATAGTATATGAGGCCGACCAT
10C             GGCATCTACCATGTCACCAACGACTGCCCTAATTCAAGTATAGTATATGAGGCCGACCAT
5C              GGCATCTACCATGTCACCAACGACTGCCCTAATTCAAGTATAGTATATGAGGCCGACCAC
3C              GGCATCTACCATGTCACCAACGACTGCCCTAATTCAAGTATAGTATATGAGGCCGACCAT
                ** *******       *** *********** **** **** ****************

7A              CACATCCTGCACCTTCCAGGTTGCGTGCCCTGTGTGAGAAGCGGAAATCAGTCACATTGC
6B              CACATCTTGCACCTTCCAGGTTGCGTGCCCTGTGTGAGAAGCGGAAATCAGTCACATTGC
9D              CACATCTTGCACCTTCCAGGTTGCGTGCCCTGTGTGAGAAGCGGAAATCAGTCACATTGC
1D              CACATCTTGCACCTCCCAGGTTGCGTGCCCTGTGTGAGAAGCGGAAATCAGTCACATTGC
8E              CACATCTTGCACCTCCCAGGTTGCGTGCCCTGTGTGAGAAGCGGAAATCAGTCACATTGC
9A              CACATCCTGCACCTCCCAGGTTGCGTGCCCTGTGTGAGAAGCGGAAATCAGTCACATTGC
10D             CACATCCTGCACCTCCCAGGTTGCGTGCCCTGTGTGAGAAGCGGAAATCAGTCACATTGC
3D              CACATCCTGCACCTCCCAGGTTGCGTGCCCTGTGTGAGAAGCGGAAATCAGTCACATTGC
4D              CACATCCTGCACCTCCCAGGTTGCGTGCCCTGTGTGAGAAGCGGAAATCAGTCACATTGC
5D              CACATCCTGCACCTCCCAGGTTGCGTGCCCTGTGTGAGAAGCGGAAATCAGTCACATTGC
10B             CACATCCTGCACCTCCCAGGTTGCGTGCCCTGTGTGAGAAGCGGAAATCAGTCACATTGC
5E              CACATCCTGCACCTCCCAGGTTGCGTGCCCTGTGTGAGAAGCGGAAATCAGTCACATTGC
1E              CACATCCTGCACCTCCCAGGTTGCGTGCCCTGTGTGAGAAGCGGAAATCAGTCACATTGC
7D              CACATCCTGCACCTCCCAGGTTGCGTGCCCTGTGTGAGAAGCGGAAATCAGTCACATTGC
2D              CACATCCTGCACCTCCCAGGTTGCGTGCCCTGTGTGAGAAGCGGAAATCAGTCACATTGC
2E              CACATCCTGCACCTCCCAGGTTGCGTGCCCTGTGTGAGAAGCGGAAATCAGTCACATTGC
6E              CACATCCTGCACCTCCCAGGTTGCGTGCCCTGTGTGAGAAGCGGAAATCAGTCACATTGC
2B              CACATCTTGCGCCTCCCAGGTTGCGTGCCCTGTGTGAGAAGCGGAAATCAGTCACATTGC
3B              CACATCCTGCACCTTCCAGGTTGCGTGCCCTGTGTGAGAAGCGGAAATCAGTCACATTGC
6D              CACATCCTGCACCTTCCAGGTTGCGTGTCCTGTGTGAGAAGCGGAAATCAGTCACATTGC
9C              CACATCCTGCACCTTCCAGGTTGCGTGCCCTGTGTGAGAAGCGGAAATCAGTCACATTGC
2C              CACATCCTGCACCTTCCAGGTTGCGTGCCCTGTGTGAGAAGCGGAAATCAGTCACATTGC
5A              CACATCCTGCACCTTCCAGGTTGCGTGTCCTGTGTGAGAAGCGGAAATCAGTCACATTGC
8A              CACATCCTGCACCTTCCAGGTTGCGTGCCCTGTGTGAGAAGCGGAAATCAGTCACATTGC
3E              CACATCCTGCACCTTCCAGGTTGCGTGTCCTGTGTGAGAAGCGGAAATCAGTCACATTGC
4E              CACATCCTGCACCTTCCAGGTTGCGTGTCCTGTGTGAGAAGCGGAAATCAGTCACATTGC
10A             CACATCCTGCACCTTCCAGGTTGCGTGCCCTGTGTGAGAAGCGGAAATCAGTCACATTGC
7B              CACATCCTGCACCTTCCAGGTTGCGTGCCCTGTGTGAGAAGCGGAAATCAGTCACATTGC
6C              CACATCCTGCACCTTCCAGGTTGCGTGCCCTGTGTGAGAAGCGGAAATCAGTCACATTGC
8C              CACATCCTGCACCTTCCAGGTTGCGTGCCCTGTGTGAGAAGCGGAAATCAGTCACATTGC
1C              CACATCCTGCACCTTCCAGGTTGCGTGCCCTGTGTGAGAAGCGGAAATCAGTCACATTGC
4C              CACATCCTGCACCTTCCAGGTTGCGTGCCCTGTGTGAGAAGCGGAAATCAGTCACATTGC
7C              CACATCCTGCACCTTCCAGGTTGCGTGCCCTGTGTGAGAAGCGGAAATCAGTCACATTGC
10C             CACATCCTGCACCTTCCAGGTTGCGTGCCCTGTGTGAGAAGCGGAAATCAGTCACATTGC
```

```
5C              CACATCCTGCACCTTCCAGGTTGCGTGCCCTGTGTGAGAAGCGGAAATCAGTCACATTGC
3C              CACATCCTGCACCTTCCAGGTTGCGTGCCCTGTGTGAGAAGCGGAAATCAGTCACATTGC
                ****** *** *** ************ ********************************

7A              TGGGTGGCCCTTACTCCTACCGTCGCAGCGCCATACATCGGTGCTCCACTTGAGTCCCTG
6B              TGGGTGGCCCTCACTCCTACCGTCGCAGCGCCGTACATCGGCGCTCCACTTGAGTCCCTG
9D              TGGGTGGCCCTTACTCCTACCGTCGCAGCGCCGTACATCGGCGCTCCACTTGAGTCCCTG
1D              TGGGTGGCCCTTACTCCTACCGTCGCAGCGCCGTACATCGGCGCTCCACTTGAGTCCCTG
8E              TGGGTGGCCCTTACTCCTACCGTCGCAGCGCCGTACATCGGCGCTCCACTTGAGTCCCTG
9A              TGGGTGGCCCTTACTCCTACCGTCGCAGCGCCATACATCGGCGCTCCACTTGAGTCCCTG
10D             TGGGTGGCCCTCACTCCTACCGTCGCAGCGCCATACATCGGCGCTCCACTTGAGTCCCTG
3D              TGGGTGGCCCTCACTCCTACCGCCGCAGCGCCATACATCGGCGCTCCACTTGAGTCCCTG
4D              TGGGTGGCCCTCACTCCTACCGCCGCAGCGCCATACATCGGCGCTCCACTTGAGTCCCTG
5D              TGGGTGGCCCTCACTCCTACCGCCGCAGCGCCATACATCGGCGCTCCACTTGAGTCCCTG
10B             TGGGTGGCCCTCACTCCTACCGCCGCAGCGCCATACATCGGCGCTCCACTTGAGTCCCTG
5E              TGGGTGGCCCTCACTCCTACCGCCGCAGCGCCATACATCGGCGCTCCACTTGAGTCCCTG
1E              TGGGTGGCCCTCACTCCTACCGCCGCAGCGCCATACATCGGCGCTCCACTTGAGTCCCTG
7D              TGGGTGGCCCTCACTCCTACCGCCGCAGCGCCATACATCGGCGCTCCACTTGAGTCCCTG
2D              TGGGTGGCCCTCACTCCTACCGCCGCAGCGCCATACATCGGCGCTCCACTTGAGTCCCTG
2E              TGGGTGGCCCTCACTCCTACCGCCGCAGCGCCATACATCGGCGCTCCACTTGAGTCCCTG
6E              TGGGTGGCCCTCACTCCTACCGCCGCAGCGCCATACATCGGCGCTCCACTTGAGTCCCTG
2B              TGGGTGGCCCTTACTCCTACCGTCGCAGCGCCATACATCGGTGCTCCACTTGAGTCCCTG
3B              TGGGTGGCCCTTACTCCTACCGTCGCAGCGCCATACATCGGTGCTCCACTTGAGTCCCTG
6D              TGGGTGGCCCTTACTCCTACCGTCGCAGCGCCATACATCGGTGCTCCACTTGAGTCCCTG
9C              TGGGTGGCCCTTACTCCTACCGTCGCAGCGCCATACATCGGTGCTCCACTTGAGTCCCTG
2C              TGGGTGGCCCTTACTCCTACCGTCGCAGCGCCATACATCGGTGCTCCACTTGAGTCCCTG
5A              TGGGTGGCCCTTACTCCTACCGTCGCAGCGCCATACATCGGTGCTCCACTTGAGTCCCTG
8A              TGGGTGGCCCTTACTCCTACCGTCGCAGCGCCATACATCGGTGCTCCACTTGAGTCCCTG
3E              TGGGTGGCCCTTACTCCTACCGTCGCAGCGCCATACATCGGTGCTCCACTTGAGTCCCTG
4E              TGGGTGGCCCTTACTCCTACCGTCGCAGCGCCATACATCGGTGCTCCACTTGAGTCCCTG
10A             TGGGTGGCCCTTACTCCTACCGTCGCAGCGCCATACATCGGTGCTCCACTTGAGTCCCTG
7B              TGGGTGGCCCTTACTCCTACCGTCGCAGCGCCATACATCGGTGCTCCACTTGAGTCCCTG
6C              TGGGTGGCCCTTACTCCTACCGTCGCAGCGCCATACATCGGTGCTCCACTTGAGTCCCTG
8C              TGGGTGGCCCTTACTCCTACCGTCGCAGCGCCATACATCGGTGCTCCACTTGAGTCCCTG
1C              TGGGTGGCCCTTACTCCTACCGTCGCAGCGCCATACATCGGTGCTCCACTTGAGTCCCTG
4C              TGGGTGGCCCTTACTCCTACCGTCGCAGCGCCATACATCGGTGCTCCACTTGAGTCCCTG
7C              TGGGTGGCCCTTACTCCTACCGTCGCAGCGCCATACATCGGTGCTCCACTTGAGTCCCTG
10C             TGGGTGGCCCTTACTCCTACCGTCGCAGCGCCATACATCGGTGCTCCACTTGAGTCCCTG
5C              TGGGTGGCCCTTACTCCTACCGTCGCAGCGCCATACATCGGTGCTCCACTTGAGTCCCTG
3C              TGGGTGGCCCTTACTCCTACCGTCGCAGCGCCATACATCGGTGCTCCACTTGAGTCCCTG
                *********** ********** ********* ******** ******************

7A              CGGAGTCATGTGGATCTGATGGTCGGGGCTGCCACTGCTTGTTCAGCCCTTTACATTGGG
6B              CGGAGTCATGTGGATCTGATGGTCGGGGCTGCCACTGCTTGTTCAGCCCTTTACATTGGG
9D              CGGAGTCATGTGGATCTGATGGTCGGGGCTGCCACTGCTTGTTCAGCCCTTTACATTGGG
1D              CGGAGTCATGTGGATCTGATGGTCGGGGCTGCCACTGCTTGTTCAGCCCTTTACATTGGG
8E              CGGAGTCATGTGGATCTGATGGTCGGGGCTGCCACTGCTTGTTCAGCCCTTTACATTGGG
9A              CGGAGTCATGTGGATCTGATGGTCGGGGCTGCCACTGCTTGTTCAGCCCTTTACATTGGG
10D             CGGAGTCATGTGGATCTGATGGTCGGGGCTGCCACTGCTTGTTCAGCCCTTTACATTGGG
3D              CGGAGTCATGTGGATCTGATGGTCGGGGCTGCCACTGCTTGTTCAGCCCTTTACATTGGG
4D              CGGAGTCATGTGGATCTGATGGTCGGGGCTGCCACTGCTTGTTCAGCCCTTTACATTGGG
5D              CGGAGTCATGTGGATCTGATGGTCGGGGCTGCCACTGCTTGTTCAGCCCTTTACATTGGG
10B             CGGAGTCATGTGGATCTGATGGTCGGGGCTGCCACTGCTTGTTCAGCCCTTTACATTGGG
5E              CGGAGTCATGTGGATCTGATGGTCGGGGCTGCCACTGCTTGTTCAGCCCTTTACATTGGG
1E              CGGAGTCATGTGGATCTGATGGTCGGGGCTGCCACTGCTTGTTCAGCCCTTTACATTGGG
7D              CGGAGTCATGTGGATCTGATGGTCGGGGCTGCCACTGCTTGTTCAGCCCTTTACATTGGG
2D              CGGAGTCATGTGGATCTGATGGTCGGGGCTGCCACTGCTTGTTCAGCCCTTTACATTGGG
```

```
2E              CGGAGTCATGTGGATCTGATGGTCGGGGCTGCCACTGCTTGTTCAGCCCTTTACATTGGG
6E              CGGAGTCATGTGGATCTGATGGTCGGGGCTGCCACTGCTTGTTCAGCCCTTTACATTGGG
2B              CGGAGTCATGTGGATCTGATGGTCGGGGCTGCCACTGCTTGTTCAGCCCTTTACATTGGG
3B              CGGAGTCATGTGGATCTGATGGTCGGGGCTGCCACTGCTTGTTCAGCCCTTTACATTGGG
6D              CGGAGTCATGTGGATCTGATGGTCGGGGCTGCCACTGCTTGTTCAGCCCTTTACATTGGG
9C              CGGAGTCATGTGGATTTGATGGTCGGGGCTGCCACTGCTTGTTCAGCCCTTTACATTGGG
2C              CGGAGTCATGTGGATCTGATGGTCGGGGCTGCCACTGCTTGTTCAGCCCTTTACATTGGG
5A              CGGAGTCATGTGGATCTGATGGTCGGGGCTGCCACTGCTTGTTCGGCCCTTTACATTGGG
8A              CGGAGTCATGTGGATCTGATGGTCGGGGCTGCCACTGCTTGTTCAGCCCTTTACATTGGG
3E              CGGAGTCATGTGGATCTGATGGTCGGGGCTGCCACTGCTTGTTCAGCCCTTTACATTGGG
4E              CGGAGTCATGTGGATCTGATGGTCGGGGCTGCCACTGCTTGTTCAGCCCTTTACATTGGG
10A             CGGAGTCATGTGGATCTGATGGTCGGGGCTGCCACTGCTTGTTCAGCCCTTTACATTGGG
7B              CGGAGTCATGTGGATCTGATGGTCGGGGCTGCCACTGCTTGTTCAGCCCTTTACATTGGG
6C              CGGAGTCATGTGGATCTGATGGTCGGGGCTGCCACTGCTTGTTCAGCCCTTTACATTGGG
8C              CGGAGTCATGTGGATCTGATGGTCGGGGCTGCCACTGCTTGTTCAGCCCTTTACATTGGG
1C              CGGAGTCATGTGGATCTGATGGTCGGGGCTGCCACTGCTTGTTCAGCCCTTTACATTGGG
4C              CGGAGTCATGTGGATCTGATGGTCGGGGCTGCCACTGCTTGTTCAGCCCTTTACATTGGG
7C              CGGAGTCATGTGGATCTGATGGTCGGGGCTGCCACTGCTTGTTCAGCCCTTTACATTGGG
10C             CGGAGTCATGTGGATCTGATGGTCGGGGCTGCCACTGCTTGTTCAGCCCTTTACATTGGG
5C              CGGAGTCATGTGGATCTGATGGTCGGGGCTGCCACTGCTTGTTCAGCCCTTTACATTGGG
3C              CGGAGTCATGTGGATCTGATGGTCGGGGCTGCCACTGCTTGTTCAGCCCTTTACATTGGG
                *************** ***************************** **************

7A              GATCTATGTGGCAGCTTGTTCTTGGTTGGTCAGATGTTCTCTTTCCGACCACGGCGCCAC
6B              GATCTATGTGGCGGTTTGTTCTTGGTTGGTCAGATGTTCTCTTTCCGACCACGGCGCCAC
9D              GATCTATGTGGCGGTTTGTTCTTGGTTGGTCAGATGTTCTCTTTCCGACCACGGCGCCAC
1D              GATCTATGTGGCGGTTTGTTCTTGGTTGGTCAGATGTTCTCTTTCCGACCACGGCGCCAC
8E              GATCTATGTGGCGGTTTGTTCTTGGTTGGTCAGATGTTCTCTTTCCGACCACGGCGCCAC
9A              GATCTATGTGGCGGTTTGTTCTTGGTTGGTCAGATGTTCTCTTTCCGACCACGGCGCCAC
10D             GATCTATGTGGCGGTTTGTTCTTGGTTGGTCAGATGTTCTCTTTCCGACCACGGCGCCAC
3D              GATCTATGTGGCGGTTTGTTCTTGGTTGGTCAGATGTTCTCTTTCCGACCACGGCGCCAC
4D              GATCTATGTGGCGGTTTGTTCTTGGTTGGTCAGATGTTCTCTTTCCGACCACGGCGCCAC
5D              GATCTATGTGGCGGTTTGTTCTTGGTTGGTCAGATGTTCTCTTTCCGACCACGGCGCCAC
10B             GATCTATGTGGCGGTTTGTTCTTGGTTGGTCAGATGTTCTCTTTCCGACCACGGCGCCAC
5E              GATCTATGTGGCGGTTTGTTCTTGGTTGGTCAGATGTTCTCTTTCCGACCACGGCGCCAC
1E              GATCTATGTGGCGGTTTGTTCTTGGTTGGTCAGATGTTCTCTTTCCGACCACGGCGCCAC
7D              GATCTATGTGGCGGTTTGTTCTTGGTTGGTCAGATGTTCTCTTTCCGACCACGGCGCCAC
2D              GATCTATGTGGCGGTTTGTTCTTGGTTGGTCAGATGTTCTCTTTCCGACCACGGCGCCAC
2E              GATCTATGTGGCGGTTTGTTCTTGGTTGGTCAGATGTTCTCTTTCCGACCACGGCGCCAC
6E              GATCTATGTGGCGGTTTGTTCTTGGTTGGTCAGATGTTCTCTTTCCGACCACGGCGCCAC
2B              GATTTATGTGGCGGCTTGTTCTTGGTTGGTCAGATGTTCTCTTTCCGACCACGGCGCCAC
3B              GATCTATGTGGCGGCTTGTTCTTGGTTGGTCAGATGTTCTCTTTCCGACCACGGCGCCAC
6D              GATCTATGTGGCAGCTTGTTCTTGGTTGGTCAGATGTTCTCTTTCCGACCACGGCGCCAC
9C              GATTTATGTGGCAGCTTGTTCTTGGTTGGTCAGATGTTCTCTTTCCGACCACGGCGCCAC
2C              GATCTATGTGGCAGCTTGTTCTTGGTTGGTCAGATGTTCTCTTTCCGACCACGGCGCCAC
5A              GATCTATGTGGCGGCTTGTTCTTGGTTGGTCAGATGTTCTCTTTCCGACCACGGCGCCAC
8A              GATCTATGTGGCAGCTTGTTCTTGGTTGGTCAGATGTTCTCTTTCCGACCACGGCGCCAC
3E              GATCTATGTGGCAGCTTGTTCTTGGTTGGTCAGATGTTCTCTTTCCGACCACGGCGCCAC
4E              GATCTATGTGGCAGCTTGTTCTTGGTTGGTCAGATGTTCTCTTTCCGACCACGGCGCCAC
10A             GATCTATGTGGCAGCTTGTTCTTGGTTGGTCAGATGTTCTCTTTCCGACCACGGCGCCAC
7B              GATCTATGTGGCAGCTTGTTCTTGGTTGGTCAGATGTTCTCTTTCCGACCACGGCGCCAC
6C              GATCTATGTGGCAGCTTGTTCTTGGTTGGTCAGATGTTCTCTTTCCGACCACGGCGCCAC
8C              GATCTATGTGGCAGCTTGTTCTTGGTTGGTCAGATGTTCTCTTTCCGACCACGGCGCCAC
1C              GATCTATGTGGCAGCTTGTTCTTGGTTGGTCAGATGTTCTCTTTCCGACCACGGCGCCAC
4C              GATCTATGTGGCAGCTTGTTCTTGGTTGGTCAGATGTTCTCTTTCCGACCACGGCGCCAC
7C              GATCTATGTGGCAGCTTGTTCTTGGTTGGTCAGATGTTCTCTTTCCGACCACGGCGCCAC
10C             GATCTATGTGGCAGCTTGTTCTTGGTTGGTCAGATGTTCTCTTTCCGACCACGGCGCCAC
```

```
5C              GATCTATGTGGCAGCTTGTTCTTGGTTGGTCAGATGTTCTCTTTCCGACCACGGCGCCAC
3C              GATCTATGTGGCAGCTTGTTCTTGGTTGGTCAGATGTTCTCTTTCCGACCACGGCGCCAC
                *** ********* * ********************************************

7A              TGGACTACCCAGGATTGCAATTGCTCCATCTATACGGGTCATATCACTGGCCATAGAATG
6B              TGGACTACCCAGGATTGCAATTGTTCCATCTATACGGGTCATATCACTGGCCACAGAATG
9D              TGGACTACCCAGGATTGCAATTGTTCCATCTATACGGGTCATATCACTGGCCACAGAATG
1D              TGGACTACCCAGGATTGCAATTGTTCCATCTATACGGGTCATATCACTGGCCACAGAATG
8E              TGGACTACCCAGGATTGCAATTGTTCCATCTATACGGGTCATATCACTGGCCACAGAATG
9A              TGGACTACCCAGGACTGCAATTGTTCCATCTATACGGGTCATATCACTGGCCACAGAATG
10D             TGGACTACCCAGGATTGCAATTGTTCCATCTATACGGGTCATATCACTGGCCACAGAATG
3D              TGGACTACCCAGGATTGCAATTGTTCCATCTATACGGGTCATATCACTGGCCACAGAATG
4D              TGGACTACCCAGGATTGCAATTGTTCCATCTATACGGGTCATATCACTGGCCACAGAATG
5D              TGGACTACCCAGGATTGCAATTGTTCCATCTATACGGGTCATATCACTGGCCACAGAATG
10B             TGGACTACCCAGGATTGCAATTGTTCCATCTATACGGGTCATATCACTGGCCACAGAATG
5E              TGGACTACCCAGGATTGCAATTGTTCCATCTATACGGGTCATATCACTGGCCACAGAATG
1E              TGGACTACCCAGGATTGCAATTGTTCCATCTATACGGGTCATATCACTGGCCACAGAATG
7D              TGGACTACCCAGGATTGCAATTGTTCCATCTATACGGGTCATATCACTGGCCACAGAATG
2D              TGGACTACCCAGGATTGCAATTGTTCCATCTATACGGGTCATATCACTGGCCACAGAATG
2E              TGGACTACCCAGGATTGCAATTGTTCCATCTATACGGGTCATATCACTGGCCACAGAATG
6E              TGGACTACCCAGGATTGCAATTGTTCCATCTATACGGGTCATATCACTGGCCACAGAATG
2B              TGGACTACCCAGGATTGCAATTGCTCCATCTATACGGGTCATATCACTGGCCACAGAATG
3B              TGGACTACCCAGGATTGCAATTGCTCCATCTATACGGGTCATATCACTGGCCATAGAATG
6D              TGGACTACCCAGGATTGCAATTGCTCCATCTATACGGGTCATATCACTGGCCATAGAATG
9C              TGGACTACCCAGGATTGCAATTGCTCCACCTATACGGGTCATATCACTGGCCATAGAATG
2C              TGGACTACCCAGGATTGCAATTGCTCCACCTATACGGGTCATATCACTGGCCATAGAATG
5A              TGGACTACCCAGGATTGCAATTGCTCCATCTATACGGGTCATATCACTGGCCATAGAATG
8A              TGGACTACCCAGGATTGCAATTGCTCCATCTATACGGGTCATATCACTGGCCATAGAATG
3E              TGGACTACCCAGGATTGCAATTGCTCCATCTATACGGGTCATATCACTGGCCATAGAATG
4E              TGGACTACCCAGGATTGCAATTGCTCCATCTATACGGGTCATATCACTGGCCATAGAATG
10A             TGGACTACCCAGGATTGCAATTGCTCCATCTATACGGGTCATATCACTGGCCATAGAATG
7B              TGGACTACCCAGGATTGCAATTGCTCCATCTATACGGGTCATATCACTGGCCATAGAATG
6C              TGGACTACCCAGGATTGCAATTGCTCCATCTATACGGGTCATATCACTGGCCATAGAATG
8C              TGGACTACCCAGGATTGCAATTGCTCCATCTATACGGGTCATATCACTGGCCATAGAATG
1C              TGGACTACCCAGGATTGCAATTGCTCCATCTATACGGGTCATATCACTGGCCATAGAATG
4C              TGGACTACCCAGGATTGCAATTGCTCCATCTATACGGGTCATATCACTGGCCATAGAATG
7C              TGGACTACCCAGGATTGCAATTGCTCCATCTATACGGGTCATATCACTGGCCATAGAATG
10C             TGGACTACCCAGGATTGCAATTGCTCCATCTATACGGGTCATATCACTGGCCATAGAATG
5C              TGGACTACCCAGGATTGCAATTGCTCCATCTATACGGGTCATATCACTGGCCATAGAATG
3C              TGGACTACCCAGGATTGCAATTGCTCCATCTATACGGGTCATATCACTGGCCATAGAATG
                ************** ********* **** *********************** ******

7A              GCCTGGGACATGATGATGAACTGGAGTCCAACAACCACCCTAGTTCTCGCCCAGGTTATG
6B              GCCTGGGACATGATGATGAACTGGAGTCCAACAACCACCCTAGTTCTCGCCCAGGTTATG
9D              GCCTGGGACATGATGATGAACTGGAGTCCAACAACCACCCTAGTTCTCGCCCAGGTTATG
1D              GCCTGGGACATGATGATGAACTGGAGTCCAACAACCACCCTAGTTCTCGCCCAGGTTATG
8E              GCCTGGGACATGATGATGAACTGGAGTCCAACAACCACCCTAGTTCTCGCCCAGGTTATG
9A              GCCTGGGACATGATGATGAACTGGAGTCCAACAACCACCCTAGTTCTCGCCCAGGTTATG
10D             GCCTGGGACATGATGATGAACTGGAGTCCAACAACCACCCTAGTTCTCGCCCAGGTTATG
3D              GCCTGGGACATGATGATGAACTGGAGTCCAACAACCACCCTAGTTCTCGCCCAGGTTATG
4D              GCCTGGGACATGATGATGAACTGGAGTCCAACAACCACCCTAGTTCTCGCCCAGGTTATG
5D              GCCTGGGACATGATGATGAACTGGAGTCCAACAACCACCCTAGTTCTCGCCCAGGTTATG
10B             GCCTGGGACATGATGATGAACTGGAGTCCAACAACCACCCTAGTTCTCGCCCAGGTTATG
5E              GCCTGGGACATGATGATGAACTGGAGTCCAACAACCACCCTAGTTCTCGCCCAGGTTATG
1E              GCCTGGGACATGATGATGAACTGGAGTCCAACAACCACCCTAGTTCTCGCCCAGGTTATG
7D              GCCTGGGACATGATGATGAACTGGAGTCCAACAACCACCCTAGTTCTCGCCCAGGTTATG
2D              GCCTGGGACATGATGATGAACTGGAGTCCAACAACCACCCTAGTTCTCGCCCAGGTTATG
```

```
2E              GCCTGGGACATGATGATGAACTGGAGTCCAACAACCACCCTAGTTCTCGCCCAGGTTATG
6E              GCCTGGGACATGATGATGAACTGGAGTCCAACAACCACCCTAGTTCTCGCCCAGGTTATG
2B              GCCTGGGACATGATGATGAACTGGAGTCCAACAACCACCCTAGTTCTCGCCCAGGTTATG
3B              GCCTGGGACATGATGATGAACTGGAGTCCAACAACCACCCTAGTTCTCGCCCAGGTTATG
6D              GCCTGGGACATGATGATGAACTGGAGTCCAACAACCACCCTAGTTCTCGCCCAGGTTATG
9C              GCCTGGGACATGATGATGAACTGGAGTCCAACAACCACCCTAGTTCTCGCCCAGGTTATG
2C              GCCTGGGACATGATGATGAACTGGAGTCCAACAACCACCCTAGTTCTCGCCCAGGTTATG
5A              GCCTGGGACATGATGATGAACTGGAGTCCAACAACCACCCTAGTTCTCGCCCAGGTTATG
8A              GCCTGGGACATGATGATGAACTGGAGTCCAACAACCACCCTAGTTCTCGCCCAGGTTATG
3E              GCCTGGGACATGATGATGAACTGGAGTCCAACAACCACCCTAGTTCTCGCCCAGGTTATG
4E              GCCTGGGACATGATGATGAACTGGAGTCCAACAACCACCCTAGTTCTCGCCCAGGTTATG
10A             GCCTGGGACATGATGATGAACTGGAGTCCAACAACCACCCTAGTTCTCGCCCAGGTTATG
7B              GCCTGGGACATGATGATGAACTGGAGTCCAACAACCACCCTAGTTCTCGCCCAGGTTATG
6C              GCCTGGGACATGATGATGAACTGGAGTCCAACAACCACCCTAGTTCTCGCCCAGGTTATG
8C              GCCTGGGACATGATGATGAACTGGAGTCCAACAACCACCCTAGTTCTCGCCCAGGTTATG
1C              GCCTGGGACATGATGATGAACTGGAGTCCAACAACCACCCTAGTTCTCGCCCAGGTTATG
4C              GCCTGGGACATGATGATGAACTGGAGTCCAACAACCACCCTAGTTCTCGCCCAGGTTATG
7C              GCCTGGGACATGATGATGAACTGGAGTCCAACAACCACCCTAGTTCTCGCCCAGGTTATG
10C             GCCTGGGACATGATGATGAACTGGAGTCCAACAACCACCCTAGTTCTCGCCCAGGTTATG
5C              GCCTGGGACATGATGATGAACTGGAGTCCAACAACCACCCTAGTTCTCGCCCAGGTTATG
3C              GCCTGGGACATGATGATGAACTGGAGTCCAACAACCACCCTAGTTCTCGCCCAGGTTATG
                ************************************************************

7A              AGGATCCCAGGCACTCTGCTAGACCTACTAGCTGGAGGCCACTGGGGTGTCCTCGTGGGA
6B              AGGATCCCAGGTACTCTGCTAGACCTACTATCTGGAGGCCACTGGGGTGTCCTCGTGGGA
9D              AGGATCCCAGGTACTCTGCTAGACCTACTAGCTGGAGGCCACTGGGGTGTCCTCGTGGGA
1D              AGGATCCCAGGTACTCTGCTAGACCTACTAGCTGGAGGCCACTGGGGTGTCCTCGTGGGA
8E              AGGATCCCAGGTACTCTGCTAGACCTACTAGCTGGAGGCCACTGGGGTGTCCTCGTGGGA
9A              AGGATCCCAGGTACTCTGCTAGACCTACTAGCTGGAGGCCACTGGGGTGTCCTCGTGGGA
10D             AGGATCCCAGGTACTCTGCTAGACCTACTTGCTGGAGGCCACTGGGGTGTCCTCGTGGGA
3D              AGGATCCCAGGTACTCTGCTAGACCTACTTGCTGGAGGCCACTGGGGTGTCCTCGTGGGA
4D              AGGATCCCAGGTACTCTGCTAGACCTACTTGCTGGAGGCCACTGGGGTGCCCTCGTGGGA
5D              AGGATCCCAGGTACTCTGCTAGACCTACTTGCTGGAGGCCACTGGGGTGCCCTCGTGGGA
10B             AGGATCCCAGGTACTCTGCTAGACCTACTTGCTGGAGGCCACTGGGGTGTCCTCGTGGGA
5E              AGGATCCCAGGTACTCTGCTAGACCTACTTGCTGGAGGCCACTGGGGTGTCCTCGTGGGA
1E              AGGATCCCAGGTACTCTGCTAGACCTACTTGCTGGAGGCCACTGGGGTGTCCTCGTGGGA
7D              AGGATCCCAGGTACTCTGCTAGACCTACTTGCTGGAGGCCACTGGGGTGTCCTCGTGGGA
2D              AGGATCCCAGGTACTCTGCTAGACCTACTTGCTGGAGGCCACTGGGGTGTCCTCGTGGGA
2E              AGGATCCCAGGTACTCTGCTAGACCTACTTGCTGGAGGCCACTGGGGTGTCCTCGTGGGA
6E              AGGATCCCAGGTACTCTGCTAGACCTACTTGCTGGAGGCCACTGGGGTGTCCTCGTGGGA
2B              AGGATCCCAGGTACTCTGCTAGACCTACTTGCTGGAGGCCACTGGGGTGTCCTCGTGGGA
3B              AGGATCCCAGGTACTCTGCTAGACCTACTTGCTGGAGGCCACTGGGGCGTCCTCGTGGGA
6D              AGGATCCCAGGTACTCTGCTAGACCTACTTGCTGGAGGCCACTGGGGCGTCCTCGTGGGA
9C              AGGATCCCAGGTACTCTGGTAAACCTACTTGCTGGAGGCCACTGGGGCGTCCTCGTGGGA
2C              AGGATCCCAGGTACTCTGCTAGACCTACTTGCTGGAGGCCACTGGGGCGTCCTCGTGGGA
5A              AGGATCCCAGGTACTCTGCTAGACCTACTTGCTGGAGGCCACTGGGGCGTCCTCGTGGGA
8A              AGGATCCCAGGTACTCTGCTAGACCTACTTGCTGGAGGCCACTGGGGCGTCCTCGTGGGA
3E              AGGATCCCAGGTACTCTGCTAGACCTACTTGCTGGAGGCCACTGGGGCGTCCTCGTGGGA
4E              AGGATCCCAGGTACTCTGCTAGACCTACTTGCTGGAGGCCACTGGGGCGTCCTCGTGGGA
10A             AGGATCCCAGGTACTCTGCTAGACCTACTTGCTGGAGGCCACTGGGGCGTCCTCGTGGGA
7B              AGGATCCCAGGTACTCTGCTAGACCTACTTGCTGGAGGCCACTGGGGCGTCCTCGTGGGA
6C              AGGATCCCAGGTACTCTGCTAGACCTACTTGCTGGAGGCCACTGGGGCGCCCTCGTGGGA
8C              AGGATCCCAGGTACTCTGCTAGACCTACTTGCTGGGGGCCACTGGGGCGCCCTCGTGGGA
1C              AGGATCCCAGGTACTCTGCTAGACCTACTTGCTGGAGGCCACTGGGGCGCCCTCGTGGGA
4C              AGGATCCCAGGTACTCTGCTAGACCTACTTGCTGGAGGCCACTGGGGCGCCCTCGTGGGA
7C              AGGATCCCAGGTACTCTGCTAGACCTACTTGCTGGAGGCCACTGGGGCGCCCTCGTGGGA
10C             AGGATCCCAGGTACTCTGCTAGACCTACTTGCTGGAGGCCACTGGGGCGCCCTCGTGGGA
```

```
5C          AGGATCCCAGGTACTCTGCTAGACCTACTTGCTGGAGGCCACTGGGGCGCCCTCGTGGGA
3C          AGGATCCCAGGTACTCTGCTAGACCTACTTGCTGGAGGCCACTGGGGCGCCCTCGTGGGA
            *********** ****** ** *******  **** *********** * **********

7A          GTGGCCTATTTCAGCATGCAATCTAATTGGGCAAAAGTCATCCTAGTCCTATTTCTCTTT
6B          GTGGCCTATTTCAGCATGCAATCTAATTGGGCAAAAGTCATCCTAGTCCTATTTCTCTTT
9D          GTGGCCTATTTCAGCATGCAATCTAATTGGGCAAAAGTCATCCTAGTCCTATTTCTCTTT
1D          GTGGCCTATTTCAGCATGCAATCTAATTGGGCAAAAGTCATCCTAGTCCTATTTCTCTTT
8E          GTGGCCTATTTCAGCATGCAATCTAATTGGGCAAAAGTCATCCTAGTCCTATTTCTCTTT
9A          GTGGCCTATTTCAGCATGCAGTCTAATTGGGCAAAAGTCATCCTAGTCCTATTTCTCTTT
10D         GTGGCCTATTTCAGCATGCAATCTAATTGGGCAAAAGTCATCCTAGTCCTATTTCTCTTT
3D          GTGGCCTATTTCAGCATGCAATCTAATTGGGCAAAAGTCATCCTAGTCCTATTTCTCTTT
4D          GTGGCCTATTTCAGCATGCAATCTAATTGGGCAAAAGTCATCCTAGTCCTATTTCTCTTT
5D          GTGGCCTATTTCAGCATGCAATCTAATTGGGCAAAAGTCATCCTAGTCCTATTTCTCTTT
10B         GTGGCCTATTTCAGCATGCAATCTAATTGGGCAAAAGTCATCCTAGTCCTATTTCTCTTT
5E          GTGGCCTATTTCAGCATGCAATCTAATTGGGCAAAAGTCATCCTAGTCCTATTTCTCTTT
1E          GTGGCCTATTTCAGCATGCAATCTAATTGGGCAAAAGTCATCCTAGTCCTATTTCTCTTT
7D          GTGGCCTATTTCAGCATGCAATCTAATTGGGCAAAAGTCATCCTAGTCCTATTTCTCTTT
2D          GTGGCCTATTTCAGCATGCAATCTAATTGGGCAAAAGTCATCCTAGTCCTATTTCTCTTT
2E          GTGGCCTATTTCAGCATGCAATCTAATTGGGCAAAAGTCATCCTAGTCCTATTTCTCTTT
6E          GTGGCCTATTTCAGCATGCAATCTAATTGGGCAAAAGTCATCCTAGTCCTATTTCTCTTT
2B          GTGGCCTATTTCAGCATGCAGTCTAATTGGGCAAAAGTCATCCTAGTCCTATTTCTCTTT
3B          GTGGCCTATTTCAGCATGCAATCTAATTGGGCGAAAGTCATCCTAGTTCTATTTCTCTTT
6D          GTGGCCTATTTCAGCATGCAATCCAATTGGGCGAAAGTCATCCTAGTTCTATTTCTCTTT
9C          GTGGCCTATTTCAGCATGCAATCCAATTGGGCCAAAGTCATCCTAGTTCTATTTCTCTTT
2C          GTGGCCTATTTCAGCATGCAATCCAATTGGGCGAAAGTCATCCTAGTTCTATTTCTCTTT
5A          GTGGCCTATTTCAGCATGCAATCTAATTGGGCGAAAGTCATCCTAGTTCTATTTCTCTTT
8A          GTGGCCTATTTCAGCATGCAATCCAATTGGGCGAAAGTCATCCTAGTTCTATTTCTCTTT
3E          GTGGCCTATTTCAGCATGCAATCCAATTGGGCGAAAGTCATCCTAGTTCTATTTCTCTTT
4E          GTGGCCTATTTCAGCATGCAATCCAATTGGGCGAAAGTCATCCTAGTTCTATTTCTCTTT
10A         GTGGCCTATTTCAGCATGCAATCCAATTGGGCGAAAGTCATCCTAGTTCTATTTCTCTTT
7B          GTGGCCTATTTCAGCATGCAATCCAATTGGGCGAAAGTCATCCTAGTTCTATTTCTCTTT
6C          GTGGCCTATTTCAGCATGCAATCCAATTGGGCGAAAGTCATCCTAGTTCTATTTCTCTTT
8C          GTGGCCTATTTCAGCATGCAATCCAATTGGGCGAAAGTCATCCTAGTTCTATTTCTCTTT
1C          GTGGCCTATTTCAGCATGCAATCCAATTGGGCGAAAGTCATCCTAGTTCTATTTCTCTTT
4C          GTGGCCTATTTCAGCATGCAATCCAATTGGGCGAAAGTCATCCTAGTTCTATTTCTCTTT
7C          GTGGCCTATTTCAGCATGCAATCCAATTGGGCGAAAGTCATCCTAGTTCTATTTCTCTTT
10C         GTGGCCTATTTCAGCATGCAATCCAATTGGGCGAAAGTCATCCTAGTTCTATTTCTCTTT
5C          GTGGCCTATTTCAGCATGCAATCCAATTGGGCGAAAGTCATCCTAGTTCTATTTCTCTTT
3C          GTGGCCTATTTCAGCATGCAATCCAATTGGGCGAAAGTCATCCTAGTTCTATTTCTCTTT
            ******************** ** ******** ************** ************

7A          GCAGGGGTTGACGCCAGCACTCGTGTGTCAGGGGGCGTGGTTTCTCACACCACCGCTGGC
6B          GCAGGGGTTGATGCCAGCACTCGTGTGTCAGGGGGCGTGGTTTCTCACACCACCGCTGGC
9D          GCAGGGGTTGACGCCAGCACTCGTGTGTCAGGGGGCGTGGTTTCTCACACCACCGCTGGC
1D          GCAGGGGTTGATGCCAGCACTCGTGTGTCAGGGGGCGTGGTTTCTCACACCACCGCTGGC
8E          GCAGGGGTTGATGCCAGCACTCGTGTGTCAGGGGGTGTGGTTTCTCACACCACCGCTGGC
9A          GCAGGGGTTGACGCCAGCACTCGTGTGTCAGGGGGCGTGGTTTCTCACACCACCGCTGGC
10D         GCAGGGGTTGACGCCAGCACTCGTGTGTCAGGGGGCGTGGTTTCTCACACCACCGCTGGC
3D          GCAGGGGTTGACGCCAGCACTCGTGTGTCAGGGGGCGTGGTTTCTCACACCACCGCTGGC
4D          GCAGGGGTTGACGCCAGCACTCGTGTGTCAGGGGGCGTGGTTTCTCACACCACCGCTGGC
5D          GCAGGGGTTGACGCCAGCACTCGTGTGTCAGGGGGCGTGGTTTCTCACACCACCGCTGGC
10B         GCAGGGGTTGACGCCAGCACTCGTGTGTCAGGGGGCGTGGTTTCTCACACCACCGCTGGC
5E          GCAGGGGTTGACGCCAGCACTCGTGTGTCAGGGGGCGTGGTTTCTCACACCACCGCTGGC
1E          GCAGGGGTTGACGCCAGCACTCGTGTGTCAGGGGGCGTGGTTTCTCACACCACCGCTGGC
7D          GCAGGGGTTGACGCCAGCACTCGTGTGTCAGGGGGCGTGGTTTCTCACACCACCGCTGGC
2D          GCAGGGGTTGACGCCAGCACTCGTGTGTCAGGGGGCGTGGTTTCTCACACCACCGCTGGC
```

```
2E              GCAGGGGTTGACGCCAGCACTCGTGTGTCAGGGGGCGTGGTTTCTCACACCACCGCTGGC
6E              GCAGGGGTTGACGCCAGCACTCGTGTGTCAGGGGGCGTGGTTTCTCACACCACCGCTGGC
2B              GCAGGGGTAGACGCCGAAACTCATGTGTCAGGGGGTGTGGTTTCTCACAGCACCTCTGGG
3B              GCAGGGGTAGACGCCCACACTCATGTGTCAGGGGGTGTGGTTTCTCACAGCACCTATGGG
6D              GCAGGGGTAGACGCCCACACTCATGTGTCAGGTGGTGTGGTTTCTCACAGCACCTATGGG
9C              GCAGGGGTAGACGCCCACACTCCTGTGTCAGGGGGTGTGGTTTCTCACACCACCTATGGG
2C              GCAGGGGTAGACGCCCACACTCATGTGTCAGGTGGTGTGGTTTCTCACAGCACCTATGGG
5A              GCAGGGGTAGACGCCCACACTCATGTGTCAGGGGGTGTGGTTTCTCACAGCACCAATGGG
8A              GCAGGGGTAGACGCCCACACTCATGTGTCAGGTGGTGTGGTTTCTCACAGCACCTATGGG
3E              GCAGGGGTAGACGCCCACACTCATGTGTCAGGTGGTGTGGTTTCTCACAGCACCTATGGG
4E              GCAGGGGTAGACGCCCACACTCATGTGTCAGGTGGTGTGGTTTCTCACAGCACCTATGGG
10A             GCAGGGGTAGACGCCCACACTCATGTGTCAGGTGGTGTGGTTTCTCACAGCACCTATGGG
7B              GCAGGGGTAGACGCCCACACTCGTGTGTCAGGTGGTGTGGTTTCTCACAGCACCTATGGG
6C              GCAGGGGTAGACGCCCACGCTCATGTGTCAGGTGGTGTGGTTTCTCACAGCACCTATGGG
8C              GCAGGGGTAGACGCCCACGCTCATGTGTCAGGTGGTGTGGTTTCTCACAGCACCTATGGG
1C              GCAGGGGTAGACGCCCACGCTCATGTGTCAGGTGGTGTGGTTTCTCACAGCACCTATGGG
4C              GCAGGGGTAGACGCCCACGCTCATGTGTCAGGTGGTGTGGTTTCTCACAGCACCTATGGG
7C              GCAGGGGTAGACGCCCACGCTCATGTGTCAGGTGGTGTGGTTTCTCACAGCACCTATGGG
10C             GCAGGGGTAGACGCCCACGCTCATGTGTCAGGTGGTGTGGTTTCTCACAGCACCTATGGG
5C              GCAGGGGTAGACGCCCACGCTCATGTGTCAGGTGGTGTGGTTTCTCACAGCACCTATGGG
3C              GCAGGGGTAGACGCCCACGCTCATGTGTCAGGTGGTGTGGTTTCTCACAGCACCTATGGG
                ******** ** ***    *** ********* ** ************* ****  ***

7A              CTTGCCGGACTTCTGTCCCCTGGACCCAAGCAGAATCTACATCTCATCAACAGCAATGGG
6B              CTTGCCGGACTTCTGTCCCCTGGACCCAAGCAGAATCTACATCTCATCAACAGCAATGGG
9D              CTTGCCGGACTTCTGTCCCCTGGACCCAAGCAGAATCTACATCTCATCAACAGCAATGGG
1D              CTTGCCGGACTTCTGTCCCCTGGACCCAAGCAGAATCTACATCTCATCAACAGCAATGGG
8E              CTTGCCGGACTTCTGTCCCCTGGACCCAAGCAGAATCTACATCTCATCAACAGCAATGGG
9A              CTTGCCGGACTTCTGTCCCCTGGACCCAAGCAGAATCTACATCTCATCAACAGCAATGGG
10D             CTTGCCGGACTTCTTTCCCCTGGACCCAAGCAGAATCTACATCTCATCAACAGCAACGGG
3D              CTTGCCGGACTTCTTTCCCCTGGACCCAAGCAGAATCTACATCTCATCAACAGCAACGGG
4D              CTTGCCGGACTTCTTTCCCCTGGACCCAAGCAGAATCTACATCTCATCAACAGCAACGGG
5D              CTTGCCGGACTTCTTTCCCCTGGACCCAAGCAGAATCTACATCTCATCAACAGCAACGGG
10B             CTTGCCGGACTTCTTTCCCCTGGACCCAAGCAGAATCTACATCTCATCAACAGCAACGGG
5E              CTTGCCGGACTTCTTTCCCCTGGACCCAAGCAGAATCTACATCTCATCAACAGCAACGGG
1E              CTTGCCGGACTTCTTTCCCCTGGACCCAAGCAGAATCTACATCTCATCAACAGCAACGGG
7D              CTTGCCGGACTTCTTTCCCCTGGACCCAAGCAGAATCTACATCTCATCAACAGCAACGGG
2D              CTTGCCGGACTTCTTTCCCCTGGACCCAAGCAGAATCTACATCTCATCAACAGCAACGGG
2E              CTTGCCGGACTTCTTTCCCCTGGACCCAAGCAGAATCTACATCTCATCAACAGCAACGGG
6E              CTTGCCGGACTTCTTTCCCCTGGACCCAAGCAGAATCTACATCTCATCAACAGCAACGGG
2B              CTTGCCGACTTTCTTACCCCTGGACCCAGCCAGAAACTACATCTCATCAACAGCAACGGG
3B              TTTGCCAGCCTTCTTACCCCTGGACCCAGCCAGAAGCTACATCTCATCAACAGCAACGGG
6D              TTTGCCAGCCTTCTTACCCCTGGACCCAGCCAGAAGCTACATCTCATCAACAGCAACGGG
9C              TTTGCCAGCCTTCTTACCCCTGGACCCAGCCAGAAGCTACATCTCATCAACAGCAACGGG
2C              TTTGCCAGCCTTCTTACCCCTGGACCCAGCCAGAAGCTACATCTCATCAACAGCAACGGG
5A              TTTGCCAGCCTTCTTACCCCTGGACCCAGCCAGAAGCTACATCTCATCAACAGCAACGGG
8A              TTTGCCAGCCTTCTTACCCCTGGACCCAGCCAGAAGCTACATCTCATCAACAGCAATGGG
3E              TTTGCCAGCCTTCTTACCCCTGGACCCAGCCAGAAGCTACATCTCATCAACAGCAACGGG
4E              TTTGCCAGCCTTCTTACCCCTGGACCCAGCCAGAAGCTACATCTCATCAACAGCAACGGG
10A             TTTGCCAGCCTTCTTACCCCTGGACCCAGCCAGAAGCTACATCTCATCAACAGCAACGGG
7B              TTTGCCAGCCTTCTTACCCCTGGACCCAGCCAGAAGCTACATCTCATCAACAGCAACGGG
6C              TTTGCCAGCCTTCTTACCCCTGGACCCAGCCAGAAGCTACATCTCATCAACAGCAACGGG
8C              TTTGCCAGCCTTCTTACCCCTGGACCCAGCCAGAAGCTACATCTCATCAACAGCAACGGG
1C              TTTGCCAGCCTTCTTACCCCTGGACCCAGCCAGAAGCTACATCTCATCAACAGCAACGGG
4C              TTTGCCAGCCTTCTTACCCCTGGACCCAGCCAGAAGCTACATCTCATCAACAGCAACGGG
7C              TTTGCCAGCCTTCTTACCCCTGGACCCAGCCAGAAGCTACATCTCATCAACAGCAACGGG
10C             TTTGCCAGCCTTCTTACCCCTGGACCCAGCCAGAAGCTACATCTCATCAACAGCAACGGG
```

```
5C              TTTGCCAGCCTTCTTACCCCTGGACCCAGCCAGAAGCTACATCTCATCAACAGCAACGGG
3C              TTTGCCAGCCTTCTTACCCCTGGACCCAGCCAGAAGCTACATCTCATCAACAGCAACGGG
                 *****    ****  ************  ***** ******************** ***

7A              AGCTGGCATATAAACAGGACTGCCCTCAATTGCAATGACAGCTTAAACACTGGGTTCTTA
6B              AGCTGGCATATAAACAGGACTGCCCTCAATTGCAATGACAGCTTAAACACTGGGTTCTTA
9D              AGCTGGCATATAAACAGGACTGCCCTCAATTGCAATGACAGCTTAAACACTGGGTTCTTA
1D              AGCTGGCATATAAACAGGACTGCCCTCAATTGCAATGACAGCTTAAACACTGGGTTCTTA
8E              AGCTGGCATATAAACAGGACTGCCCTCAATTGCAATGACAGCTTAAACACTGGGTTCTTA
9A              AGCTGGCATATAAACAGGACTGCCCTCAATTGCAATGACAGCTTAAACACTGGGTTCTTA
10D             AGCTGGCATATAAACAGGACTGCCCTCAATTGCAATGACAGCTTAAACACTGGGTTCTTA
3D              AGCTGGCATATAAACAGGACTGCCCTCAATTGCAATGACAGCTTAAACACTGGGTTCTTA
4D              AGCTGGCATATAAACAGGACTGCCCTCAATTGCAATGACAGCTTAAACACTGGGTTCTTA
5D              AGCTGGCATATAAACAGGACTGCCCTCAATTGCAATGACAGCTTAAACACTGGGTTCTTA
10B             AGCTGGCATATAAACAGGACTGCCCTCAATTGCAATGACAGCTTAAACACTGGGTTCTTA
5E              AGCTGGCATATAAACAGGACTGCCCTCAATTGCAATGACAGCTTAAACACTGGGTTCTTA
1E              AGCTGGCATATAAACAGGACTGCCCTCAATTGCAATGACAGCTTAAACACTGGGTTCTTA
7D              AGCTGGCATATAAACAGGACTGCCCTCAATTGCAATGACAGCTTAAACACTGGGTTCTTA
2D              AGCTGGCATATAAACAGGACTGCCCTCAATTGCAATGACAGCTTAAACACTGGGTTCTTA
2E              AGCTGGCATATAAACAGGACTGCCCTCAATTGCAATGACAGCTTAAACACTGGGTTCTTA
6E              AGCTGGCATATAAACAGGACTGCCCTCAATTGCAATGACAGCTTAAACACTGGGTTCTTA
2B              AGCTGGCATATAAACAGGACTGCCCTCAATTGCAATGACAGCTTAAACACTGGGTTCTTA
3B              AGCTGGCATATAAATAGGACTGCCCTCAACTGCAATGACAGCTTAAACACTGGGTTCTTA
6D              AGCTGGCATATAAATAGGACTGCCCTCAATTGCAATGACAGCTTAAACACTGGGTTCTTA
9C              AGCTGGCATATAAATAGGACTGCCCTCAATTGCAATGACAGCTTAAACACTGGGTTCTTA
2C              AGCTGGCATATAAATAGGACTGCCCTCAATTGCAATGACAGCTTAAACACTGGGTTCTTA
5A              AGCTGGCATATAAATAGGACTGCCCTCAACTGCAATGACAGCTTAAACACTGGGTTCTTA
8A              AGCTGGCATATAAACAGGACTGCCCTCAATTGCAATGACAGCTTAAACACTGGGTTCTTA
3E              AGCTGGCATATAAATAGGACTGCCCTCAATTGCAATGACAGCTTAAACACTGGGTTCTTA
4E              AGCTGGCATATAAATAGGACTGCCCTCAATTGCAATGACAGCTTAAACACTGGGTTCTTA
10A             AGCTGGCATATAAATAGGACTGCCCTCAATTGCAATGACAGCTTAAACACTGGGTTCTTA
7B              AGCTGGCATATAAATAGGACTGCCCTCAATTGCAATGACAGCTTAAACACTGGGTTCTTA
6C              AGCTGGCATATAAATAGGACTGCCCTCAATTGCAATGACAGCTTAAACACTGGGTTCTTA
8C              AGCTGGCATATAAATAGGACTGCCCTCAATTGCAATGACAGCTTAAACACTGGGTTCTTA
1C              AGCTGGCATATAAATAGGACTGCCCTCAATTGCAATGACAGCTTAAACACTGGGTTCTTA
4C              AGCTGGCATATAAATAGGACTGCCCTCAATTGCAATGACAGCTTAAACACTGGGTTCTTA
7C              AGCTGGCATATAAATAGGACTGCCCTCAATTGCAATGACAGCTTAAACACTGGGTTCTTA
10C             AGCTGGCATATAAATAGGACTGCCCTCAATTGCAATGACAGCTTAAACACTGGGTTCTTA
5C              AGCTGGCATATAAATAGGACTGCCCTCAATTGCAATGACAGCTTAAACACTGGGTTCTTA
3C              AGCTGGCATATAAATAGGACTGCCCTCAATTGCAATGACAGCTTAAACACTGGGTTCTTA
                ************** ************** ******************************

7A              GCTAGCTTGTTCTACGCTCACAAGTTTAACAGCTCAGGGTGTCCCGAACGGCTAGCATCA
6B              GCTAGCTTGTTCTACGCTCACAAGTTTAACAGCTCAGGGTGTCCCGAACGGCTAGCATCA
9D              GCTAGCTTGTTCTACGCTCACAAGTTTAACAGCTCAGGGTGTCCCGAACGGCTAGCATCA
1D              GCTAGCTTGTTCTACGCTCACAAGTTTAACAGCTCAGGGTGTCCCGAACGGCTAGCATCA
8E              GCTAGCTTGTTCTACGCTCACAAGTTTAACAGCTCAGGGTGTCCCGAACGGCTAGCATCA
9A              GCTAGCTTGTTCTACGCTCACAAGTTTAACAGCTCAGGGTGTCCCGAACGGCTAGCATCA
10D             GCTAGCTTGTTCTACGCTCACAAGTTTAACAGCTCAGGGTGTCCCGAACGGCTAGCATCA
3D              GCTAGCTTGTTCTACGCTCACAGGTTTAACAGCTCAGGGTGTCCCGAACGGCTAGCACCA
4D              GCTAGCTTGTTCTACGCTCACAGGTTTAACAGCTCAGGGTGTCCCGAACGGCTAGCACCA
5D              GCTAGCTTGTTCTACGCTCACAGGTTTAACAGCTCAGGGTGTCCCGAACGGCTAGCACCA
10B             GCTAGCTTGTTCTACGCTCACAGGTTTAACAGCTCAGGGTGTCCCGAACGGCTAGCACCA
5E              GCTAGCTTGTTCTACGCTCACAGGTTTAACAGCTCAGGGTGTCCCGAACGGCTAGCACCA
1E              GCTAGCTTGTTCTACGCTCACAGGTTTAACAGCTCAGGGTGTCCCGAACGGCTAGCACCA
7D              GCTAGCTTGTTCTACGCTCACAGGTTTAACAGCTCAGGGTGTCCCGAACGGCTAGCACCA
2D              GCTAGCTTGTTCTACGCTCACAGGTTTAACAGCTCAGGGTGTCCCGAACGGCTAGCACCA
```

```
2E          GCTAGCTTGTTCTACGCTCACAGGTTTAACAGCTCAGGGTGTCCCGAACGGCTAGCACCA
6E          GCTAGCTTGTTCTACGCTCACAGGTTTAACAGCTCAGGGTGTCCCGAACGGCTAGCACCA
2B          GCTAGCTTGTTCTACACTCACAAGTTTAACAGCTCAGGGTGTCCCGAACGGCTAGCATCA
3B          GCTAGCTTGTTCTACGCTCATAAGTTTAACAGCTCAGGGTGTCCCGAACGGCTAGCATCA
6D          GCTAGCTTGTTCTACGCTCATAAGTTTAACAGCTCAGGGTGTCCCGAACGGCTAGCATCT
9C          GCTAGCCTGTTCTACGCTCATAAGTTTAACAGCTCAGGGTGTCCCGAACGGCTAGCATCA
2C          GCTAGCCTGTTCTACGCTCATAAGTTTAACAGCTCAGGGTGTCCCGAACGGCTAGCATCA
5A          GCTAGCTTGTTCTACGCTCATAAGTTTAACAGCTCAGGGTGTCCCGAACGGCTAGCATCA
8A          GCTAGCTTGTTCTACGCTCATAAGTTTAACAGCTCAGGGTGTCCCGAACGGCTAGCATCA
3E          GCTAGCTTGTTCTACGCTCATAAGTTTAACAGCTCAGGGTGTCCCGAACGGCTAGCATCT
4E          GCTAGCTTGTTCTACGCTCATAAGTTTAACAGCTCAGGGTGTCCCGAACGGCTAGCATCT
10A         GCTAGCTTGTTCTACGCTCATAAGTTTAACAGCTCAGGGTGTCCCGAACGGCTAGCATCA
7B          GCTAGCTTGTTCTACGCTCATAAGTTTAACAGCTCAGGGTGTCCCGAACGGCTAGCATCA
6C          GCTAGCTTGTTCTACGCTCATAAGTTTAACAGCTCAGGGTGTCCCGAACGGCTAGCATCA
8C          GCTAGCTTGTTCTACGCTCATAAGTTTAACAGCTCAGGGTGTCCCGAACGGCTAGCATCA
1C          GCTAGCTTGTTCTACGCTCATAAGTTTAACAGCTCAGGGTGTCCCGAACGGCTAGCATCA
4C          GCTAGCTTGTTCTACGCTCATAAGTTTAACAGCTCAGGGTGTCCCGAACGGCTAGCATCA
7C          GCTAGCTTGTTCTACGCTCATAAGTTTAACAGCTCAGGGTGTCCCGAACGGCTAGCATCA
10C         GCTAGCTTGTTCTACGCTCATAAGTTTAACAGCTCAGGGTGTCCCGAACGGCTAGCATCA
5C          GCTAGCTTGTTCTACGCTCATAAGTTTAACAGCTCAGGGTGTCCCGAACGGCTAGCATCA
3C          GCTAGCTTGTTCTACGCTCATAAGTTTAACAGCTCAGGGTGTCCCGAACGGCTAGCATCA
            ****** ******** **** * ********************************** *

7A          TGCAAAAGCCTTGACAGTTTTCCCCAAGGCTGGGGCCCACTTGGGGTCGCTAACATCAGC
6B          TGCAGAAGCCTTGACAGTTTTCCCCAAGGCTGGGGCCCACTTGGGGTCGCTAACATCAGC
9D          TGCAGAAGCCTTGACAGTTTTGCCCAAGGCTGGGGCCCACTTGGGGTCGCTAACATCAGC
1D          TGCAAAAGCCTTGACAGTTTTCCCCAAGGCTGGGGCCCACTTGGGGTCGCTAACATCAGC
8E          TGCAGAAGCCTTGACAGTTTTCCCCAAGGCTGGGGCCCACTTGGGGTCGCTAACATCAGC
9A          TGCAAAAGCCTTGACAGTTTTGACCAAGGCTGGGGCCCACTTGGGGTCGCTAACATCAGC
10D         TGCCGAAGCCTTGACAGTTTTGACCAAGGCTGGGGCCCACTTGGGGTCGCTAACATCAGC
3D          TGCCGAAGCCTTGACAGTTTTGACCAAGGCTGGGGCCCACTTGGGGTCGCTAACATCAGC
4D          TGCCGAAGCCTTGACAGTTTTGACCAAGGCTGGGGCCCACTTGGGGTCGCTAACATCAGC
5D          TGCCGAAGCCTTGACAGTTTTGACCAAGGCTGGGGCCCACTTGGGGTCGCTAACATCAGC
10B         TGCCGAAGCCTTGACAGTTTTGACCAAGGCTGGGGCCCACTTGGGGTCGCTAACATCAGC
5E          TGCCGAAGCCTTGACAGTTTTGACCAAGGCTGGGGCCCACTTGGGGTCGCTAACATCAGC
1E          TGCCGAAGCCTTGACAGTTTTGACCAAGGCTGGGGCCCACTTGGGGTCGCTAACATCAGC
7D          TGCCGAAGCCTTGACAGTTTTGACCAAGGCTGGGGCCCACTTGGGGTCGCTAACATCAGC
2D          TGCCGAAGCCTTGACAGTTTTGACCAAGGCTGGGGCCCACTTGGGGTCGCTAACATCAGC
2E          TGCCGAAGCCTTGACAGTTTTGACCAAGGCTGGGGCCCACTTGGGGTCGCTAACATCAGC
6E          TGCCGAAGCCTTGACAGTTTTGACCAAGGCTGGGGCCCACTTGGGGTCGCTAACATCAGC
2B          TGCAGGAGCCTTGACAGTTTTGACCAAGGCTGGGGCCCACTTGGGGTCGCTAACATCAGC
3B          TGCAAGAGCCTTGACAGTTTTGACCAAGGCTGGGGCCCACTTGGGGTCGCTAACATCAGC
6D          TGCAAGAGCCTTGACAGTTTTGACCAAGGCTGGGGCCCACTTGGGGTCGCTAACATCAGC
9C          TGCAAGAGCCTTGACAGTTTTGACCAAGGCTGGGGCCCACTTGGGGTCGCTAACATCAGC
2C          TGCAAGAGCCTTGACAGTTTTGACCAAGGCTGGGGCCCACTTGGGGTCGCTAACATCAGC
5A          TGCAAGAGCCTTGACAGTTTTGACCAAGGCTGGGGCCCACTTGGGGTCGCTAACATCAGC
8A          TGCAAGAGCCTTGACAGTTTTGACCAAGGCTGGGGCCCACTTGGGGTCGCTAACATCAGC
3E          TGCAAGAGCCTTGACAGTTTTGACCAAGGCTGGGGCCCACTTGGGGTCGCTAACATCAGC
4E          TGCAAGAGCCTTGACAGTTTTGACCAAGGCTGGGGCCCACTTGGGGTCGCTAACATCAGC
10A         TGCAAGAGCCTTGACAGTTTTGACCAAGGCTGGGGCCCACTTGGGGTCGCTAACATCAGC
7B          TGCAAGAGCCTTGACAGTTTTGACCAAGGCTGGGGCCCACTTGGGGTCGCTAACATCAGC
6C          TGCAAGAGCCTTGACAGTTTTGACCAAGGCTGGGGCCCACTTGGGGTCGCTAACATCAGC
8C          TGCAAGAGCCTTGACAGTTTTGACCAAGGCTGGGGCCCACTTGGGGTCGCTAACATCAGC
1C          TGCAAGAGCCTTGACAGTTTCGACCAAGGCTGGGGCCCACTTGGGGTCGCTAACATCAGC
4C          TGCAAGAGCCTTGACAGTTTTGACCAAGGCTGGGGCCCACTTGGGGTCGCTAACATCAGC
7C          TGCAAGAGCCTTGACAGTTTTGACCAAGGCTGGGGCCCACTTGGGGTCGCTAACATCAGC
10C         TGCAAGAGCCTTGACAGTTTTGACCAAGGCTGGGGCCCACTTGGGGTCGCTAACATCAGC
```

```
5C              TGCAAGAGCCTTGACAGTTTTGACCAAGGCTGGGGCCCACTTGGGGTCGCTAACATCAGC
3C              TGCAAGAGCCTTGACAGTTTTGACCAAGGCTGGGGCCCACTTGGGGTCGCTAACATCAGC
                ***   **************   *************************************

7A              GGCCCATCTGATGACAGGCCTTATTGCTGGCACTACGCGCCCCGGCCGTGTGCGATTGTG
6B              GGCCCATCTGATGACAGGCCTTATTGCTGGCACTACGCGCCCCGGCCGTGTGCGATTGTG
9D              GGCCCATCTGATGACAGGCCTTACTGCTGGCACTACGCGCCCCGGCCGTGTGCGATTGTG
1D              GGCCCATCTGAAGACAGGCCTTATTGCTGGCACTACGCGCCCCGGCCGTGTGCGATTGTG
8E              GGCCCATCTGATGACAGGCCTTATTGCTGGCACTACGCGCCCCGGCCGTGTGCGATTGTG
9A              GGCCCATCTGATGACAGGCCTTATTGCTGGCACTACGCGCCTCGGCCGTGTGCGATTGAG
10D             GGCCCATCTGATGACAGGCCTTATTGCTGGCACTACGCGCCCCGGCCGTGTGCGATTGTG
3D              GGCCCATCTGATGACAGGCCTTATTGCTGGCACTACGCGCCTCGGCCGTGTGCGATTGAG
4D              GGCCCATCTGATGACAGGCCTTATTGCTGGCACTACGCGCCTCGGCCGTGTGCGATTGAG
5D              GGCCCATCTGATGACAGGCCTTATTGCTGGCACTACGCGCCTCGGCCGTGTGCGATTGAG
10B             GGCCCATCTGATGACAGGCCTTATTGCTGGCACTACGCGCCTCGGCCGTGTGCGATTGAG
5E              GGCCCATCTGATGACAGGCCTTATTGCTGGCACTACGCGCCTCGGCCGTGTGCGATTGAG
1E              GGCCCATCTGATGACAGGCCTTATTGCTGGCACTACGCGCCTCGGCCGTGTGCGATTGAG
7D              GGCCCATCTGATGACAGGCCTTATTGCTGGCACTACGCGCCTCGGCCGTGTGCGATTGAG
2D              GGCCCATCTGATGACAGGCCTTATTGCTGGCACTACGCGCCTCGGCCGTGTGCGATTGAG
2E              GGCCCATCTGATGACAGGCCTTATTGCTGGCACTACGCGCCTCGGCCGTGTGCGATTGAG
6E              GGCCCATCTGATGACAGGCCTTATTGCTGGCACTACGCGCCTCGGCCGTGTGCGATTGAG
2B              GGCCCATCTGATGACAGGCCTTATTGCTGGCACTACGCGCCTCGGCCGTGTGCGATTGAG
3B              GGCCCATCTGAAGACAGGCCTTATTGCTGGCACTACGCGCCCCGGCCGTGTGCGATTGAG
6D              GGCCCATCTGATGACAGGCCTTATTGCTGGCACTACGCGCCTCGGCCGTGTGCGATTGAG
9C              GGCCCATCTGATGACAGGCCTTATTGCTGGCACTACGCGCCTCGGCCGTGTGCGATTGAG
2C              GGCCCATCTGATGACAGGCCTTATTGCTGGCACTACGCGCCTCGGCCGTGTGCGATTGAG
5A              GGCCCATCTGATGACAGGCCTTATTGCTGGCACTACGCGCCTCGGCCGTGTGCGATTGAG
8A              GGCCCATCTGATGACAGGCCTTATTGCTGGCACTACGCGCCTCGGCCGTGTGCGATTGAG
3E              GGCCCATCTGATGACAGGCCTTATTGCTGGCACTACGCGCCTCGGCCGTGTGCGATTGAG
4E              GGCCCATCTGATGACAGGCCTTATTGCTGGCACTACGCGCCTCGGCCGTGTGCGATTGAG
10A             GGCCCATCTGATGACAGGCCTTATTGCTGGCACTACGCGCCTCGGCCGTGTGCGATTGAG
7B              GGCCCATCTGATGACAGGCCTTATTGCTGGCACTACGCGCCTCGGCCGTGTGCGATTGAG
6C              GGCCCATCTGATGACAGGCCTTATTGCTGGCACTACGCGCCTCGGCCGTGTGCGATTGAG
8C              GGCCCATCTGATGACAGGCCTTATTGCTGGCACTACGCGCCTCGGCCGTGTGCGATTGAG
1C              GGCCCATCTGATGACAGGCCTTATTGCTGGCACTACGCGCCTCGGCCGTGTGCGATTGAG
4C              GGCCCATCTGATGACAGGCCTTATTGCTGGCACTACGCGCCTCGGCCGTGTGCGATTGAG
7C              GGCCCATCTGATGACAGGCCTTATTGCTGGCACTACGCGCCTCGGCCGTGTGCGATTGAG
10C             GGCCCATCTGATGACAGGCCTTATTGCTGGCACTACGCGCCTCGGCCGTGTGCGATTGAG
5C              GGCCCATCTGATGACAGGCCTTATTGCTGGCACTACGCGCCTCGGCCGTGTGCGATTGAG
3C              GGCCCATCTGATGACAGGCCTTATTGCTGGCACTACGCGCCTCGGCCGTGTGCGATTGAG
                *********** *********** ***************** **************** *

7A              CCGGCGGCCAGAGTGTGTGGTCCTGTGTACTGCTTTACTCCCAGCCCCGTCGTAGTCGGA
6B              CCGGCGGCCAGAGTGTGTGGTCCTGTGTACTGCTTTACTCCCAGCCCCGTCGTAGTCGGA
9D              CCGGCGGCCAGAGTGTGTGGTCCTGTGTACTGCTTTACTCCCAGCCCCGTCGTAGTCGGA
1D              CCGGCGGCCAGAGTGTGTGGTCCTGTGTACTGCTTTACTCCCAGCCCCGTCGTAGTCGGA
8E              CCGGCGGCCAGAGTGTGTGGTCCTGTGTACTGCTTTACTCCCAGCCCCGTCGTAGTCGGA
9A              CCGGCAGCCAGAGTGTGTGGTCCCGTGTACTGCTTTACTCCCAGCCCCGTCGTAGTCGGA
10D             CCGGCGGCCAGAGTGTGTGGTCCTGTGTACTGCTTTACTCCCAGCCCCGTCGTAGTCGGA
3D              CCGGCAGCCAGAGTGTGTGGTCCCGTGTACTGCTTTACTCCCAGCCCCGTCGTAGTCGGA
4D              CCGGCAGCCAGAGTGTGTGGTCCCGTGTACTGCTTTACTCCCAGCCCCGTCGTAGTCGGA
5D              CCGGCAGCCAGAGTGTGTGGTCCCGTGTACTGCTTTACTCCCAGCCCCGTCGTAGTCGGA
10B             CCGGCAGCCAGAGTGTGTGGTCCCGTGTACTGCTTTACTCCCAGCCCCGTCGTAGTCGGA
5E              CCGGCAGCCAGAGTGTGTGGTCCCGTGTACTGCTTTACTCCCAGCCCCGTCGTAGTCGGA
1E              CCGGCAGCCAGAGTGTGTGGTCCCGTGTACTGCTTTACTCCCAGCCCCGTCGTAGTCGGA
7D              CCGGCAGCCAGAGTGTGTGGTCCCGTGTACTGCTTTACTCCCAGCCCCGTCGTAGTCGGA
2D              CCGGCAGCCAGAGTGTGTGGTCCCGTGTACTGCTTTACTCCCAGCCCCGTCGTAGTCGGA
```

```
2E            CCGGCAGCCAGAGTGTGTGGTCCCGTGTACTGCTTTACTCCCAGCCCCGTCGTAGTCGGA
6E            CCGGCAGCCAGAGTGTGTGGTCCCGTGTACTGCTTTACTCCCAGCCCCGTCGTAGTCGGA
2B            CCGGCAGCCAGAGTGTGTGGTCCCGTGTACTGCTTTACTCCCAGCCCCGTCGTAGTCGGA
3B            CCGGCAGCCAGAGTGTGTGGTCCCGTGTACTGCTTTACTCCCAGCCCCGTCGTAGTCGGA
6D            CCGGCAGCCAGAGTGTGTGGTCCCGTGTACTGCTTTACTCCCAGCCCCGTCGTAGTCGGA
9C            CCGGCAGCCAGAGTGTGTGGTCCCGTGTACTGCTTTACTCCCAGCCCTGTCGTAGTCGGA
2C            CCGGCAGCCAGAGTGTGTGGTCCCGTGTACTGCTTTACTCCCAGCCCTGTCGTAGTCGGA
5A            CCGGCAGCCAGAGTGTGTGGTCCCGTGTACTGCTTTACTCCCAGCCCCGTCGTAGTCGGA
8A            CCGGCAGCCAGAGTGTGTGGTCCCGTGTACTGCTTTACTCCCAGCCCCGTCGTAGTCGGA
3E            CCGGCAGCCAGAGTGTGTGGTCCCGTGTACTGCTTTACTCCCAGCCCCGTCGTAGTCGGA
4E            CCGGCAGCCAGAGTGTGTGGTCCCGTGTACTGCTTTACTCCCAGCCCCGTCGTAGTCGGA
10A           CCGGCAGCCAGAGTGTGTGGTCCCGTGTACTGCTTTACTCCCAGCCCCGTCGTAGTCGGA
7B            CCGGCAGCCAGAGTGTGTGGTCCCGTGTACTGCTTTACTCCCAGCCCCGTCGTAGTCGGA
6C            CCGGCAGCCAGAGTGTGTGGTCCCGTGTACTGCTTTACTCCCAGCCCCGTCGTAGTCGGA
8C            CCGGCAGCCAGAGTGTGTGGTCCCGTGTACTGCTTTACTCCCAGCCCCGTCGTAGTCGGA
1C            CCGGCAGCCAGAGTGTGTGGTCCCGTGTACTGCTTTACTCCCAGCCCCGTCGTAGTCGGA
4C            CCGGCAGCCAGAGTGTGTGGTCCCGTGTACTGCTTTACTCCCAGCCCCGTCGTAGTCGGA
7C            CCGGCAGCCAGAGTGTGTGGTCCCGTGTACTGCTTTACTCCCAGCCCCGTCGTAGTCGGA
10C           CCGGCAGCCAGAGTGTGTGGTCCCGTGTACTGCTTTACTCCCAGCCCCGTCGTAGTCGGA
5C            CCGGCAGCCAGAGTGTGTGGTCCCGTGTACTGCTTTACTCCCAGCCCCGTCGTAGTCGGA
3C            CCGGCAGCCAGAGTGTGTGGTCCCGTGTACTGCTTTACTCCCAGCCCCGTCGTAGTCGGA
              ***** ***************** *********************** ************

7A            ACCACTGACCGTCTCGGGGTCCCTACTTACACCTGGGGGGAGAATGAGTCTGATGTCTTT
6B            ACCACTGACCGTCTCGGGGTCCCTACTTACACCTGGGGGGAGAATGAGTCTGATGTCTTC
9D            ACCACTGACCGTCTCGGGGTCCCTACTTACACCTGGGGGGAGAATGAGTCTGATGTCTTT
1D            ACCACTGACCGTCTCGGGGTCCCTACTTACACCTGGGGGGAGAATGAGTCTGATGTCTTT
8E            ACCACTGACCGTCTCGGGGTCCCTACTTACACCTGGGGGGAGAATGAGTCTGATGTCTTT
9A            ACCACTGACCGTCTCGGGGTCCCTACTTACACTTGGGGGGAGAATGAGTCTGATGTCTTT
10D           ACCACTGACCGTCTCGGGGTCCCTACTTACACTTGGGGGGAGAATGAGTCTGATGTCTTT
3D            ACCACTGACCGTCTCGGGGTCCCTACTTACACTTGGGGGGAGAATGAGTCTGATGTCTTT
4D            ACCACTGACCGTCTCGGGGTCCCTACTTACACTTGGGGGGAGAATGAGTCTGATGTCTTT
5D            ACCACTGACCGTCTCGGGGTCCCTACTTACACTTGGGGGGAGAATGAGTCTGATGTCTTT
10B           ACCACTGACCGTCTCGGGGTCCCTACTTACACTTGGGGGGAGAATGAGTCTGATGTCTTT
5E            ACCACTGACCGTCTCGGGGTCCCTACTTACACTTGGGGGGAGAATGAGTCTGATGTCTTT
1E            ACCACTGACCGTCTCGGGGTCCCTACTCACACTTGGGGGGAGAATGAGTCTGATGTCTTT
7D            ACCACTGACCGTCTCGGGGTCCCTACTTACACTTGGGGGGAGAATGAGTCTGATGTCTTT
2D            ACCACTGACCGTCTCGGGGTCCCTACTTACACTTGGGGGGAGAATGAGTCTGATGTCTTT
2E            ACCACTGACCGTCTCGGGGTCCCTACTTACACTTGGGGGGAGAATGAGTCTGATGTCTTT
6E            ACCACTGACCGTCTCGGGGTCCCTACTTACACTTGGGGGGAGAATGAGTCTGATGTCTTT
2B            ACCACTGACCGTCTCGGGGTCCCTACTTACACTTGGGGGGAGAATGAGTCTGATGTCTTT
3B            ACCACTGACCGTCTCGGGGTCCCTACTTACACTTGGGGGGAGAATGAGTCTGATGTCTTT
6D            ACCACTGACCGTCTCGGGGTCCCTACTCACACTTGGGGGGAGAATGAGTCTGATGTCTTT
9C            ACCACTGACCGTCTCGGGGTCCCTACTTACACTTGGGGGGAGAATGAGTCAGATGTCTTT
2C            ACCACTGACCGTCTCGGGGTCCCTACTTACACTTGGGGGGAGAATGAGTCTGATGTCTTT
5A            ACCACTGACCGTCTCGGGGTCCCTACTTACACTTGGGGGGAGAATGAGTCTGATGTCTTT
8A            ACCACTGACCGTCTCGGGGTCCCTACTTACACTTGGGGGGAGAATGAGTCTGATGTCTTT
3E            ACCACTGACCGTCTCGGGGTCCCTACTCACACTTGGGGGGAGAATGAGTCTGATGTCTTT
4E            ACCACTGACCGTCTCGGGGTCCCTACTCACACTTGGGGGGAGAATGAGTCTGATGTCTTT
10A           ACCACTGACCGTCTCGGGGTCCCTACTTACACTTGGGGGGAGAATGAGTCTGATGTCTTT
7B            ACCACTGACCGTCTCGGGGTCCCTACTTACACTTGGGGGGAGAATGAGTCTGATGTCTTT
6C            ACCACTGACCGTCTCGGGGTCCCTACTTACACTTGGGGGGAGAATGAGTCAGATGTCTTT
8C            ACCACTGACCGTCTCGGGGTCCCTACTTACACTTGGGGGGAGAATGAGTCAGATGTCTTT
1C            ACCACTGACCGTCTCGGGGTCCCTACTTACACTTGGGGGGAGAATGAGTCAGATGTCTTT
4C            ACCACTGACCGTCTCGGGGTCCCTACTTACACTTGGGGGGAGAATGAGTCAGATGTCTTT
7C            ACCACTGACCGTCTCGGGGTCCCTACTTACACTTGGGGGGAGAATGAGTCAGATGTCTTT
10C           ACCACTGACCGTCTCGGGGTCCCTACTTACACTTGGGGGGAGAATGAGTCAGATGTCTTT
```

```
5C              ACCACTGACCGTCTCGGGGTCCCTACTTACACTTGGGGGGAGAATGAGTCAGATGTCTTT
3C              ACCACTGACCGTCTCGGGGTCCCTACTTACACTTGGGGGGAGAATGAGTCAGATGTCTTT
                *************************** **** ***************** ******** 

7A              CTTTTGAACTCGACTAGGCCGCCGCGTGGTGCGTGGTTCGGATGCGTGTGGATGAACACT
6B              CTTTTGAACTCGACTAGGCCGCCGCGTGGTGCGTGGTTCGGATGCGTGTGGATGAACACT
9D              CTTTTGAACTCGACTAGGCCGCCGCGTGGTGCGTGGTTCGGATGCGTGTGGATGAACACT
1D              CTTTTGAACTCGACTAGGCCGCCGCGTGGTGCGTGGTTCGGATGCGTGTGGATGAACACT
8E              CTTTTGAACTCGACTAGGCCGCCGCGTGGTGCGTGGTTCGGATGCGTGTGGATGAACACT
9A              CTTTTGAACTCGACTAGGCCGCCGCGTGGTGCGTGGTTCGGGTGCGTGTGGATGAACACT
10D             CTTTTGAACTCGACTAGGCCGCCGCGTGGTGCGTGGTTCGGATGCGTGTGGATGAACACT
3D              CTTTTGAACTCGACTAGGCCGCCGCGTGGTGCGTGGTTCGGATGCGTGTGGATGAACACT
4D              CTTTTGAACTCGACTAGGCCGCCGCGTGGTGCGTGGTTCGGATGCGTGTGGATGAACACT
5D              CTTTTGAACTCGACTAGGCCGCCGCGTGGTGCGTGGTTCGGATGCGTGTGGATGAACACT
10B             CTTTTGAACTCGACTAGGCCGCCGCGTGGTGCGTGGTTCGGATGCGTGTGGATGAACACT
5E              CTTTTGAACTCGACTAGGCCGCCGCGTGGTGCGTGGTTCGGATGCGTGTGGATGAACACT
1E              CTTTTGAACTCGACTAGGCCGCCGCGTGGTGCGTGGTTCGGATGCGTGTGGATGAACACT
7D              CTTTTGAACTCGACTAGGCCGCCGCGTGGTGCGTGGTTCGGGTGCGTGTGGATGAACACT
2D              CTTTTGAACTCGACTAGGCCGCCGCGTGGTGCGTGGTTCGGATGCGTGTGGATGAACACT
2E              CTTTTGAACTCGACTAGGCCGCCGCGTGGTGCGTGGTTCGGATGCGTGTGGATGAACACT
6E              CTTTTGAACTCGACTAGGCCGCCGCGTGGTGCGTGGTTCGGATGCGTGTGGATGAACACT
2B              CTTTTGAACTCGACTAGGCCGCCGCGTGGTGCGTGGTTCGGGTGCGTGTGGATGAACACT
3B              CTTTTGAACTCGACTAGGCCGCCGCGTGGTGCGTGGTTCGGATGCGTGTGGATGAACACT
6D              CTTTTGAACTCGACTAGGCCGCCGCGTGGTGCGTGGTTCGGATGCGTGTGGATGAACACT
9C              CTTTTGAACTCGACTAGGCCGCCGCGTGGTGCGTGGTTCGGATGCGTGTGGATGAACACT
2C              CTTTTGAACTCGACTAGGCCGCCGCGTGGTGCGTGGTTCGGATGCGTGTGGATGAACACT
5A              CTTTTGAACTCGACTAGGCCGCCGCGTGGTGCGTGGTTCGGATGCGTGTGGATGAACACT
8A              CTTTTGAACTCGACTAGGCCGCCGCGTGGTGCGTGGTTCGGATGCGTGTGGATGAACACT
3E              CTTTTGAACTCGACTAGGCCGCCGCGTGGTGCGTGGTTCGGATGCGTGTGGATGAACACT
4E              CTTTTGAACTCGACTAGGCCGCCGCGTGGTGCGTGGTTCGGATGCGTGTGGATGAACACT
10A             CTTTTGAACTCGACTAGGCCGCCGCGTGGTGCGTGGTTCGGATGCGTGTGGATGAACACT
7B              CTTTTGAACTCGACTAGGCCGCCGCGTGGTGCGTGGTTCGGATGCGTGTGGATGAACACT
6C              CTTTTGAACTCGACTAGGCCGCCGCGTGGTGCGTGGTTCGGATGCGTGTGGATGAACACT
8C              CTTTTGAACTCGACTAGGCCGCCGCGTGGTGCGTGGTTCGGATGCGTGTGGATGAACACT
1C              CTTTTGAACTCGACTAGGCCGCCGCGTGGTGCGTGGTTCGGATGCGTGTGGATGAACACT
4C              CTTTTGAACTCGACTAGGCCGCCGCGTGGTGCGTGGTTCGGATGCGTGTGGATGAACACT
7C              CTTTTGAACTCGACTAGGCCGCCGCGTGGTGCGTGGTTCGGATGCGTGTGGATGAACACT
10C             CTTTTGAACTCGACTAGGCCGCCGCGTGGTGCGTGGTTCGGATGCGTGTGGATGAACACT
5C              CTTTTGAACTCGACTAGGCCGCCGCGTGGTGCGTGGTTCGGATGCGTGTGGATGAACACT
3C              CTTTTGAACTCGACTAGGCCGCCGCGTGGTGCGTGGTTCGGATGCGTGTGGATGAACACT
                ***************************************** ******************

7A              ACCGGGTTTACGAAAACCTGCGGCGCTCCTCCATGCGAGGTCAGGGCCAATGCAACCTGG
6B              ACCGGGTTTACGAAAACCTGCGGCGCTCCTCCATGCGAGGTCAGGGCCAATGCAACCTGG
9D              ACCGGGTTTACGAAAACCTGCGGCGCTCCTCCATGCGAGGTCAGGGCCAATACAACCTGG
1D              ACCGGGTTTACGAAAACCTGCGGCGCTCCTCCATGCGAGGTCAGGGCCAATGCAACCTGG
8E              ACCGGGTTTACGAAAACCTGCGGCGCTCCTCCATGCGAGGTCAGGGCCAATGCAACCTGG
9A              ACCGGGTTTACGAAAACCTGCGGTGCTCCTCCATGCGAGGTCAGGGCCAATAGAACCTGG
10D             ACCGGGTTTACGAAAACCTGCGGTGCTCCTCCATGCGAGGTCAGGGCCAATAGAACCTGG
3D              ACCGGGTTTACGAAAACCTGCGGTGCTCCTCCATGCGAGGTCAGGGCCAATAGAACCTGG
4D              ACCGGGTTTACGAAAACCTGCGGTGCTCCTCCATGCGAGGTCAGGGCCAATAGAACCTGG
5D              ACCGGGTTTACGAAAACCTGCGGTGCTCCTCCATGCGAGGTCAGGGCCAATAGAACCTGG
10B             ACCGGGTTTACGAAAACCTGCGGTGCTCCTCCATGCGAGGTCAGGGCCAATAGAACCTGG
5E              ACCGGGTTTACGAAAACCTGCGGTGCTCCTCCATGCGAGGTCAGGGCCAATAGAACCTGG
1E              ACCGGGTTTACGAAAACCTGCGGTGCTCCTCCATGCGAGGTCAGGGCCAATAGAACCTGG
7D              ACCGGGTTTACGAAAACCTGCGGTGCTCCTCCATGCGAGGTCAGGGCCAATAGAACCTGG
2D              ACCGGGTTTACGAAAACCTGCGGTGCTCCTCCATGCGAGGTCAGGGCCAATAGAACCTGG
```

```
2E          ACCGGGTTTACGAAAACCTGCGGTGCTCCTCCATGCGAGGTCAGGGCCAATAGAACCTGG
6E          ACCGGGTTTACGAAAACCTGCGGTGCTCCTCCATGCGAGGTCAGGGCCAATAGAACCTGG
2B          ACCGGGTTTACGAAAACCTGCGGTGCTCCTCCATGCGAGGTCAGGGCCAATAGAACCTGG
3B          ACCGGGTTTACGAAAACCTGCGGTGCTCCTCCATGCGAGGTCAGGGCCAATAGAACCTGG
6D          ACCGGGTTTACGAAAACCTGCGGTGCTCCTCCATGCGAGGTCAGGGCCAATAGAACCTGG
9C          ACCGGGTTTACGAAAACCTGCGGTGCTCCTCCATGCGAGGTCAGGGCCAATAGAACCTGG
2C          ACCGGGTTTACGAAAACCTGCGGTGCTCCTCCATGCGAGGTCAGGGCCAATAGAACCTGG
5A          ACCGGGTTTACGAAAACCTGCGGTGCTCCTCCATGCGAGGTCAGGGCCAATAGAACCTGG
8A          ACCGGGTTTACGAAAACCTGCGGTGCTCCTCCATGCGAGGTCAGGGCCAATAGAGCCTGG
3E          ACCGGGTTTACGAAAACCTGCGGTGCTCCTCCATGCGAGGTCAGGGCCAATAGAACCTGG
4E          ACCGGGTTTACGAAAACCTGCGGTGCTCCTCCATGCGAGGTCAGGGCCAATAGAACCTGG
10A         ACCGGGTTTACGAAAACCTGCGGTGCTCCTCCATGCGAGGTCAGGGCCAATAGAACCTGG
7B          ACCGGGTTTACGAAAACCTGCGGTGCTCCTCCATGCGAGGTCAGGGCCAATAGAACCTGG
6C          ACCGGGTTTACGAAAACCTGCGGTGCTCCTCCATGCGAGGTCAGGGCCAATAGAACCTGG
8C          ACCGGGTTTACGAAAACCTGCGGTGCTCCTCCATGCGAGGTCAGGGCCAATAGAACCTGG
1C          ACCGGGTTTACGAAAACCTGCGGTGCTCCTCCATGCGAGGTCAGGGCCAATAGAACCTGG
4C          ACCGGGTTTACGAAAACCTGCGGTGCTCCTCCATGCGAGGTCAGGGCCAATAGAACCTGG
7C          ACCGGGTTTACGAAAACCTGCGGTGCTCCTCCATGCGAGGTCAGGGCCAATAGAACCTGG
10C         ACCGGGTTTACGAAAACCTGCGGTGCTCCTCCATGCGAGGTCAGGGCCAATAGAACCTGG
5C          ACCGGGTTTACGAAAACCTGCGGTGCTCCTCCATGCGAGGTCAGGGCCAATAGAACCTGG
3C          ACCGGGTTTACGAAAACCTGCGGTGCTCCTCCATGCGAGGTCAGGGCCAATAGAACCTGG
            *********************** ***************************  * *****

7A          CGCTGCCCCACTGACTGCTTCAGGAAACACCCGGAGACTACCTACACCAAATGCGGGTCA
6B          CATTGCCCCACTGACTGCTTCAGGAAACACCCGGAGACTACCTACACCAAATGCGGGTCA
9D          CACTGCCCCACTGACTGCTTCAGGAAACACCCGGAGACTACCTACACCAAATGCGGGTCA
1D          CATTGCCCCACTGACTGCTTCAGGAAACACCCGGAGACTACCTACACCAAATGCGGGTCA
8E          CATTGCCCCACTGACTGCTTCAGGAAACACCCGGAGACTACCTACACCAAATGCGGGTCA
9A          TACTGCCCCACTGACTGCTTCAGGAAACACCCGGAGACTACCTACACCAAATGCGGGTCA
10D         CGCTGCCCCACTGACTGCTTCAGGAAACATCCGGAGACTACCTACGCCAAATGCGGGTCA
3D          CGCTGCCCCACTGACTGCTTCAGAAAACATCCGGAGACTACCTACGCCAAATGCGGGTCA
4D          CGCTGCCCCACTGACTGCTTCAGAAAACATCCGGAGACTACCTACGCCAAATGCGGGTCA
5D          CGCTGCCCCACTGACTGCTTCAGAAAACATCCGGAGACTACCTACGCCAAATGCGGGTCA
10B         CGCTGCCCCACTGACTGCTTCAGAAAACATCCGGAGACTACCTACGCCAAATGCGGGTCA
5E          CGCTGCCCCACTGACTGCTTCAGAAAACATCCGGAGACTACCTACGCCAAATGCGGGTCA
1E          CGCTGCCCCACTGACTGCTTCAGAAAACATCCGGAGACTACCTACGCCAAATGCGGGTCA
7D          CGCTGCCCCACTGACTGCTTCAGAAAACATCCGGAGACTACCTACGCCAAATGCGGGTCA
2D          CGCTGCCCCACTGACTGCTTCAGAAAACATCCGGAGACTACCTACGCCAAATGCGGGTCA
2E          CGCTGCCCCACTGACTGCTTCAGAAAACATCCGGAGACTACCTACGCCAAATGCGGGTCA
6E          CGCTGCCCCACTGACTGCTTCAGAAAACATCCGGAGACTACCTACGCCAAATGCGGGTCA
2B          TACTGCCCCACTGACTGCTTCAGGAAACACCCGGAGACTACCTACACCAAATGCGGGTCA
3B          CGCTGCCCCACTGACTGCTTCAGGAAACATCCGGAGACTACCTACGCCAAATGCGGGTCA
6D          CGCTGCCCCACTGACTGCTTCAGAAAACATCCGGAGACTACCTACGCCAAATGCGGGTCA
9C          CGCTGCCCCACTGACTGCTTCAGAAAACATCCGGAGACTACCTACGCCAAATGCGGGTCA
2C          CGCTGCCCCACTGACTGCTTCAGAAAACATCCGGAGACTACCTACGCCAAATGCGGGTCA
5A          CGCTGCCCCACTGACTGCTTCAGAAAACATCCGGAGACTACCTACGCCAAATGCGGGTCA
8A          CGCTGCCCCACTGACTGCTTCAGAAAACATCCGGAGACTACCTACGCCAAATGCGGGTCA
3E          CGCTGCCCCACTGACTGCTTCAGAAAACATCCGGAGACTACCTACGCCAAATGCGGGTCA
4E          CGCTGCCCCACTGACTGCTTCAGAAAACATCCGGAGACTACCTACGCCAAATGCGGGTCA
10A         CGCTGCCCCACTGACTGCTTCAGAAAACATCCGGAGACTACCTACGCCAAATGCGGGTCA
7B          CGCTGCCCCACTGACTGCTTCAGAAAACATCCGGAGACTACCTACGCCAAATGCGGGTCA
6C          CGCTGCCCCACTGACTGCTTCAGAAAACATCCGGAGACTACCTACGCCAAATGCGGGTCA
8C          CGCTGCCCCACTGACTGCTTCAGAAAACATCCGGAGACTACCTACGCCAAATGCGGGTCA
1C          CGCTGCCCCACTGACTGCTTCAGAAAACATCCGGAGACTACCTACGCCAAATGCGGGTCA
4C          CGCTGCCCCACTGACTGCTTCAGAAAACATCCGGAGACTACCTACGCCAAATGCGGGTCA
7C          CGCTGCCCCACTGACTGCTTCAGAAAACATCCGGAGACTACCTACGCCAAATGCGGGTCA
10C         CGCTGCCCCACTGACTGCTTCAGAAAACATCCGGAGACTACCTACGCCAAATGCGGGTCA
```

```
5C              CGCTGCCCCACTGACTGCTTCAGAAAACATCCGGAGACTACCTACGCCAAATGCGGGTCA
3C              CGCTGCCCCACTGACTGCTTCAGAAAACATCCGGAGACTACCTACGCCAAATGCGGGTCA
                   ******************** ***** *************** **************

7A              GGGCCCTGGATCACACCACGATGCCTGATTCATTATCCATACCGGCTATGGCATTATCCA
6B              GGGCCCTGGATCACACCACGATGCCTGATTCATTATCCATACCGGCTATGGCATTATCCA
9D              GGGCCCTGGATCACACCACGATGCCTGATTCATTATCCATACCGGCTATGGCATTATCCA
1D              GGGCCCTGGATCACACCACGATGCCTGATTCATTATCCATACCGGCTATGGCATTATCCA
8E              GGGCCCTGGATCACACCACGATGCCTGATTCATTATCCATACCGGCTATGGCATTATCCA
9A              GGGCCCTGGATCACACCACGATGCCTGATTCATTATCCATATCGGCTATGGCATTATCCA
10D             GGGCCCTGGATCACACCACGATGCCTGATTCATTATCCATACCGGCTATGGCATTATCCA
3D              GGGCCCTGGATCACACCACGATGCCTGATTCATTATCCATACCGGCTATGGCATTATCCA
4D              GGGCCCTGGATCACACCACGATGCCTGATTCATTATCCATACCGGCTATGGCATTATCCA
5D              GGGCCCTGGATCACACCACGATGCCTGATTCATTATCCATACCGGCTATGGCATTATCCA
10B             GGGCCCTGGATCACACCACGATGCCTGATTCATTATCCATACCGGCTATGGCATTATCCA
5E              GGGCCCTGGATCACACCACGATGCCTGATTCATTATCCATACCGGCTATGGCATTATCCA
1E              GGGCCCTGGATCACACCACGATGCCTGATTCATTATCCATACCGGCTATGGCATTATCCA
7D              GGGCCCTGGATCACACCACGATGCCTGATTCATTATCCATACCGGCTATGGCATTATCCA
2D              GGGCCCTGGATCACACCACGATGCCTGATTCATTATCCATACCGGCTATGGCATTATCCA
2E              GGGCCCTGGATCACACCACGATGCCTGATTCATTATCCATACCGGCTATGGCATTATCCA
6E              GGGCCCTGGATCACACCACGATGCCTGATTCATTATCCATACCGGCTATGGCATTATCCA
2B              GGGCCCTGGATCACACCACGATGCCTGATTCATTATCCATACCGGCTATGGCATTATCCA
3B              GGGCCCTGGATCACACCACGATGCCTGATTCATTATCCATACCGGCTATGGCATTATCCA
6D              GGGCCCTGGATCACACCACGATGCCTGATTCATTATCCATACCGGCTATGGCATTATCCA
9C              GGGCCCTGGATCACACCACGATGCCTGATTCATTATCCATACCGGCTATGGCATTATCCA
2C              GGGCCCTGGATCACACCACGATGCCTGATTCATTATCCATACCGGCTATGGCATTATCCA
5A              GGGCCCTGGATCACACCACGATGCCTGATTCATTATCCATACCGGCTATGGCATTATCCA
8A              GGGCCCTGGATCACACCACGATGCCTGATTCATTATCCATACCGGCTATGGCATTATCCA
3E              GGGCCCTGGATCACACCACGATGCCTGATTCATTATCCATACCGGCTATGGCATTATCCA
4E              GGGCCCTGGATCACACCACGATGCCTGATTCATTATCCATACCGGCTATGGCATTATCCA
10A             GGGCCCTGGATCACACCACGATGCCTGATTCATTATCCATACCGGCTATGGCATTATCCA
7B              GGGCCCTGGATCACACCACGATGCCTGATTCATTATCCATACCGGCTATGGCATTATCCA
6C              GGGCCCTGGATCACACCACGATGCCTGATTCATTATCCATACCGGCTATGGCATTATCCA
8C              GGGCCCTGGATCACACCACGATGCCTGATTCATTATCCATACCGGCTATGGCATTATCCA
1C              GGGCCCTGGATCACACCACGATGCCTGATTCATTATCCATACCGGCTATGGCATTATCCA
4C              GGGCCCTGGATCACACCACGATGCCTGATTCATTATCCATACCGGCTATGGCATTATCCA
7C              GGGCCCTGGATCACACCACGATGCCTGATTCATTATCCATACCGGCTATGGCATTATCCA
10C             GGGCCCTGGATCACACCACGATGCCTGATTCATTATCCATACCGGCTATGGCATTATCCA
5C              GGGCCCTGGATCACACCACGATGCCTGATTCATTATCCATACCGGCTATGGCATTATCCA
3C              GGGCCCTGGATCACACCACGATGCCTGATTCATTATCCATACCGGCTATGGCATTATCCA
                ***************************************** ******************

7A              TGCACCGTCAACTTTACCGTCTTTAAGATCAGGACATTTATTGGCGGTATAGAGCATCGG
6B              TGCACCGTCAACTTTACCATCTTTAAGATCAGGACATTTATTGGCGGTATAGAGCATCGG
9D              TGCACCGTCAACTTTACCGTCTTTAAGATCAGGACATTTATTGGCGGTATAGAGCATCGG
1D              TGCACCGTCAACTTTACCATCTTTAAGATCAGGACATTTATTGGCGGTATAGAGCATCGG
8E              TGCACCGTCAACTTTACCATCTTTAAGATCAGGACATTTATTGGCGGTATAGAGCATCGG
9A              TGCACCGTCAACTTTACCATCTTTAAGATCAGGACATTTATTGGCGGTATAGAGCATCGG
10D             TGCACCGTCAACTTTACCATCTTTAAGATCAGGACATTTGTTGGCGGTATAGAACATCGG
3D              TGCACCGTCAACTTTACCATCTTTAAGATCAGGACATTTGTTGGCGGTATAGAGCATCGG
4D              TGCACCGTCAACTTTACCATCTTTAAGATCAGGACATTTGTTGGCGGTATAGAGCATCGG
5D              TGCACCGTCAACTTTACCATCTTTAAGATCAGGACATTTGTTGGCGGTATAGAGCATCGG
10B             TGCACCGTCAACTTTACCATCTTTAAGATCAGGACATTTGTTGGCGGTATAGAGCATCGG
5E              TGCACCGTCAACTTTACCATCTTTAAGATCAGGACATTTGTTGGCGGTATAGAGCATCGG
1E              TGCACCGTCAACTTTACCATCTTTAAGATCAGGACATTTGTTGGCGGTATAGAGCATCGG
7D              TGCACCGTCAACTTTACCATCTTTAAGATCAGGACATTTGTTGGCGGTATAGAGCATCGG
2D              TGCACCGTCAACTTTACCATCTTTAAGATCAGGACATTTGTTGGCGGTATAGAGCATCGG
```

```
2E              TGCACCGTCAACTTTACCATCTTTAAGATCAGGACATTTGTTGGCGGTATAGAGCATCGG
6E              TGCACCGTCAACTTTACCATCTTTAAGATCAGGACATTTGTTGGCGGTATAGAGCATCGG
2B              TGCACCGTCAACTTTACCATCTTTAAGATCAGGACATTTATTGGCGGTATAGAGCATCGG
3B              TGCACCGTCAACTTTACCATCTTTAAGATCAGGACATTTGTTGGCGGTATAGAACATCGG
6D              TGCACCGTCAACTTTACCATCTTTAAGATCAGGACATTTGTTGGCGGTATAGAGCATCGG
9C              TGCACCGTCAACTTTACCATCTTTAAGATCAGGACATTTGTTGGCGGTATAGAGCATCGG
2C              TGCACCGTCAACTTTACCATCTTTAAGATCAGGACATTTGTTGGCGGTATAGAGCATCGG
5A              TGCACCGTCAACTTTACCATCTTTAAGATCAGGACATTTGTTGGCGGTATAGAGCATCGG
8A              TGCACCGTCAACTTTACCATCTTTAAGATCAGGACATTTGTTGGCGGTATAGAGCATCGG
3E              TGCACCGTCAACTTTACTATCTTTAAGATCAGGACATTTGTTGGCGGTATAGAGCATCGG
4E              TGCACCGTCAACTTTACCATCTTTAAGATCAGGACATTTGTTGGCGGTATAGAGCATCGG
10A             TGCACCGTCAACTTTACCATCTTTAAGATCAGGACATTTGTTGGCGGTATAGAGCATCGG
7B              TGCACCGTCAACTTTACCATCTTTAAGATCAGGACATTTGTTGGCGGTATAGAGCATCGG
6C              TGCACCGTCAACTTTACCATCTTTAAGATCAGGACATTTGTTGGCGGTATAGAGCATCGG
8C              TGCACCGTCAACTTTACCATCTTTAAGATCAGGACATTTGTTGGCGGTATAGAGCATCGG
1C              TGCACCGTCAACTTTACCATCTTTAAGATCAGGACATTTGTTGGCGGTATAGAGCATCGG
4C              TGCACCGTCAACTTTACCATCTTTAAGATCAGGACATTTGTTGGCGGTATAGAGCATCGG
7C              TGCACCGTCAACTTTACCATCTTTAAGATCAGGACATTTGTTGGCGGTATAGAGCATCGG
10C             TGCACCGTCAACTTTACCATCTTTAAGATCAGGACATTTGTTGGCGGTATAGAGCATCGG
5C              TGCACCGTCAACTTTACCATCTTTAAGATCAGGACATTTGTTGGCGGTATAGAGCATCGG
3C              TGCACCGTCAACTTTACCATCTTTAAGATCAGGACATTTGTTGGCGGTATAGAGCATCGG
                ***************** ****************** ************ ******

7A              ATGGAAGCGGCGTGTAATTGGACCAGGGGAGAAGTTTGTGGCTTGGAGCATAGGGATCGC
6B              ATGGAAGCAGCGTGTAATTGGACCAGGGGAGAAGTTTGTGGCTTGGAGCATAGGGATCGC
9D              ATGGAAGCGGCGTGTAATTGGACCAGGGGAGAAGTTTGTGGCTTGGAGCATAGGGATCGC
1D              ATGGAAGCGGCGTGTAATTGGACCAGGGGAGAAGTTTGTGGCTTGGAGCATAGGGATCGC
8E              ATGGAAGCGGCGTGTAATTGGACCAGGGGAGAAGTTTGTGGCTTGGAGCATAGGGATCGC
9A              ATGGAAGCGGCGTGTAATTGGACCAGGGGAGAAGTTTGTGGCTTGGAGCATAGGGATCGC
10D             ATGGAAACGGCGTGTAATTGGACCAGGGGAGAAGTTTGTGGCTTGGAGCATAGGGATCGC
3D              ATGGAAGCGGCGTGTAATTGGACCAGGGGAGAAGTTTGTGGCTTGGAGCATAGGGATCGC
4D              ATGGAAGCGGCGTGTAATTGGACCAGGGGAGAAGTTTGTGGCTTGGAGCATAGGGATCGC
5D              ATGGAAGCGGCGTGTAATTGGACCAGGGGAGAAGTTTGTGGCTTGGAGCATAGGGATCGC
10B             ATGGAAGCGGCGTGTAATTGGACCAGGGGAGAAGTTTGTGGCTTGGAGCATAGGGATCGC
5E              ATGGAAGCGGCGTGTAATTGGACCAGGGGAGAAGTTTGTGGCTTGGAGCATAGGGATCGC
1E              ATGGAAGCGGCGTGTAATTGGACCAGGGGAGAAGTTTGTGGCTTGGAGCATAGGGATCGC
7D              ATGGAAGCGGCGTGTAATTGGACCAGGGGAGAAGTTTGTGGCTTGGAGCATAGGGATCGC
2D              ATGGAAGCGGCGTGTAATTGGACCAGGGGAGAAGTTTGTGGCTTGGAGCATAGGGATCGC
2E              ATGGAAGCGGCGTGTAATTGGACCAGGGGAGAAGTTTGTGGCTTGGAGCATAGGGATCGC
6E              ATGGAAGCGGCGTGTAATTGGACCAGGGGAGAAGTTTGTGGCTTGGAGCATAGGGATCGC
2B              ATGGAAGCGGCGTGTAATTGGACCAGGGGAGAAGTTTGTGGCTTGGAGCATAGGGATCGC
3B              ATGGAAGCGGCGTGTAATTGGACCAGGGGAGAAGTTTGTGGCTTGGAGCATAGGGATCGC
6D              ATGGAAGCGGCATGTAATTGGACCAGGGGAGAAGTTTGTGGCTTGGAGCATAGGGATCGC
9C              ATGGAAGCGGCGTGTAATTGGACCAGGGGAGAAGTTTGTGGCTTGGAGCATAGGGATCGC
2C              ATGGAAGCGGCGTGTAATTGGACCAGGGGAGAAGTTTGTGGCTTGGAGCATAGGGATCGC
5A              ATGGAAGCGGCGTGTAATTGGACCAGGGGAGAAGTTTGTGGCTTGGAGCATAGGGATCGC
8A              ATGGAAGCGGCGTGTAATTGGACCAGGGGAGAAGTTTGTGGCTTGGAGCATAGGGATCGC
3E              ATGGAAGCGGCGTGTAATTGGACCAGGGGAGAAGTTTGTGGCTTGGAGCATAGGGATCGC
4E              ATGGAAGCGGCGTGTAATTGGACCAGGGGAGAAGTTTGTGGCTTGGAGCATAGGGATCGC
10A             ATGGAAGCGGCGTGTAATTGGACCAGGGGAGAAGTTTGTGGCTTGGAGCATAGGGATCGC
7B              ATGGAAGCGGCGTGTAATTGGACCAGGGGAGAAGTTTGTGGCTTGGAGCATAGGGATCGC
6C              ATGGAAGCGGCGTGTAATTGGACCAGGGGAGAAGTTTGTGGCTTGGAGCATAGGGATCGC
8C              ATGGAAGCGGCGTGTAATTGGACCAGGGGAGAAGTTTGTGGCTTGGAGCATAGGGATCGC
1C              ATGGAAGCGGCGTGTAATTGGACCAGGGGAGAAGTTTGTGGCTTGGAGCATAGGGATCGC
4C              ATGGAAGCGGCGTGTAATTGGACCAGGGGAGAAGTTTGTGGCTTGGAGCATAGGGATCGC
7C              ATGGAAGCGGCGTGTAATTGGACCAGGGGAGAAGTTTGTGGCTTGGAGCATAGGGATCGC
10C             ATGGAAGCGGCGTGTAATTGGACCAGGGGAGAAGTTTGTGGCTTGGAGCATAGGGATCGC
```

```
5C              ATGGAAGCGGCGTGTAATTGGACCAGGGGAGAAGTTTGTGGCTTGGAGCATAGGGATCGC
3C              ATGGAAGCGGCGTGTAATTGGACCAGGGGAGAAGTTTGTGGCTTGGAGCATAGGGATCGC
                ****** * ** ************************************************

7A              GTAGAGCTGTCGCCTCTGCTCCTCTCCACCACAACATGGCAAATCCTCCCCTGTTCTTTC
6B              GCAGAGCTGTCACCTTTGCTCCTCTCCACCACAACATGGCAAATCCTCCCCTGTTCTTTC
9D              GTAGAGCTGTCGCCTCTGCTCCTCTCCACCACAACATGGCAAATCCTCCCCTGTTCTTTC
1D              GTAGAGCTGTCGCCTCTGCTCCTCTCCACCACAACATGGCAAATCCTCCCCTGTTCTTTC
8E              GTAGAGCTGTCGCCTCTGCTCCTCTCCACCACAACATGGCAAATCCTCCCCTGTTCTTTC
9A              GCAGAGCTGTCACCCTTGCTCCTCTCCACCACAACATGGCAAGTCCTCCCCTGTTCTTTC
10D             GCAGAGCTGTCACCTTTGCTCCTCTCCACCACAACATGGCAAATCCTCCCCTGTTCTTTC
3D              GCAGAGCTGTCACCTTTGCTCCTCTCCACCACAACATGGCAAATCCTCCCCTGTTCTTTC
4D              GCAGAGCTGTCACCTTTGCTCCTCTCCACCACAACATGGCAAATCCTCCCCTGTTCTTTC
5D              GCAGAGCTGTCACCTTTGCTCCTCTCCACCACAACATGGCAAATCCTCCCCTGTTCTTTC
10B             GCAGAGCTGTCACCTTTGCTCCTCTCCACCACAACATGGCAAATCCTCCCCTGTTCTTTC
5E              GCAGAGCTGTCACCTTTGCTCCTCTCCACCACAACATGGCAAATCCTCCCCTGTTCTTTC
1E              GCAGAGCTGTCACCTTTGCTCCTCTCCACCACAACATGGCAAATCCTCCCCTGTTCTTTC
7D              GCAGAGCTGTCACCTTTGCTCCTCTCCACCACAACATGGCAAATCCTCCCCTGTTCTTTC
2D              GCAGAGCTGTCACCTTTGCTCCTCTCCACCACAACATGGCAAATCCTCCCCTGTTCTTTC
2E              GCAGAGCTGTCACCTTTGCTCCTCTCCACCACAACATGGCAAATCCTCCCCTGTTCTTTC
6E              GCAGAGCTGTCACCTTTGCTCCTCTCCACCACAACATGGCAAATCCTCCCCTGTTCTTTC
2B              GCAGAGCTGTCACCCTTGCTCCTCTCCACCACAACATGGCAAATCCTCCCCTGTTCTTTC
3B              GCAGAGCTGTCACCTTTGCTCCTCTCCACCACAACATGGCAAATCCTCCCCTGTTCTTTC
6D              GCAGAGCTGTCACCTTTGCTCCTCTCCACCACAACATGGCAAATCCTCCCCTGTTCTTTC
9C              GCAGAGCTGTCACCTTTGCTCCTCTCCACCACAACATGGCAAATCCTCCCCTGTTCTTTC
2C              GCAGAGCTGTCACCTTTGCTCCTCTCCACCACAACATGGCAAATCCTCCCCTGTTCTTTC
5A              GCAGAGCTGTCACCTTTGCTCCTCTCCACCACAACATGGCAAATCCTCCCCTGTTCTTTC
8A              GCAGAGCTGTCACCTTTGCTCCTCTCCACCACAACATGGCAAATCCTCCCCTGTTCATTC
3E              GCAGAGCTGTCACCTTTGCTCCTCTCCACCACAACATGGCAAATCCTCCCCTGTTCTTTC
4E              GCAGAGCTGTCACCTTTGCTCCTCTCCACCACAACATGGCAAATCCTCCCCTGTTCTTTC
10A             GCAGAGCTGTCACCTTTGCTCCTCTCCACCACAACATGGCAAATCCTCCCCTGTTCTTTC
7B              GCAGAGCTGTCACCTTTGCTCCTCTCCACCACAACATGGCAAATCCTCCCCTGTTCTTTC
6C              GCAGAGCTGTCACCTCTGCTCCTCTCCACCACAACATGGCAAATCCTCCCCTGTTCTTTC
8C              GCAGAGCTGTCACCTCTGCTCCTCTCCACCACAACATGGCAAATCCTCCCCTGTTCTTTC
1C              GCAGAGCTGTCACCTCTGCTCCTCTCCACCACAACATGGCAAATCCTCCCCTGTTCTTTC
4C              GCAGAGCTGTCACCTCTGCTCCTCTCCACCACAACATGGCAAATCCTCCCCTGTTCTTTC
7C              GCAGAGCTGTCACCTCTGCTCCTCTCCACCACAACATGGCAAATCCTCCCCTGTTCTTTC
10C             GCAGAGCTGTCACCTCTGCTCCTCTCCACCACAACATGGCAAATCCTCCCCTGTTCTTTC
5C              GCAGAGCTGTCACCTCTGCTCCTCTCCACCACAACATGGCAAATCCTCCCCTGTTCTTTC
3C              GCAGAGCTGTCACCTCTGCTCCTCTCCACCACAACATGGCAAATCCTCCCCTGTTCTTTC
                * ********* **  ************************** ************* ***

7A              ACCACCTTGCCTGCCCTTTCTACCGGCTTGATCCACCTCCACCAAAATATCGTGGACGTC
6B              ACCACCTTGCCTGCCCTTTCTACCGGCTTGATCCACCTCCACCAAAATATCGTGGACGTC
9D              ACCACCTTGCCTGCCCTTTCTACCGGCTTGATCCACCTCCACCAAAATATCGTGGACGTC
1D              ACCACCTTGCCTGCCCTTTCTACCGGCTTGATCCACCTCCACCAAAATATCGTGGACGTC
8E              ACCACCTTGCCTGCCCTTTCTACCGGCTTGATCCACCTCCACCAAAATATCGTGGACGTC
9A              ACCACCTTGCCTGCCCTTTCTACCGGCTTGATCCACCTCCACCAAAATATCGTGGACGTC
10D             ACCACCTTGCCTGCCCTTTCTACCGGCTTGATCCACCTCCACCAAAATATCGTGGACGTC
3D              ACCACCTTGCCTGCCCTTTCTACCGGCTTGATCCACCTCCACCAAAATATCGTGGACGTC
4D              ACCACCTTACCTGCCCTTTCTACCGGCTTGATCCACCTCCACCAAAATATCGTGGACGTC
5D              ACCACCTTACCTGCCCTTTCTACCGGCTTGATCCACCTCCACCAAAATATCGTGGACGTC
10B             ACCACCTTGCCTGCCCTTTCTACCGGCTTGATCCACCTCCACCAAAATATCGTGGACGTC
5E              ACCACCTTGCCTGCCCTTTCTACCGGCTTGATCCACCTCCACCAAAATATCGTGGACGTC
1E              ACCACCTTGCCTGCCCTTTCTACCGGCTTGATCCACCTCCACCAAAATATCGTGGACGTC
7D              ACCACCTTGCCTGCCCTTTCTACCGGCTTGATCCACCTCCACCAAAATATCGTGGACGTC
2D              ACCACCTTGCCTGCCCTTTCTACCGGCTTGATCCACCTCCACCAAAATATCGTGGACGTC
```

```
2E          ACCACCTTGCCTGCCCTTTCTACCGGCTTGATCCACCTCCACCAAAATATCGTGGACGTC
6E          ACCACCTTGCCTGCCCTTTCTACCGGCTTGATCCACCTCCACCAAAATATCGTGGACGTC
2B          ACCACCTTGCCTGCCCTTTCTACCGGCTTGATCCACCTCCACCAAAATATCGTGGACGTC
3B          ACCACCTTGCCTGCCCTTTCTACCGGCTTGATCCACCTCCACCAAAATATCGTGGACGTC
6D          ACCACCTTGCCTGCCCTTTCTACCGGCTTGATCCACCTCCACCAAAATATCGTGGACGTC
9C          ACCACCTTGCCTGCCCTTTCTACCGGCTTGATCCACCTCCACCAAAATATCGTGGACGTC
2C          ACCACCTTGCCTGCCCTTTCTACCGGCTTGATCCACCTCCACCAAAATATCGTGGACGTC
5A          ACCACCTTGCCTGCCCTTTCTACCGGCTTGATCCACCTCCACCAAAATATCGTGGACGTC
8A          ACCACCTTGCCTGCCCTTTCTACCGGCTTGATCCACCTCCACCAAAATATCGTGGACGTC
3E          ACCACCTTGCCTGCCCTTTCTACCGGCTTGATCCACCTCCACCAAAATATCGTGGACGTC
4E          ACCACCTTGCCTGCCCTTTCTACCGGCTTGATCCACCTCCACCAAAATATCGTGGACGTC
10A         ACCACCTTGCCTGCCCTTTCTACCGGCTTGATCCACCTCCACCAAAATATCGTGGACGTC
7B          ACCACCTTGCCTGCCCTTTCTACCGGCTTGATCCACCTCCACCAAAATATCGTGGACGTC
6C          ACCACCTTGCCTGCCCTTTCTACCGGCTTGATCCACCTCCACCAAAATATCGTGGACGTC
8C          ACCACCTTGCCTGCCCTTTCTACCGGCTTGATCCACCTCCACCAAAATATCGTGGACGTC
1C          ACCACCTTGCCTGCCCTTTCTACCGGCTTGATCCACCTCCACCAAAATATCGTGGACGTC
4C          ACCACCTTGCCTGCCCTTTCTACCGGCTTGATCCACCTCCACCAAAATATCGTGGACGTC
7C          ACCACCTTGCCTGCCCTTTCTACCGGCTTGATCCACCTCCACCAAAATATCGTGGACGTC
10C         ACCACCTTGCCTGCCCTTTCTACCGGCTTGATCCACCTCCACCAAAATATCGTGGACGTC
5C          ACCACCTTGCCTGCCCTTTCTACCGGCTTGATCCACCTCCACCAAAATATCGTGGACGTC
3C          ACCACCTTGCCTGCCCTTTCTACCGGCTTGATCCACCTCCACCAAAATATCGTGGACGTC
            ******** ***************************************************

7A          CAGTACCTTTATGGTGTTGGATCTGCGGTGGTATCTTGGGCCCTAAAGTGGGAATATGTG
6B          CAGTACCTTTATGGTGTTGGATCTGCGGTGGTATCTTGGGCCCTAAAGTGGGAATATGTG
9D          CAGTACCTTTATGGTGTTGGATCTGCGGTGGTATCTTGGGCCCTAAGGTGGGAATATGTG
1D          CAGTACCTTTATGGTGTTGGATCTGCGGTGGTATCTTGGGCCCTAAGGTGGGAATATGTG
8E          CAGTACCTTTATGGTGTTGGATCTGCGGTGGTATCTTGGGCCCTAAGGTGGGAATATGTG
9A          CAGTACCTTTATGGTGTTGGATCTGCAGTGGTATCTTGGGCCCTAAGGTGGGAATATGTG
10D         CAGTACCTTTATGGTGTTGGATCTGCGGTGGTATCTTGGGCCCTAAAGTGGGAATATGTG
3D          CAGTACCTTTATGGTGTTGGATCTGCGGTGGTATCTTGGGCCCTAAGGTGGGAATATGTG
4D          CAGTACCTTTATGGTGTTGGATCTGCGGTGGTATCTTGGGCCCTAAGGTGGGAATATGTG
5D          CAGTACCTTTATGGTGTTGGATCTGCGGTGGTATCTTGGGCCCTAAGGTGGGAATATGTG
10B         CAGTACCTTTATGGTGTTGGATCTGCGGTGGTATCTTGGGCCCTAAGGTGGGAATATGTG
5E          CAGTACCTTTATGGTGTTGGATCTGCGGTGGTATCTTGGGCCCTAAGGTGGGAATATGTG
1E          CAGTACCTTTATGGTGTTGGATCTGCGGTGGTATCTTGGGCCCTAAGGTGGGAATATGTG
7D          CAGTACCTTTATGGTGTTGGATCTGCGGTGGTATCTTGGGCCCTAAGGTGGGAATATGTG
2D          CAGTACCTTTATGGTGTTGGATCTGCGGTGGTATCTTGGGCCCTAAGGTGGGAATATGTG
2E          CAGTACCTTTATGGTGTTGGATCTGCGGTGGTATCTTGGGCCCAGAGGTGGGAATATGTG
6E          CAGTACCTTTATGGTGTTGGATCTGCGGTGGTATCTTGGGCCCAAAGGTGGGAATATGTG
2B          CAGTACCTTTATGGTGTTGGATCTGCGGTGGTATCTTGGGCCCTAAGGTGGGAATATGTG
3B          CAGTACCTTTATGGTGTTGGATCTGCGGTGGTATCTTGGGCCCAGAGGTGGGAATATGTG
6D          CAGTATCTTTATGGTGTTGGATCTGCGGTGGTATCTTGGGCCCTAAGGTGGGAATATGTG
9C          CAGTACCTTTATGGTGTTGGATCTGCGGTGGTATCTTGGGCCCTAAGGTGGGAATATGTG
2C          CAGTACCTTTATGGTGTTGGATCTGCGGTGGTATCTTGGGCCCGGAGGTGGGAATATGTG
5A          CAGTACCTTTATGGTGTTGGATCTGCGGTGGTATCTTGGGCCCTAAGGTGGGAATATGTG
8A          CAGTACCTTTATGGTGTTGGATCTGCGGTGGTATCTTGGGCCCGGAGGTGGGAATATGTG
3E          CAGTATCTTTATGGTGTTGGATCTGCGGTGGTATCTTGGGCCCTAAGGTGGGAATATGTG
4E          CAGTATCTTTATGGTGTTGGATCTGCGGTGGTATCTTGGGCCCTAAGGTGGGAATATGTG
10A         CAGTACCTTTATGGTGTTGGATCTGCGGTGGTATCTTGGGCCCTAAGGTGGGAATATGTG
7B          CAGTACCTTTATGGTGTTGGATCTGCGGTGGTATCTTGGGCCCTAAGGTGGGAATATGTG
6C          CAGTACCTTTATGGTGTTGGATCTGCGGTGGTATCTTGGGCCCGGAGGTGGGAATATGTG
8C          CAGTACCTTTATGGTGTTGGATCTGCGGTGGTATCTTGGGCCCTAAGGTGGGAATATGTG
1C          CAGTACCTTTATGGTGTTGGATCTGCGGTGGTATCTTGGGCCCTAAGGTGGGAATATGTG
4C          CAGTACCTTTATGGTGTTGGATCTGCGGTGGTATCTTGGGCCCTAAGGTGGGAATATGTG
7C          CAGTACCTTTATGGTGTTGGATCTGCGGTGGTATCTTGGGCCCTAAGGTGGGAATATGTG
10C         CAGTACCTTTATGGTGTTGGATCTGCGGTGGTATCTTGGGCCCTAAGGTGGGAATATGTG
```

```
5C              CAGTACCTTTATGGTGTTGGATCTGCGGTGGTATCTTGGGCCCTAAGGTGGAATATGTG
3C              CAGTACCTTTATGGTGTTGGATCTGCGGTGGTATCTTGGGCCCTAAGGTGGAATATGTG
                ***** ******************** ***************  * *************

7A              GTGCTCGCGTTCCTGCTTCTTGCAGACGCGAGAGTCTCCCGCTGCCTATGAT
6B              GTGCTTGCGTTCCTGCTTCTTGCAGACGCGAGAGTCTCCCGCTGCCTATGGA
9D              GTGCTTGCGTTCCTGCTTCTTGCAGACGCGAGAGTCTCCCGCTGCCTATGAT
1D              GTGCTCGCGTTCCTGCTTCTTGCAGACGCGAGAGTCTCCCGCTGCCTATGAT
8E              GTGCTCGCGTTCCTGCTTCTTGCAGACGCGAGAGTCTCCCGCTGCCTATGAT
9A              GTGCTCGCGTTCCTGCTTCTTGCAGACGCGAGAGTCTCCCGCTGCCTATGAT
10D             GTGCTTGCGTTCCTGCTTCTTGCAGACGCGAGAGTCTCCCGCTGCCTATGAT
3D              GTGCTTGCGTTCCTGCTTCTTGCAGACGCGAGAGTCTCCCGCTGCCTATGAT
4D              GTGCTTGCGTTCCTGCTTCTTGCAGACGCGAGAGTCTCCCGCTGCCTATGAT
5D              GTGCTTGCGTTCCTGCTTCTTGCAGACGCGAGAGTCTCCCGCTGCCTATGAT
10B             GTGCTTGCGTTCCTGCTTCTTGCAGACGCGAGAGTCTCCCGCTGCCTATGAT
5E              GTGCTTGCGTTCCTGCTTCTTGCAGACGCGAGAGTCTCCCGCTGCCTATGAT
1E              GTGCTTGCGTTCCTGCTTCTTGCAGACGCGAGAGTCTCCCGCTGCCTATGAT
7D              GTGCTTGCGTTCCTGCTTCTTGCAGACGCGAGAGTCTCCCGCTGCCTATGAT
2D              GTGCTTGCGTTCCTGCTTCTTGCAGACGCGAGAGTCTCCCGCTGCCTATGAT
2E              GTGCTTGCGTTCCTGCTTCTTGCAGACGCGAGAGTCTCCCGCTGCCTATGAT
6E              GTGCTTGCGTTCCTGCTTCTTGCAGACGCGAGAGTCTCCCGCTGCCTATGAT
2B              GTGCTCGCGTTCCTGCTTCCTGCAGACGCGAGAGTCTCCCGCTGCCTATGAT
3B              GTGCTTGCGTTCCTGCTTCTTGCAGACGCGAGAGTCTCCCGCTGCCTATGAT
6D              GTGCTTGCGTTCCTGCTTCCTGCAGACGCGAGAGTCTCCCGCTGCCTATGAT
9C              GTGCTTGCGTTCCTGCTTCTTGCAGACGCGAGAGTCTCCCGCTGCCTATGAT
2C              GTGCTTGCGTTCCTGCTTCTTGCAGACGCGAGAGTCTCCCGCTGCCTATGAT
5A              GTGCTTGCGTTCCTGCTTCCTGCAGACGCGAGAGTCTCCCGCTGCCTATGAT
8A              GTGCTTGCGTTCCTGCTTCTTGCAGACGCGAGAGTCTCCCGCTGCCTATGAT
3E              GTGCTTGCGTTCCTGCTTCTTGCAGACGCGAGAGTCTCCCGCTGCCTATGAT
4E              GTGCTTGCGTTCCTGCTTCTTGCAGACGCGAGAGTCTCCCGCTGCCTATGAT
10A             GTGCTTGCGTTCCTGCTTCTTGCAGACGCGAGAGTCTCCCGCTGCCTATGAT
7B              GTGCTTGCGTTCCTGCTTCTTGCAGACGCGAGAGTCTCCCGCTGCCTATGAT
6C              GTGCTTGCGTTCCTGCTTCTTGCAGACGCGAGAGTCTCCCGCTGCCTATGAT
8C              GTGCTTGCGTTCCTGCTTCTTGCAGACGCGAGAGTCTCCCGCTGCCTATGAT
1C              GTGCTTGCGTTCCTGCTTCTTGCAGACGCGAGAGTCTCCCGCTGCCTATGGA
4C              GTGCTTGCGTTCCTGCTTCTTGCAGACGCGAGAGTCTCCCGCTGCCTATGAT
7C              GTGCTTGCGTTCCTGCTTCTTGCAGACGCGAGAGTCTCCCGCTGCCTATGAT
10C             GTGCTTGCGTTCCTGCTTCTTGCAGACGCGAGAGTCTCCCGCTGCCTATGAT
5C              GTGCTTGCGTTCCTGCTTCTTGCAGACGCGAGAGTCTCCCGCTGCCTATGAT
3C              GTGCTTGCGTTCCTGCTTCTTGCAGACGCGAGAGTCTCCCGCTGCCTATGAT
                ***** ************* ******************************
```
